# Supplementary material for: A Strategy Based on GC-MS/MS, UPLC-MS/MS and Virtual Molecular Docking for Analysis and Prediction of Bioactive Compounds in Eucalyptus Globulus Leaves
Source: Int J Mol Sci. 2019 Aug 8;20(16):3875. doi: 10.3390/ijms20163875 (PMC6721025; doi:10.3390/ijms20163875)
Supplement: Supplementary file 1 [file ijms-20-03875-s001.zip › ijms-541543-final sup/Supplement 1.pdf]

前　言

林木种子检验是为育苗、造林提供有关种子质量确切信息的一项重要工作。70年代后期，根据当时林业生产的需要，主要参照国际种子检验协会（ISTA）1976年《国际种子检验规程》，结合我国林木种子生产实际，经过多年试验，制定了GB 2772—1981《林木种子检验方法》。

GB 2772—1981发布实施后，各级林业部门都相继建立了种子检验机构并开展此项工作。通过检验，使生产用种质量有了保证，对营造速生丰产林、巩固造林绿化成果，推动林业事业的发展起到了积极的作用。

由于林木种子管理水平和生产技术的提高，GB 2772—1981已不适应新形势的需要。为使修订后的标准与国际接轨，根据ISTA1993年《国际种子检验规程》正文及其附件的内容对该标准进行了修订。修订后的标准在技术内容与编写规则上与该国际规程等效。

在对GB 2772—1981进行修订时，着重与国际规程规定一致。依据其内容，将该标准《林木种子检验方法》更名为《林木种子检验规程》。在本规程中引用了X射线测定和质量检验证书两章的内容；其他各章，则在原标准内容结构基础上作了引用。如在抽样章，引用了仪器、送检样品的最低重量和抽样方法等内容；在净度分析章，引用了定义、仪器、两个半试样等内容；在发芽测定章，引用定义的内容，发芽标准由芽提高到幼苗，使检验的发芽结果更贴近田间发芽实际，同时介绍了幼苗的基本结构，规定了正常苗和不正常苗的标准及称量发芽测定法，原标准发芽测定技术规定有118个树种，此次修订时调减4个树种，另新增加35个树种，共计149个树种。新增加树种中，既有乔木，也有灌木，既有用材林树种，也有经济林木，既有荒山造林环境保护树种，又有绿化美化树种；生活力测定章，引用了应用范围、原理的内容，增加了48个属种种子的测定技术条件，比原标准生活力测定增加了26个树种；原种子病虫害感染程度测定更名为种子健康状况测定，该章增加了定义、原则和程序等内容；在含水率测定章，引用了定义、原则、仪器和程序等内容；在重量测定章，引用了原则和仪器等内容。在优良度测定章，参照上述各章引用的内容结构，增加了定义、原则、测定用工具和程序等内容，同时删去原标准中的挤压法，鉴定优良度的树种由53个增加至130。对原标准的附表，按GB/T 1.1—1993的规定，处理为附录。种批和样品重量表、发芽测定技术条件表、生活力测定技术条件表、四唑、靛蓝染色示意图、优良种子鉴别表、检验申请表、净度分析记录表、发芽测定记录表、生活力测定记录表、优良度测定记录表、种子健康状况测定记录表、含水率测定记录表、重量测定记录表、以及增加的种子样品质量检验证书、种批质量检验证书，为标准的附录；检验情况综合表和乔灌木种子示意图为提示的附录。此外，还保留了GB 2772—1981中实践证明适合我国情况又不妨碍国际通用的章节，如生活力测定中的靛蓝测定和优良度测定。

本标准是为造林绿化主要树种制定的，但从操作程序和检验方法看，即使缺少某个细节，原则上仍适用于标准中没有提及的林木种子。

本标准从实施之日起，同时代替GB 2772—1981。

本标准的附录A、附录B、附录C、附录D都是标准的附录。

本标准的附录E和附录F是提示的附录。

本标准由国家林业局提出。

本标准由全国林木种子标准化技术委员会归口。

本标准起草单位：中国林业科学研究院林业研究所、南京林业大学、江西省林业科学研究所、东北林业大学，四川、浙江、福建、山西、甘肃、黑龙江、内蒙古、辽宁、北京林木种苗站。

本标准主要起草人：于淑兰、陈幼生、赵德铭、杨国华、吴琼美、翁尧富、陈恩军、李锦文、李庆梅。

《国际种子检验规程》前言

农业最大的风险之一是播下的种子没有生产能力，不能使所需的栽培品种丰产。人们发展种子检验事业，就是要在播种前评定种子品质，使这种风险减小到最低程度。种子品质概念是由不同属性组成的。种子行业的各个方面——种子生产人员、加工人员、仓库管理人员、商人、农民、签证机关以及负责种子管理的政府部门或办事机构都深切关注这些属性。无论是什么情况，检验的最终目的是确定种子的播种价值。

种子是有生命的生物产品，它的行为表现不能准确地加以预测。准确地预测行为表现，那是检验无生命材料即非生物材料才能做到的。种子检验所用的方法必须以所掌握的种子的科学知识和种子分析人员积累的经验为基础，所要求的精确程度和重现性取决于检验的目的。

下面的文本规定了处理国际贸易时评定种子所用的标准定义和方法。这个目的要求有很高的准确性和重现性。种子交换跨越国界时，它有可能在不同国家的实验室接受检验。因而重要的一点便是，所有的实验室都应采用标准的方法。制定这些标准方法原本就是为了在可以接受的范围内得到普遍一致的结果。

本文本分两部分——规程和附件。

规程部分规定了每个检验项目的目的、原则和适用的定义，并概括地规定了应当采用的程序和方法。

附件部分对定义加以扩展，并对规程规定的程序和方法作了详细的描述。

本规程开列的检验项目如果要用本协会的国际种子检验证书填报检验结果，就必须严格遵守本规程，这是强制性的要求，而且对规程任一条款的解释都必须同该章相应附件扩展的内容相符。

在一个国家范围之内处理种子商贸事务，实施种子质量管理的国家法规时，建议尽可能采用本规程和附件。虽然这时并不一定需要签发国际种子检验证书，但是应该明白，如果偏离国际公认的这个文本，会阻碍国家之间的种子自由流通。

考虑到播种季节、土壤类型和海拔高度等因素，种子的送检者可能会要求作咨询性质的检验，希望就这些特殊目的而评定种批的价值。对于这类检验，本规程和附件也能提供基本依据，还可以参照有关文献介绍的其他技术更好地满足这类检验的特殊要求。

本规程和附件是为世界上的主要作物制定的，尽管不是在每个细节上，但是在原则上也适用于文本中没有提及的其他任何栽培物种。

中华人民共和国国家标准

GB 2772—1999

代替 GB 2772—1981

林木种子检验规程

Rules for forest tree seed testing

1 范围

本标准规定了造林绿化树种种子检验的抽样、净度分析、发芽测定、生活力测定、优良度测定、种子健康状况测定、含水量测定、重量测定以及X射线测定的原则和方法，还规定了质量检验证书的内容和格式。

本标准适用于林木种子生产者、经营管理者和使用者在种子采收、调运、播种、贮藏以及国内外贸易时所进行的种子质量的检验。

2 引用标准

下列标准所包含的条文，通过在本标准中引用而构成为本标准的条文。本标准出版时，所示版本均为有效。所有标准都会被修订，使用本标准的各方应探讨使用下列标准最新版本的可能性。

GB 7908—1999 林木种子质量分级

GB/T 8170—1987 数值修约规则

3 抽样

3.1 目的

抽样是抽取有代表性的、数量能满足检验需要的样品，其中某个成分存在的概率仅仅取决于该成分在该种批中出现的水平。

为使种子检验获得正确结果并具有重演性，必须按照本规程规定的方法，从种批中随机提取具有代表性的初次样品、混合样品和送检样品。这是因为同它应当代表的种批相比，样品的数量极少，无论检验工作做得如何准确，检验结果也只能表明供检样品的品质。因此必须尽最大努力保证送检样品能准确地代表该批种子的组成成分。同样，检验机构也要使分取的测定样品能代表送检样品。只有这样才能通过样品的检验评定种批品质。

3.2 定义

3.2.1 种批

具备下列条件的同一树种的种子：

a）在一个县范围内采集的；

b）采种期相同；

c）加工调制和贮藏方法相同；

d）种子经过充分混合，使组成种批的各成分均匀一致地随机分布；

e）不超过规定数量。特大粒种子如核桃、板栗、麻栎、油桐等为10 000 kg；大粒种子如油茶、山杏、苦楝等为5 000 kg；中粒种子如红松、华山松、樟树、沙枣等为3 500 kg；小粒种子如油松、落叶松、杉木、刺槐等为1 000 kg；特小粒种子如桉、桑、泡桐、木麻黄等为250 kg。重量超过规定5%时需另划种批。

国家质量技术监督局1999-11-10批准　　2000-04-01实施

3.2.2 初次样品

从种批的一个抽样点上取出的少量样品。

3.2.3 混合样品

从一个种批中抽取的全部大体等量的初次样品合并混合而成的样品。

3.2.4 送检样品

送交检验机构的样品，可以是整个混合样品，也可以是从中随机分取的一部分，但数量不得少于附录A表A1规定的最低量(见3.3.3)。

3.2.5 测定样品

从送检样品中分取，供作某项品质测定用的样品。

3.3 种批的抽样程序

3.3.1 原则

3.3.1.1 抽样要由受过抽样训练具有经验的人员担任，按本章规定的程序和方法进行抽样。

3.3.1.2 抽样人员在抽样前应查看采种登记表和有关堆装和混合的情况。所有容器都必须具备标签并标记种批号。种批各容器或各部分的排列应便于抽样。

3.3.1.3 抽样时，应当确有证据证明该种批已经充分混拌均匀。如果种批很不均匀，抽样人员能看出袋间或初次样品间的差异时，应拒绝抽样，直至重新混合均匀后再行抽样。

3.3.1.4 初次样品混合前，须检查每个初次样品的种子真实性，检验在混杂程度、含水量、颜色、光泽、气味以及其他品质表现方面是否一致。如初次样品间没有很大差别，可以认为该批种子是均匀一致的，可混合成混合样品。

3.3.1.5 混合样品的大小取决于批量大小。批量愈大，混合样品也愈大。

3.3.1.6 送检样品可按3.4.2的方法将混合样品缩减到适当的大小而得；如混合样品的大小已适当，则不必缩减，直接作为送检样品。

3.3.1.7 一个种批抽取一个送检样品，并按附录C(标准的附录)中表C1填写检验申请表。

3.3.2 抽样强度

3.3.2.1 袋装(或大小一致、容量相近的其他容器盛装)的种批，下列抽样强度应视为最低要求：

5袋以下	每袋都抽，且至少取5个初次样品
6～30袋	抽5袋，或者每3袋抽取1袋，这两种抽样强度中以数量大的一个为准
31～400袋	抽10袋，或者每5袋抽取1袋，这两种抽样强度中以数量大的一个为准
401袋或以上	抽80袋，或者每7袋抽取1袋，这两种抽样强度中以数量大的一个为准

3.3.2.2 从其他类型的容器，或者从倾卸装入容器时的流动种子中抽取样品时，下列抽样强度应视为最低要求：

种批量	应当抽取的初次样品数
500 kg以下	至少5个初次样品
501～3 000 kg	每300 kg一个初次样品，但不少于5个初次样品
3 001 ～20 000 kg	每500 kg一个初次样品，但不少于10个初次样品
20 000 kg以上	每700 kg一个初次样品，但不少于40个初次样品

3.3.3 送检样品的重量

3.3.3.1 净度测定样品一般至少应含2 500粒纯净种子。送检样品的重量至少应为净度测定样品的2～3倍，大粒种子重量至少应为1 000 g，特大粒种子至少要有500粒，详见附录A(标准的附录)，未列入表A1中的树种，可对比千粒重等情况参照表中相应树种确定。

3.3.3.2 种子健康状况测定用的送检样品重量至少为3.3.3.1规定的送检样品的一半。含水量测定的送检样品，最低重量为50 g，需要切片的种类为100 g。

3.3.3.3 检验机构收到的送检样品少于规定数量时，应通知送检单位补送。确因种子价格昂贵，送检样

品少于规定数量时，检验机构也可以尽可能完成检验，但应在质量检验证书上注明“送检样品重量仅××克，不符合规程要求”。

3.3.3.4　送检样品要按种批做好标志，防止混杂。

3.3.4　初次样品的抽取

初次样品的抽取方法关系着样品的代表性。遵从随机原则，采用正确的抽样技术，可以减少误差，提高样品的代表性。

从每个取样的容器中，或从容器的各个部位，或从散装大堆的各个部位扦取重量大体上相等的初次样品。

装在容器（包括袋装）中的种批，应在整个种批中随机选定取样的容器。从选定的容器的上、中、下各部位扦取初次样品，但不一定要求每袋都抽取一个以上部位。种子是散装或在大型容器里的，应随机从各个部位及深度扦取初次样品。

对于不易流动的粘滞性种子，可徒手取得初次样品。

对于装在小型或防湿容器（如铁罐或塑料袋）中的种子，如有可能，应在种子装入容器前或装入容器时扦样。如没有这样做，则应把足够数量的容器打开或穿孔取得初次样品。然后将扦样后的容器封闭或将种子装入新的容器。

3.3.5　混合样品的取得

如果初次样品外观一致，可将其合并混合成混合样品。

3.3.6　送检样品的取得

用3.4.2中的方法之一，将混合样品缩减至适当样品大小而取得。

3.3.7　送检样品的发送

送检样品用木箱、布袋等容器密封包装。加工时种翅不易脱落的种子，需用木箱等硬质容器盛装，以免因种翅脱落增加夹杂物的比例。供含水量测定的和经过干燥含水量很低的送检样品要装在可以密封的防潮容器内，并尽量排出其中空气。种子健康状况测定用的送检样品应装在玻璃瓶或塑料瓶内。

送检样品必须填写两份标签，注明树种、检验申请表（见附录C表C1）编号和种批号，一份放入袋内，另一份挂在袋外。送检样品要尽快连同检验申请表寄送种子检验机构。

3.4　实验室的抽样方法

3.4.1　测定样品的最低重量

各个检验项目测定样品的最低重量在本规程有关章节中做出规定。

3.4.2　测定样品的取得

测定样品应对送检样品有最大的代表性，测定样品的数量应略多于规定数量。取得测定样品的方法是将送检样品充分混合并反复对半分取。以下两个方法可以选用：

a）四分法

将种子均匀地倒在光滑清洁的桌面上，略成正方形。两手各拿一块分样板，从两侧略微提高地把种子拨到中间，使种子堆成长方形，再将长方形两端的种子拨到中央，这样重复3～4次，使种子混拌均匀。将混拌均匀的种子铺成正方形，大粒种子厚度不超过10 cm，中粒种子厚度不超过5 cm，小粒种子厚度不超过3 cm。用分样板沿对角线把种子分成四个三角形，将对顶的两个三角形的种子装入容器中备用，取余下的两个对顶三角形的种子再次混合，按前法继续分取，直至取得略多于测定样品所需数量为止。

b）分样器法

适用于种粒小的、流动性大的种子。分样前先将送检样品通过分样器，使种子分成重量大约相等的两份。两份种子重量相差不超过两份种子平均重的5%时，可以认为分样器是正确的，可以使用；如超过5%，应调整分样器。

分样时先将送检样品通过分样器三次，使种子充分混合后再分取样品，取其中的一份继续用分样器分取，直到种子缩减至略多于测定样品的需要量为止。

3.5 样品保存

3.5.1 种子检验机构收到送检样品后，要按附录E表E1登记，并即进行检验。一时不能检验的样品应存放在凉爽、通风良好的室内或冰箱中，使种子品质的变化降到最低限度。检验机构对保存的样品发生的劣变不承担责任。高含水量的种子难以妥善贮藏，应尽快检验。

3.5.2 为了便于复验，送检样品自发证之日起要放在适宜条件下保存四个月，使种子品质的变化降至最低限度。低含水量的种子样品放入密封的塑料袋中，在3～5℃下可以保存很长时间不会变化。供测定含水量和测定种子健康状况的送检样品，检验后不必保存。

4 净度分析

4.1 目的

测定供检验样品中纯净种子、其他植物种子和夹杂物的重量百分率，据此推断种批的组成。

4.2 定义

4.2.1 净度

测定样品中纯净种子重量占测定后样品各成分重量总和的百分数。

4.2.2 纯净种子

a）送检者陈述的种或分析中发现的主要种（包括该种的变种和栽培品种）的种子，是完整的、没有受伤害的、发育正常的种子；发育不完全的种子和不能识别出的空粒；虽已破口或发芽，但仍具发芽能力的种子。

b）带翅的种子中，凡加工时种翅容易脱落的，其纯净种子是指除去种翅的种子；凡加工时种翅不易脱落的，则不必除去，其纯净种子包括留在种子上的种翅。附录F表F1乔灌木种子示意图可以帮助检验人员作出判断。

c）壳斗科的纯净种子是否包括壳斗，取决于各个种的具体情况：壳斗容易脱落的不包括壳斗；难于脱落的包括壳斗。

d）复粒种子中至少含有一粒种子的。

4.2.3 其他植物种子

分类学上与纯净种子不同的其他植物种子。

4.2.4 夹杂物

a）能明显识别的空粒、腐坏粒、已萌芽因而显然丧失发芽能力的种子；

b）严重损伤（超过原大小一半）的种子和无种皮的裸粒种子；

c）叶片、鳞片、苞片、果皮、种翅、壳斗、种子碎片、土块和其他杂质；

d）昆虫的卵块、成虫、幼虫和蛹。

4.2.5 粘滞性种子

由于结构或质地上的特点这类种子可分为：

a）容易相互粘附或容易粘附在其他物体（如包装袋、分样器等）上；

b）容易被其他植物种子粘附，或容易粘附其他植物种子；

c）不易被清选、混合或扦样。

如果全部粘滞性结构（包括粘滞性杂质）占一个样品的三分之一或更多，就认为该样品是有粘滞性。例如冷杉属、翠柏属、雪松属、扁柏属、柏木属、柳杉属、杉木属、落叶松属、云杉属、长叶松、刚松、黄杉属、红杉属、巨杉属、落羽杉属、铁杉属、槭属、臭椿属、桤木属、桦木属、鹅耳枥属、梓属、石竹属、桉属、水青冈属、银桦属、女贞属、枫香属、鹅掌楸属、悬铃木属、竹类、杨属、香椿属、丁香属、崖柏属、榔树属、榆属、榉属等都是粘滞性种子，应用容许差距表2、表3时，应当使用粘滞性种子栏的容许误差。

见附录F表F1乔灌木种子示意图。

4.3 原则

将测定样品分成纯净种子、其他植物种子和夹杂物三个组成部分，并测定各部分的重量百分率。样品中所有植物种子和各种夹杂物，应尽可能加以鉴定。

样品中含有两个或两个以上的种难以区分时，允许只填报其属名，符合4.2.2定义的该属的全部种子均为纯净种子。

4.4 程序

4.4.1 测定样品

a）送检样品中混有较大的或多量的夹杂物时，要在样品称重后，分取测定样品前，进行必要的清理并称重。用经过初步清理后的送检样品分取测定样品进行净度测定。

b）净度分析用的测定样品的最低量见附录A表A1规定。除种粒大的至少为500粒外，其他树种通常要求至少含有纯净种子2 500粒。

c）测定样品可以是按附录A表A1规定重量的一个测定样品（一个全样品），或者至少是这个重量一半的两个各自独立分取的测定样品（两个“半样品”）。必要时也可以是两个全样品。

d）为使百分数可以计算到一位小数，样品的总体及其各个组成成分的称量精度要求见表1。

表1 净度分析样品的总体及各个组成成分的称量精度

测定样品重，g<br>（全样品或“半样品”）	称量至小数位数<br>（全样品或“半样品”及其组成）
1.000 0以下	4
1.000～9.999	3
10.00～99.99	2
100.0～999.9	1
1 000或1 000以上	0

e）用称量发芽法检验时，不必测定净度，遇有大的杂质，可按a)处理。

4.4.2 分离

测定样品称重后，按4.2将其中各种成分分离，分别按4.4.1d)要求的精确度称量，填入附录C表C2。

4.5 结果计算

4.5.1 全样品的原重减去净度分析后纯净种子、其他植物种子和夹杂物的重量和，其差值不得大于原重的5%，否则需重做。

4.5.2 用两个“半样品”时，每份“半样品”各自将所有成分的重量相加，如果同原重量的差距超过原重量5%，需再分析两个“半样品”。

4.5.3 分别计算两个“半样品”或两个全样品每个成分的重量占各成分重量之和的百分率（至少保留两位小数），并根据4.5.4、4.5.5和4.5.6的规定，检查两份全样品、两份“半样品”每个成分分析结果之间的差异是否超过容许差异。如果各个成分均在容许范围之内，可以计算并在质量检验证书中填报每个成分重量百分数的平均数。

任何一个成分的分析结果超过了容许差距，均按以下程序处理：

4.5.3.1 在使用“半样品”的情况下，再分析一对“半样品”（但总共不必多于四对），直至一对“半样品”各成分的差距均在容许范围之内。将其成分的差异超过容许差距两倍的成对样品舍去不计，根据其余各对的数据计算各个成分的百分数的平均值。

4.5.3.2 在使用两份全样品的情况下，再分析一份样品。只要最高值和最低值的差异未超过容许差距的两倍，就取这三次分析的平均值填报。除非其中有的结果显然是由于差错而不是随机样品误差引起的，在这种情况下，将错误的结果舍去不计。

4.5.4 表2用于同一实验室，对同一送检样品的净度分析结果重复间的比较，适用于任何成分。使用时先按两次分析结果的平均值从栏1或栏2中找到相应的行，根据种子是否为粘滞性种子和分析的是半

样品还是全样品，从栏 3 至栏 6 之一栏中找出其相应的容许差距。

4.5.5 表 3 用于来自同一种批的两个不同送检样品的净度分析，两次分析可能是在相同或不同实验室进行，且当第二次分析结果低于第一次分析结果时使用，适用于任何成分。使用时先按两次分析结果的平均值从栏 1 或栏 2 中找到相应的行，再根据种子是否为粘滞性种子从栏 3 或栏 4 中找出其相应的容许差距。

4.5.6 表 4 适用于来自同一种批的两个不同送检样品的净度分析，两次分析可以在相同或不同实验室进行，适用于净度分析中任何成分的比较，以确定两个估算值是否一致。使用时先按两次分析结果的平均值从栏 1 或栏 2 中找到相应的行，根据种子是否为粘滞性种子从栏 3 或栏 4 中找出其相应的容许差距。

表 2 同实验室同送检样品净度分析容许差距(5%显著水平的两尾测定)

两次分析结果平均		不同测定之间的容许差距			
		半样品		全样品	
50%～100%	<50%	非粘滞性种子	粘滞性种子	非粘滞性种子	粘滞性种子
1	2	3	4	5	6
99.95～100.00	0.00～0.04	0.20	0.23	0.1	0.2
99.90～99.94	0.05～0.09	0.33	0.34	0.2	0.2
99.85～99.89	0.10～0.14	0.40	0.42	0.3	0.3
99.80～99.84	0.15～0.19	0.47	0.49	0.3	0.4
99.75～99.79	0.20～0.24	0.51	0.55	0.4	0.4
99.70～99.74	0.25～0.29	0.55	0.59	0.4	0.4
99.65～99.69	0.30～0.34	0.61	0.65	0.4	0.5
99.60～99.64	0.35～0.39	0.65	0.69	0.5	0.5
99.55～99.59	0.40～0.44	0.68	0.74	0.5	0.5
99.50～99.54	0.45～0.49	0.72	0.76	0.5	0.5
99.40～99.49	0.50～0.59	0.76	0.82	0.5	0.6
99.30～99.39	0.60～0.69	0.83	0.89	0.6	0.6
99.20～99.29	0.70～0.79	0.89	0.95	0.6	0.7
99.10～99.19	0.80～0.89	0.95	1.00	0.7	0.7
99.00～99.09	0.90～0.99	1.00	1.06	0.7	0.8
98.75～98.99	1.00～1.24	1.07	1.15	0.8	0.8
98.50～98.74	1.25～1.49	1.19	1.26	0.8	0.9
98.25～98.49	1.50～1.74	1.29	1.37	0.9	1.0
98.00～98.24	1.75～1.99	1.37	1.47	1.0	1.0
97.75～97.99	2.00～2.24	1.44	1.54	1.0	1.1
97.50～97.74	2.25～2.49	1.53	1.63	1.1	1.2
97.25～97.49	2.50～2.74	1.60	1.70	1.1	1.2
97.00～97.24	2.75～2.99	1.67	1.78	1.2	1.3
96.50～96.99	3.00～3.49	1.77	1.88	1.3	1.3
96.00～96.49	3.50～3.99	1.88	1.99	1.3	1.4
95.50～95.99	4.00～4.49	1.99	2.12	1.4	1.5
95.00～95.49	4.50～4.99	2.09	2.22	1.5	1.6
94.00～94.99	5.00～5.99	2.25	2.38	1.6	1.7
93.00～93.99	6.00～6.99	2.43	2.56	1.7	1.8
92.00～92.99	7.00～7.99	2.59	2.73	1.8	1.9
91.00～91.99	8.00～8.99	2.74	2.90	1.9	2.1
90.00～90.99	9.00～9.99	2.88	3.04	2.0	2.2

表 2（完）

两次分析结果平均		不同测定之间的容许差距			
		半　样　品		全　样　品	
50%～100%	<50%	非粘滞性种子	粘滞性种子	非粘滞性种子	粘滞性种子
1	2	3	4	5	6
88.00～89.99	10.00～11.99	3.08	3.25	2.2	2.3
86.00～87.99	12.00～13.99	3.31	3.49	2.3	2.5
84.00～85.99	14.00～15.99	3.52	3.71	2.5	2.6
82.00～83.99	16.00～17.99	3.69	3.90	2.6	2.8
80.00～81.99	18.00～19.99	3.86	4.07	2.7	2.9
78.00～79.99	20.00～21.99	4.00	4.23	2.8	3.0
76.00～77.99	22.00～23.99	4.14	4.37	2.9	3.1
74.00～75.99	24.00～25.99	4.26	4.50	3.0	3.2
72.00～73.99	26.00～27.99	4.37	4.61	3.1	3.3
70.00～71.99	28.00～29.99	4.47	4.71	3.2	3.3
65.00～69.99	30.00～34.99	4.61	4.86	3.3	3.4
60.00～64.99	35.00～39.99	4.77	5.02	3.4	3.6
50.00～59.99	40.00～49.99	4.89	5.16	3.5	3.7

表 3　相同或不同实验室不同送检样品净度分析容许差距

（用于两份全样品，1%显著水平的一尾测定）

两次结果平均		容许差距	
50%～100%	<50%	非粘滞性种子	粘滞性种子
1	2	3	4
99.95～100.00	0.00～0.04	0.2	0.2
99.90～99.99	0.05～0.09	0.3	0.3
99.85～99.89	0.10～0.14	0.3	0.4
99.80～99.84	0.15～0.19	0.4	0.5
99.75～99.79	0.20～0.24	0.4	0.5
99.70～99.74	0.25～0.29	0.5	0.6
99.65～99.69	0.30～0.34	0.5	0.6
99.60～99.64	0.35～0.39	0.6	0.7
99.55～99.59	0.40～0.44	0.6	0.7
99.50～99.54	0.45～0.49	0.6	0.7
99.40～99.49	0.50～0.59	0.7	0.8
99.30～99.39	0.60～0.69	0.7	0.9
99.20～99.29	0.70～0.79	0.8	0.9
99.10～99.19	0.80～0.89	0.8	1.0
99.00～99.09	0.90～0.99	0.9	1.0
98.75～98.99	1.00～1.24	0.9	1.1
98.50～98.74	1.25～1.49	1.0	1.2
98.25～98.49	1.50～1.74	1.1	1.3
98.00～98.24	1.75～1.99	1.2	1.4
97.75～97.99	2.00～2.24	1.3	1.5
97.50～97.74	2.25～2.49	1.3	1.6
97.25～97.49	2.50～2.74	1.4	1.6
97.00～97.24	2.75～2.99	1.5	1.7

表 3（完）

两次结果平均		容许差距	
50％～100％	＜50％	非粘滞性种子	粘滞性种子
1	2	3	4
96.50～96.99	3.00～3.49	1.5	1.8
96.00～96.49	3.50～3.99	1.6	1.9
95.50～95.99	4.00～4.49	1.7	2.0
95.00～95.49	4.50～4.99	1.8	2.2
94.00～94.99	5.00～5.99	2.0	2.3
93.00～93.99	6.00～6.99	2.1	2.5
92.00～92.99	7.00～7.99	2.2	2.6
91.00～91.99	8.00～8.99	2.4	2.8
90.00～90.99	9.00～9.99	2.5	2.9
88.00～89.99	10.00～11.99	2.7	3.1
86.00～87.99	12.00～13.99	2.9	3.4
84.00～85.99	14.00～15.99	3.0	3.6
82.00～83.99	16.00～17.99	3.2	3.7
80.00～81.99	18.00～19.99	3.3	3.9
78.00～79.99	20.00～21.99	3.5	4.1
76.00～77.99	22.00～23.99	3.6	4.2
74.00～75.99	24.00～25.99	3.7	4.3
72.00～73.99	26.00～27.99	3.8	4.4
70.00～71.99	28.00～29.99	3.8	4.5
65.00～69.99	30.00～34.99	4.0	4.7
60.00～64.99	35.00～39.99	4.1	4.8
50.00～59.99	40.00～49.99	4.2	5.0

表 4　相同或不同实验室不同送检样品净度分析容许差距

（用于两份全样品，1％显著水平的两尾测定）

两次结果平均		容许差距	
50％～100％	＜50％	非粘滞性种子	粘滞性种子
1	2	3	4
99.95～100.00	0.00～0.04	0.18	0.21
99.90～99.99	0.05～0.09	0.28	0.32
99.85～99.89	0.10～0.14	0.34	0.40
99.80～99.84	0.15～0.19	0.40	0.47
99.75～99.79	0.20～0.24	0.44	0.53
99.70～99.74	0.25～0.29	0.49	0.57
99.65～99.69	0.30～0.34	0.53	0.62
99.60～99.64	0.35～0.39	0.57	0.66
99.55～99.59	0.40～0.44	0.60	0.70
99.50～99.54	0.45～0.49	0.63	0.73
99.40～99.49	0.50～0.59	0.68	0.79
99.30～99.39	0.60～0.69	0.73	0.85
99.20～99.29	0.70～0.79	0.78	0.91
99.10～99.19	0.80～0.89	0.83	0.96
99.00～99.09	0.90～0.99	0.87	1.01

表 4（完）

两次结果平均		容许差距	
50%～100%	<50%	非粘滞性种子	粘滞性种子
1	2	3	4
98.75～98.99	1.00～1.24	0.94	1.10
98.50～98.74	1.25～1.49	1.04	1.21
98.25～98.49	1.50～1.74	1.12	1.31
98.00～98.24	1.75～1.99	1.20	1.40
97.75～97.99	2.00～2.24	1.26	1.47
97.50～97.74	2.25～2.49	1.33	1.55
97.25～97.49	2.50～2.74	1.39	1.63
97.00～97.24	2.75～2.99	1.46	1.70
96.50～96.99	3.00～3.49	1.54	1.80
96.00～96.49	3.50～3.99	1.64	1.92
95.50～95.99	4.00～4.49	1.74	2.04
95.00～95.49	4.50～4.99	1.83	2.15
94.00～94.99	5.00～5.99	1.95	2.29
93.00～93.99	6.00～6.99	2.10	2.46
92.00～92.99	7.00～7.99	2.23	2.62
91.00～91.99	8.00～8.99	2.36	2.76
90.00～90.99	9.00～9.99	2.48	2.92
88.00～89.99	10.00～11.99	2.65	3.11
86.00～87.99	12.00～13.99	2.85	3.35
84.00～85.99	14.00～15.99	3.02	3.55
82.00～83.99	16.00～17.99	3.18	3.74
80.00～81.99	18.00～19.99	3.32	3.90
78.00～79.99	20.00～21.99	3.45	4.05
76.00～77.99	22.00～23.99	3.56	4.19
74.00～75.99	24.00～25.99	3.67	4.31
72.00～73.99	26.00～27.99	3.76	4.42
70.00～71.99	28.00～29.99	3.84	4.51
65.00～69.99	30.00～34.99	3.97	4.66
60.00～64.99	35.00～39.99	4.10	4.82
50.00～59.99	40.00～49.99	4.21	4.95

4.5.7 计算方法

4.5.7.1 测定样品的净度用式(1)计算：

$$净度(\%)=\frac{纯净种子重}{纯净种子重+其他植物种子重+夹杂物重}\times 100 \quad \cdots\cdots(1)$$

4.5.7.2 送检样品先行清理的净度用式(2)、式(3)计算：

$$送检样品净度(\%)=\frac{送检样品除去大型杂质后的重量}{送检样品重}\times 100 \quad \cdots\cdots(2)$$

$$净度(\%)=送检样品净度\times测定样品净度 \quad \cdots\cdots(3)$$

4.5.7.3 其他植物种子的重量百分数和夹杂物的重量百分数的计算方法与纯净种子重量百分数(即净度)的计算方法相同。

4.6 结果报告

净度分析中各个成分应计算到两位小数，在质量检验证书上填写时按 GB/T 8170 修约到一位小数。成分少于 0.05%的填报为“微量”，若成分为零时用“—0.0—”表示。测定样品各成分总和必须为

100%。总和是 99.9%和 100.1%时,可从百分率的最大值(通常是纯净种子部分)中加减 0.1%,如修约值超过 0.1%,应核查计算有无差错。

5 发芽测定

5.1 目的

在室内标准条件下测定种批的最大发芽潜力,使测定结果能在最接近于随机样本变异的范围内重现,据此比较不同种批的品质并估计田间播种价值。

5.2 定义

5.2.1 发芽

室内测定一粒种子发芽,是指幼苗出现并生长到某个阶段,其基本结构的状况表明它是否能在正常的田间条件下进一步长成一株合格苗木。

5.2.2 发芽率

质量检验证书上填报的发芽率,是在附录 B 表 B1 规定的条件下及其规定的期限内生成正常幼苗的种子粒数占供检种子总数的百分比。

5.2.3 幼苗基本结构

对于幼苗继续生长成为合格苗木必不可少的结构。随所检树种而不同,组成幼苗的是以下某些基本结构的特定组合:根系、胚轴、子叶、初生叶、顶芽以及禾本科、棕榈科的芽鞘。

5.2.4 正常幼苗

表现出具有潜力,能在土质良好,水分、温度、光照适宜的条件下继续生长成为合格苗木的幼苗。符合下列类型之一的可以划为正常幼苗:

a) 完整幼苗:该树种应有的基本结构全都完整、匀称、健康、生长良好;

b) 带轻微缺陷的幼苗:该树种应有的基本结构出现某些轻微缺陷的幼苗,但其他方面正常,生长均衡,与同次测定中完整幼苗的其他方面不相上下;

c) 受到次生性感染的幼苗:显然本该属于上述 a)类或 b)类但受真菌或细菌感染的幼苗,条件是该粒种子不是感染源。

5.2.5 不正常幼苗

表现出没有潜力,在土质良好,水分、温度、光照适宜的条件下不能长成合格苗木的幼苗。不正常幼苗有三种类型:

a) 损伤苗:任何基本结构缺失,或损伤严重无法恢复正常,不能指望均衡生长的幼苗;

b) 畸形苗或不匀称苗:生长孱弱或生理紊乱,或基本结构畸形或失衡的幼苗;

c) 腐坏苗:由于原发性感染(即该粒种子就是感染源),该树种的任何基本结构染病或腐坏,停止正常生长的幼苗。

例如,具有下列情况之一的幼苗为不正常幼苗:

a) 初生根:生长停滞、粗短、缺失、断折、自顶端开裂、缢缩、纤细、束缚在种皮中、呈负向地性、玻璃状、因原发性感染而腐坏、禾木科植物种子无种子根或仅有一条孱弱的种子根;

b) 下胚轴、上胚轴和中胚轴:粗短、深度横裂或断折、完全纵裂、缺失、缢缩、极度扭曲、弯曲向下、呈环状或螺旋状、纤细、玻璃状、因原发性感染而腐坏;

c) 子叶(下述缺陷所占面积超过子叶面积的一半者为不正常,只占一半或不足一半者为正常,称为“50%规则”。但只要损伤或腐坏出现在子叶同幼苗中轴的联结点上或者茎尖附近,该幼苗就属于不正常幼苗,在这种情况下不考虑 50%规则。):肿胀或卷曲、畸形、断折或有其他损伤、断离或缺失、变色、坏死、玻璃状、因原发性感染而腐坏;

d) 初生叶:畸形、损伤、缺失、变色、坏死、外形正常但小于正常大小的四分之一、因原发性感染而腐坏;

e) 顶芽及其周围的组织:畸形、损伤、缺失、因原发性感染而腐坏(如果顶芽有缺陷或者缺失,即使

已经生出一个或两个侧芽，该幼苗仍为不正常幼苗)；

f) 芽鞘和第一片叶(禾本科、棕榈科)

芽鞘：畸形、损伤、缺失、顶端损伤或缺失、极度向下弯曲、呈环状或螺旋状、严重扭曲，从顶端向下开裂长度超过全长的三分之一，基部开裂或有其他损伤。

第一片叶：伸展长度不及芽鞘的一半、缺失、撕裂或呈其他畸形；

g) 幼苗整体：畸形、断裂、子叶先出、二苗融合、胚乳环圈不落、黄化或白化、纤细、玻璃状、因原发性感染而腐坏。

5.2.6 多苗种子单位

能够产生多株幼苗的种子单位。有以下几种类型：

a) 种子单位内含的真种子多于一粒，例如柚木(*Tectona grandis*)的坚果和楝树(*Melia azedarach*)的核果；

b) 真种子内含的胚多于一枚。有些种这是正常现象(多胚现象)，有些种则是偶尔出现(孪生现象)。如果是孪生胚，通常是其中一株幼苗弱小纤细，但偶尔也会是两株幼苗都接近正常大小；

c) 融合胚：偶尔会从一粒种子中生出两株融合在一起的幼苗。

5.2.7 未萌发粒

在附录B表B1给定的测定条件下测定期结束时仍未发芽的种子。可以区分成几个类型：

a) 硬粒：在测定条件下未能吸水而在测定期末仍然坚硬的种子。一种休眠形态，常见于豆科，也能出现于其他某些科；

b) 新鲜粒：在测定条件下能够吸水但发芽进程受阻，外形依旧良好，坚实硬朗，仍然具有生出正常幼苗潜力的种子；

c) 死亡粒：测定期末既非硬粒，又非新鲜粒，又未萌出幼苗任何结构的种子，通常包被物极软、变色、发霉且毫无生出幼苗的征兆；

d) 其他类型：根据送检单位的要求，未萌发粒还可细分为以下几类，它们在测定样品中所占的百分数可填入质量检验证书的备注栏：

空粒——完全空瘪或仅含某种残遗组织的种子；

涩粒——杉木、柳杉胚珠受精后败育，内含物为紫黑色单宁类物质的种子；

无胚粒——种子内有新鲜胚乳或配子体组织，但其中既无胚腔，也没有胚；

虫害粒——内有昆虫的幼虫或虫粪或有其他迹象表明受到过昆虫侵害，影响发芽能力的种子。

5.3 发芽床

5.3.1 纸床

用作发芽床的纸应是疏松、通气、无毒、无菌、容易向种子不断提供水分的滤纸或其他类型的纸，或者是洁净无毒的纱布、脱脂棉。种子排放在床面。为了向种子提供足够的水分，可以用多层的纸，也可以在纸下加垫纱布或脱脂棉。作床材料的pH值应在6.0～7.5范围内。

发芽测定用纸应在干燥凉爽的室内存放并适当包装，避免污损破伤，必要时使用前应当灭菌，消除存放期中可能感染的霉菌。

5.3.2 沙床

作床的沙应当颗粒均匀一致，能通过直径为0.8 mm的筛孔，而截留在孔眼直径为0.05 mm的筛子上。沙必须无菌无毒，不含任何种子。沙的pH值应在6.0～7.5范围内。沙不能重复使用。

5.3.3 土床

只有在纸床、沙床上发芽的幼苗出现植物毒性症状，或者对纸床上的幼苗的鉴定发生怀疑时，才可以使用质地疏松、结构良好、不会板结的壤土作床，使用时不能挤压。质地紧密的土壤应适当混加蛭石或沙。土中不能混有任何种子。土的pH值应在6.0～7.5之间。土不能重复使用。

5.4 发芽设备

5.4.1 发芽盒

用于纸床的带盖的、内具有孔隔板的透明发芽容器。盒的长度和宽度应能容纳四次或至少两次重复的种粒。盒高应略大于受检树种正常幼苗的高度。发芽床铺放在具有孔眼的隔板上。隔板同盒底之间的空间用于存水，使盒内的相对湿度尽可能接近 100%。

5.4.2 直立板发芽盒

有机玻璃制成的长方形盒，盒高约 20 cm。盒内可以垂直嵌入若干块中心距约 2 cm 的有机玻板。在玻板顶边之下适当距离处播放一排种子并覆以同玻板等大的湿滤纸。滤纸下端浸入盒底的水中向种子供水。改变滤纸下端入水的深度可以控制供水量。盖上盒盖可以保持盒内空气湿度。从玻板的反面可以清晰地观察种子的发芽状况。

5.4.3 带有水箱的发芽装置

主体是镀锌钢板或不锈钢板制作的水箱，水箱顶板上排放钟形发芽皿。钟形发芽皿的芯带穿过顶板上的孔眼或窄缝伸入水箱吸水。水温由控温器控制，为发芽环境提供所需的温度。水箱上方悬放冷白荧光灯管。

5.4.4 发芽箱

能调控温度、湿度和光照的密闭箱体。好的发芽箱应当具有加热和降温两个系统，隔热性能良好，能提供发芽所需的光照条件，且能控制湿度，能使箱内的相对湿度接近 100%。箱内的隔板承放纸床，直接排放供检样品。隔板的间距应使同一时间里能够测定尽可能多的样品。不能控制湿度的温箱应当使用 5.4.1 描述的发芽盒或 5.4.2 描述的直立板发芽盒盛放供检样品。

5.4.5 发芽室

能调控温度并有光照设备、专供发芽测定使用的房间。整个发芽室的湿度较难控制，一般用发芽盒或直立板发芽盒向种子提供水分并保持发芽小环境的湿度。

5.5 测定程序

5.5.1 提取测定样品

测定样品从净度分析所得的、经过充分混拌的纯净种子中按照随机原则提取。可以用四分法将纯净种子区分成 4 份，从每份中随机数取 25 粒组成 100 粒，共取 4 个 100 粒，即为 4 次重复。也可以用数粒器提取 4 次重复。种粒大的，或者怀疑种子带有病菌的，可以将 100 粒的每个重复以 50 粒或 25 粒为一组，以组为单位在发芽床上排放，由这样的 2 个组或 4 个组组成 1 次重复，使种粒之间有足够的距离。

无论是人工数取还是用数粒器提取样品，都必须避免有意识或无意识地对种子作任何选择。

附录 B 表 B1 规定采用称量发芽测定法的树种，或因种粒特小决定采用称量发芽测定法的其他树种，用符合第 3 章规定的程序从送检样品中提取测定样品，共取 4 个重复。每个重复的重量见附录 B 表 B1。

5.5.2 测定样品的预处理

对测定样品作预处理的目的是解除休眠。预处理的方法已分树种列在附录 B 表 B1 的备注栏。也可以采用其他有效的预处理方法，但必须在质量检验证书中注明。如果不能肯定某种预处理方法是否有效，可以在 5.5.1 规定的 4 个重复之外，再取 4 个重复作另一份测定样品，或再取 8 个重复作为另外两份测定样品，用不同的方法作预处理，同时作发芽测定，以其中最好的结果作为该次测定的结果填报，并注明所用的预处理方法。

附录 B 表 B1 所列的测定时间不包括预处理时间。带翅的种子可以去翅，但不能伤及种子。

5.5.3 置床和管理

5.5.3.1 种粒在发芽床上应保持一定距离，避免病菌蔓延、根系缠绕，也便于点数。

5.5.3.2 采用沙床(土床)时，需光种子压入沙(土)的表层，忌光种子播在疏松而平整的沙(土)之上，再均匀疏松地加盖厚度为 10～20 mm 的沙(土)。

5.5.3.3 发芽容器和直立板发芽盒中的直立板应当编号。特别是要由几个容器或几块直立板组成一次重复的，必须在置床时通过编号确定由哪几个容器或哪几块板组成哪一次重复，绝不允许为了通过误差

检验而任意组合。

5.5.3.4 经常检查测定样品及其水分、通气、温度、光照条件。检查的间隔时间由检验机构根据树种特性和样品状况等自行确定。轻微发霉的种粒可以拣出用清水冲洗后放回原发芽床。发霉种粒较多的要及时更换发芽床或发芽容器。

5.5.4 测定条件

5.5.4.1 水

发芽床的用水不应含有杂质。水的pH值应在6.0～7.5之间。如果当地的水质不符合要求,可以使用蒸馏水或去离子水。

发芽床应始终保持湿润,不断地向种子提供所需的水分。但供水过量也会影响种子的通气。对种子的供水量取决于受检树种的特性、发芽床的性质以及发芽盒的种类(5.4.1、5.4.2和5.4.3),由检验机构根据经验确定。各重复间的供水量应当一致。

5.5.4.2 通气

置床的种子要保持通气良好,但不能使发芽床过度失水而影响萌发。

5.5.4.3 温度

附录B表B1所列的温度是指发芽床上种子所处水平层次的温度,因设备性能而产生的温度变化不能超过±1℃。为发芽种子提供光照时不能使温度发生波动。

附录B表B1列出带有幅度的温度指的是变温,每24 h内应有16 h保持较低的那个温度,其余8 h保持较高的那个温度。温度的转换最好在3 h内逐渐完成,休眠性种子可以在1 h内完成。周末或节假日不能按要求转换温度时,应使发芽环境保持在较低的那个温度水平上。

附录B表B1中有的树种列有几种温度,它们的排列顺序并不表示其优劣,可以根据检验工作的方便选用。质量检验证书中应当注明实际采用的温度。

5.5.4.4 光

除非确已证实某个树种的发芽会受到光抑制,否则发芽测定中的每个24 h都应当给予8 h的光照,使幼苗长势良好,不容易遭受微生物侵害,也便于评定。施加的光指的是不含或极少含远红光的冷白色荧光。提供的光应均匀一致地使种子表面接受750～1 250 lx的照度。对于变温发芽的树种,是在给予高温的那个8 h内提供光照。

5.5.5 测定的持续时间

5.5.5.1 各树种发芽测定的持续时间在附录B表B1中以末次记数的天数表示,自置床之日起算,不包括预处理的时间。

5.5.5.2 如果测定样品在规定时间里发芽的种粒不多,可以适当延长测定时间。延长的时间最多不应超过规定时间的二分之一。如果在规定的结束时间之前样品已经充分发芽,且后期连续三天每天的发芽粒数不超过各重复供试种子粒数的1%,则该次测定可以提前结束。无论是延长还是提前结束,测定的实际天数应在质量检验证书中注明。

附录B表B1中有些树种列有几种可供选择的温度。如果选用的是较低的温度,初次计数可以适当推迟。如果使用的是沙床且测定持续时间不足10天,也可以不作初次计数。初次计数是使检验人员对供检样品的质量状况可以较早地作出估计,所记数值并不要求填报。

5.5.5.3 中期计数的次数由检验机构自行确定,除有特殊要求外,通常不宜过多,尽量减少对尚未充分生长的幼苗造成伤害。

5.5.6 观察、评定和记载

5.5.6.1 发芽测定的情况要定期观察记载。观察记载的间隔时间由检验机构根据树种和样品情况自行确定,但初次计数(除5.5.3.2所述的沙床以外)和末次计数必须有记载。

5.5.6.2 生长到一定阶段,必要的基本结构都已展现的每株幼苗都必须根据5.2.4和5.2.5进行评定,并在记数后从发芽床上拣出。严重腐坏的幼苗也应拣出,以免造成次生性感染。呈现其他缺陷的不正常幼苗则应保留在发芽床上直至末次计数。拣出的幼苗数同发芽床上剩余的种粒数应当等于前一次

记载时的剩余种粒数。拣出幼苗时要防止影响正在发芽生长的幼苗。

5.5.6.3　一个多苗种子单位常能生出两株或几株幼苗，无论其中有几株符合5.2.4的定义，都记为一株正常幼苗。根据要求，也可以统计并填报100个多苗种子单位生出的正常幼苗总数，或者分别统计并填报生出了一株、两株或更多正常幼苗的多苗种子数。

5.5.6.4　测定结束时根据5.2.7给出的定义区分未发芽粒。新鲜粒的鉴定可以采用四唑染色法、切开法、离体胚发芽法或X射线衬比法。无法判定是新鲜粒还是死亡粒的一律记为死亡粒。已经生出了幼苗的某些部位（例如根尖），即使在评定时确已腐坏，也不记为死亡种子而记为不正常幼苗。根据要求需要对属于5.2.7d）的未发芽粒分类记数时，可以采用以下方法：

a）发芽测定之前：

——测定样品的各个重复先作X射线检验；

——另取四份重复用切开法检验。

b）发芽测定之后：

——切开法；

——四唑染色法或靛蓝染色法。

5.6　测定结果的计算

5.6.1　发芽测定结束时按5.2.2计算发芽率，同时按5.2.5计算不正常幼苗以及按5.2.7中a)、b)、c)计算硬粒、新鲜粒和死亡粒的百分数。根据要求，还可以计算空粒、涩粒、无胚粒和虫害粒(5.2.7.d))的百分数。

5.6.2　5.6.1所列的各种百分数乃是100粒种子4个重复的平均数。以50粒为一组的，应在计算前按原编号(见5.5.3.3)组合成4个重复。计算结果按GB/T 8170修约至整数。正常幼苗百分数、不正常幼苗百分数和未发芽粒百分数之和必须等于100。

5.6.3　按表5.1检查重复间发芽百分数的差异是否为随机误差。如果各重复发芽百分数的最大值同最小值的差距没有超过表5的容许范围，就用各重复发芽百分率的平均数作为该次测定的发芽率。

表5　发芽测定容许差距

平均发芽百分率		最大容许差距
1	2	3
99	2	5
98	3	6
97	4	7
96	5	8
95	6	9
93～94	7～8	10
91～92	9～10	11
89～90	11～12	12
87～88	13～14	13
84～86	15～17	14
81～83	18～20	15
78～80	21～23	16
73～77	24～28	17
67～72	29～34	18
56～66	35～45	19
51～55	46～50	20

5.6.4　称量发芽测定法的测定结果用单位重量样品中的正常幼苗数表示，单位为株/克。计算时，利用表6检查重复间的差异是否为随机误差。先计算4个重复正常幼苗的总数，在表6第1栏找出该总数所在范围，并在第2栏中查到最大容许差距。如果4个重复中正常幼苗数的最大值和最小值之差等于或小于最大容许差距，该次测定可靠，以4个重复单位重量的正常幼苗数的平均数作为测定结果填报。

表 6 称量发芽测定容许差距

供检样品总重量中的正常发芽粒数	最大容许差距	供检样品总重量中的正常发芽粒数	最大容许差距
1	2	1	2
0～6	4	161～174	27
7～10	6	175～188	28
11～14	8	189～202	29
15～18	9	203～216	30
19～22	11	217～230	31
23～26	12	231～244	32
27～30	13	245～256	33
31～38	14	257～270	34
39～50	15	271～288	35
51～56	16	289～302	36
57～62	17	303～321	37
63～70	18	322～338	38
71～82	19	339～358	39
83～90	20	359～378	40
91～102	21	379～402	41
103～112	22	403～420	42
113～122	23	421～438	43
123～134	24	439～460	44
135～146	25	＞460	45
147～160	26		

5.7 重新测定

有下列情况之一时应重新布置测定。重新测定仍按 5.6 的规定计算结果并检查误差。

a）怀疑是休眠干扰测定结果时，可以仍按附录 B 表 B1 的发芽条件，但选用一种或几种解除休眠的方法重新布置一次或同时布置几次测定，将其中最好的结果作为测定结果填报，并注明所用方法。

b）由于病毒或真菌、细菌蔓延干扰测定结果时，可以使用沙床或土床重新布置一次或同时布置几次测定。必要时还可加大种粒间的距离。将所得的最好结果作为测定结果填报，并注明所用方法。

c）难于评定的幼苗数量较多而干扰测定结果时，可以仍按附录 B 表 B1 的发芽条件，用沙床或土床重新布置一次或同时布置几次测定。将所得的最好结果作为测定结果填报，并注明所用方法。

d）由于测定条件、幼苗评定或计数显然有误时，应当按原用方法重新测定，并填报重新测定的结果。

e）由于其他不明因素使得各重复间的最大差距超过表 5 规定的容许误差时，应当提取测定样品用原定方法重新测定。如果第一次和第二次的测定结果一致，即两次测定结果之差不超过表 7 规定的最大容许差距，则以两次测定的平均数作为测定结果填报。如果两次测定结果不一致，即它们的差异超过表 7 规定的最大容许差距，应当仍用同样的测定方法布置第三次测定。以三次测定中相互一致的两次的平均数作为测定结果填报。

表 7 重新发芽测定容许差距

两次测定的发芽平均数		最大容许误差	两次测定的发芽平均数		最大容许误差
1	2	3	1	2	3
98～99	2～3	2	77～84	17～24	6
95～97	4～6	3	60～76	25～41	7
91～94	7～10	4	51～59	42～50	8
85～90	11～16	5			

6 生活力测定

6.1 目的

种子生活力测定的目的是：

a）快速估测种子样品的生活力，特别是休眠种子样品的生活力；

b）某些样品在发芽测定结束时剩有较多的休眠种子未能萌发，此时可以逐粒测定这些种子的生活力，也可以再取一份样品测定样品的生活力。

6.2 定义

种子生活力是用染色法测得的种子潜在的发芽能力。

6.3 应用范围

本测定适用于附录 B 表 B2 给出方法的树种。

6.4 原理

6.4.1 四唑测定原理

应用 2,3,5-三苯基氯化（或溴化）四唑（2,3,5-triphenyl tetrazolium chloride or bromide）的无色溶液作为指示剂，以显示活细胞中所发生的还原过程。这种指示剂被种子吸收，在种子组织内与活细胞的还原过程起反应，从脱氢酶接受氢。在活细胞中，2,3,5-三苯基氯化四唑经氢化作用，生成一种红色而稳定的不扩散物质，即三苯基甲腊（triphenyl formazan）。这样就能识别出种子中红色的有生命部分和不染色的死亡部分。除完全染色的有生活力种子和完全不染色的无生活力种子外，还会出现一些部分染色的种子。在这些部分染色种子的不同部位能看到其中存在着或大或小的坏死组织，它们在胚和（或）胚乳（配子体）组织中所处的部位和大小（不一定是颜色的深浅），决定着这些种子是有生活力还是无生活力。不过同组织的健全程度相关联的颜色差异仍然被认为具有决定性意义，主要是因为在某种程度上，它们有助于识别出健全、衰弱或死亡组织并确定其位置。

6.4.2 靛蓝测定原理

靛蓝（indigo carmine）为蓝色粉末，分子式为 $C_{16}H_8N_2O_2(SO_3)_2Na_2$。靛蓝能透过死细胞组织使其染上颜色。因此，染上颜色的种子是无生活力的。根据胚染色的部位和比例大小来判断种子有无生活力。

6.5 试剂

6.5.1 使用氯化（或溴化）四唑的水溶液，浓度随树种而略有不同，见表 B2 中的规定。如果所使用蒸馏水的 pH 值不在 6.5～7.5 范围之内，可将四唑溶于缓冲溶液。缓冲溶液的配制方法如下：

溶液 a——在 1 000 mL 水中溶解 9.078 g 磷酸二氢钾（KH_2PO_4）；

溶液 b——在 1 000 mL 水中溶解 11.876 g 磷酸氢二钠（$Na_2HPO_4 \cdot 2H_2O$），或 9.472 g 磷酸氢二钠（Na_2HPO_4）。

取溶液 a 2 份和溶液 b 3 份混合，配成缓冲溶液。

在该缓冲溶液里溶解准确数量的四唑盐，以获得正确的浓度。例如，每 100 mL 缓冲溶液中溶入 1 g 四唑盐即得 1%浓度的溶液。最好随配随用。剩余的溶液可在短期内贮于低温 1～5℃的黑暗条件下。

6.5.2 靛蓝用蒸馏水配成浓度为 0.05%～0.1%的溶液，如发现溶液有沉淀，可适当加量，最好随配随用，不宜存放过久。

6.6 测定样品

从净度测定后的纯净种子中随机数取 100 粒种子作为一个重复，共取 4 个重复。或单用发芽测定结束的未萌发粒（见 5.2.7）。

6.7 种子预处理

6.7.1 去除种皮

为了软化种皮，便于剥取种仁，要对种子进行预处理。较易剥掉种皮的种子，可用始温 30～45℃的水浸种 24～48 h，每日换水，如杉木、马尾松、湿地松、火炬松、黄山松、米老排、安息香、黄连木、杜仲等。

硬粒的种子如肯氏相思、楹树、南洋楹、银合欢等可用始温 80～85℃水浸种，搅拌并在自然冷却中浸种 24～72 h，每日换水。种皮致密坚硬的种子，如孔雀豆、台湾相思、黑荆树、黑格、白格、漆树和滑桃树等，可用 98%的浓硫酸浸种 20～180 min，充分冲洗，再用水浸种 24～48 h，每日换水。

6.7.2 刺伤种皮

豆科的许多树种，如刺槐属，种子具有不透性种皮，可在胚根附近刺伤种皮或削去部分种皮，但不要伤胚。

6.7.3 切除部分种子

a）横切

为使四唑溶液均匀浸透，如女贞属，可以在浸种后在胚根相反的较宽一端将种子切去三分之一。

b）纵切

许多树种，如松属和白蜡属的种子可以纵切后染色。即在浸种后，平行于胚的纵轴纵向剖切，但不能穿过胚。白蜡属的种子可以在两边各切一刀，但不要伤胚。

c）取"胚方"

大粒种子如板栗、锥栗、核桃、银杏等可取"胚方"染色。取"胚方"是指经过浸种的种子，切取大约 1 cm^2包括胚根、胚轴和部分子叶（或胚乳）的方块。

6.8 方法

6.8.1 四唑染色法

6.8.1.1 程序

胚和胚乳均需进行染色鉴定。预处理时发现的空粒、腐烂粒和病虫害粒，记入附录C表C4中，属无生活力种子。剥种仁要细心，勿使胚损伤。剥出的种仁先放入盛有清水或垫有湿纱布或湿滤纸的器皿中，待全部剥完后再一起放入四唑溶液中，使溶液淹没种仁，上浮者要压沉。置黑暗处，保持 30～35℃，染色时间因树种和条件而异（见附录B表B2）。染色结束后，沥去溶液，用清水冲洗，将种仁摆在铺有湿滤纸的发芽皿中，保持湿润，以备鉴定。

6.8.1.2 鉴定

a）原则

根据染色的部位、染色面积的大小和同组织健壮程度有关的染色程度，逐粒判断种子的生活力。通过鉴定，将种子评为有生活力和无生活力两类，见附录B表B3。

b）染色后种子的处理

有些树种的种子染色之后，鉴定之前需要进一步处理，例如切开营养组织，或者切去一层营养组织，使胚的主要构造和活的营养组织明显暴露出来，以便观察。

6.8.2 靛蓝染色法

6.8.2.1 程序

胚和胚乳最好一起进行染色鉴定，剥取时要小心，勿使损伤。预处理时发现的空粒、腐烂粒和病虫害粒记入附录C表C4中。剥出的种仁先放入盛有清水或垫有湿沙布的器皿中。全部剥完后再放入靛蓝溶液，使溶液淹没胚，上浮者要压沉。染色时间因树种、温度而异（见附录B表B2，表B2规定的温度在 30～35℃）。

6.8.2.2 鉴定

a）原则

根据染色部位和比例大小来判断种子生活力。通过鉴定，将测定种子评为有生活力和无生活力两类，见附录B表B4。

b）染色后种子的处理见 6.8.1.2b）。

6.9 结果计算和表示

测定结果以有生活力种子的百分率表示，分别计算各个重复的百分率，重复间最大容许差距与发芽

测定相同(见表5)。如果各重复中最大值与最小值没有超过容许误差范围,就用各重复的平均数作为该次测定的生活力。如果各个重复间的最大差距超过表5规定的容许误差,与发芽测定同样处理(见5.7e))。计算结果按GB/T 8170修约至整数,在质量检验证书上填报。

7 优良度测定

7.1 目的

为在收购种子时根据种子外观和内部状况尽快鉴定出种子质量以确定其使用价值和价格。

7.2 定义

7.2.1 优良种子

具有下述感官表现的种子:种粒饱满,胚和胚乳发育正常,呈该树种新鲜种子特有的颜色、弹性和气味。

7.2.2 劣质种子

具有下述感官表现的种子:种仁萎缩或干瘪,失去该树种新鲜种子特有的颜色、弹性和气味,或被虫蛀,或有霉坏症状,或有异味,或已霉烂。

7.3 测定用工具

解剖刀,解剖剪,镊子,锤子,放大镜,玻杯,铝盒,载玻片等。

7.4 测定样品

根据第3章的规定从种批抽样,取得送检样品。从经过充分混合的送检样品中随机数取100粒(种粒大的取50粒或25粒),作为一个重复,共取4个重复。种皮坚硬难于剖切的,可在测定前浸种,使种皮软化。

7.5 方法

采用解剖法。

先观察供测种子的外部情况,然后分别逐粒剖开,观察种子内部情况。根据7.2.1和7.2.2所列定义区分优良种子与劣质种子。附录B表B5给出了130个树种优良种子的鉴别特征。表B5未列出的树种可以参照近似种或根据检验机构的经验判断,但应在检验证书中注明"所检树种未列入GB 2772—1999"。各个重复的优良种子、劣质种子以及剖切时发现的空粒、涩粒、无胚粒、腐烂粒和虫害粒的数量记入附录C表C5中。空粒、涩粒、无胚粒和虫害粒的定义见5.2.7.d)。

7.6 计算结果

测定结果以优良种子的百分率表示,分别计算各个重复的百分率,并按表5检查各次重复间的差距是否为随机误差,如果各重复中最大与最小值之差没有超过容许差距范围,就用各重复的平均数作为该种批的优良度。平均数带有的小数按GB/T 8170修约为整数。

如果各重复中最大值与最小值之差超过表5所列的容许范围,应按5.7e)的规定重新测定并计算结果。

7.7 结果报告

预处理情况和计算结果填在质量检验证书上。

8 种子健康状况测定

8.1 目的

测定种子样品的健康状况,为评估种子质量提供依据,从而提出对种子的处理意见。

8.2 定义

8.2.1 种子健康状况:种子健康状况主要是指是否携带病原菌,如真菌、细菌、病毒,以及害虫。

8.2.2 培养:将种子保持在有利于病原体发育或病症发展的环境条件下进行培养。

8.3 原则

检查测定样品中是否存在送检人指明的病原体和害虫，按所用方法允许的程度尽可能准确地估测样品中受感染的种子数。

种批如经过处理，可能会影响测定。凡经过处理的，要求送检人说明处理方式和所用化学药品。

8.4 程序

8.4.1 直观检查：将测定样品放在白纸、白瓷盘或玻璃板上，挑出菌核、霉粒、虫瘿、活虫及病虫伤害的种子，分别计算病虫害感染度。如挑出的菌核、虫瘿、活虫数量多时，应分别统计。

8.4.2 种子中隐蔽害虫的检查：在送检样品中，随机抽取测定样品 200 粒或 100 粒，选用下列方法进行测定。

a）剖开法　切开种子检查；

b）染色法　用高锰酸钾等化学药品染色检查；

c）比重法　利用饱和食盐水或其他药液的浮力检查（适用于豆科等比重较大的种子，如刺槐，浮在上面的种粒多为受虫害的，结合剖开法即可确定虫害粒数）；

d）X 射线透视检查。

8.4.3 如需测定种子病害原因或携带的病原体，可用适当倍数的显微镜直接检查。有条件的，可进行洗涤检查和分离培养检查。

8.5 结果计算和表示

8.5.1 病害感染度计算

$$病害感染度(\%)=\frac{霉粒数+病害粒数}{测定样品粒数}\times 100 \quad\cdots\cdots\cdots\cdots(4)$$

8.5.2 虫害感染度计算

$$虫害感染度(\%)=\frac{虫害粒数}{测定样品粒数}\times 100 \quad\cdots\cdots\cdots\cdots(5)$$

8.5.3 送检样品病、虫害感染度是式(4)和式(5)两者之和。

8.6 结果报告

将测定结果填入质量检验证书上，并提出处理意见。凡能识别的病、虫，应记载其种类；不能识别的必要时送有关部门鉴定。

9 含水量测定

9.1 目的

使用适合于常规检验的方法测定种子的水分含量。

9.2 定义

样品的含水量是指采用本标准的方法将种子样品烘干所失去的重量，用这个重量占样品原始重量的百分率表示。

9.3 原则

测定方法应在尽可能多地除去水分的同时，减少样品的氧化、分解或其他挥发性物质的损失。

9.4 仪器

a）恒温烘箱及其附件（包括样品盒和干燥器）；

b）分析天平；

c）筛子。

9.5 程序

9.5.1 注意事项

供水分测定的送检样品（含水量送检样品重量见 3.3.3.2），必须装在防潮容器中，尽可能排除其中的空气。测定应在样品接收以后尽快开始。测定时，样品曝露在实验室空气中的时间应减少至最低限度。

对于不需要切片的种子，从接收到的容器中取出样品，直至样品密闭在准备烘干的样品盒内，所经历的时间不得超过 2 min。

9.5.2 称重

称重以克为单位，保留三位小数。

9.5.3 测定样品

测定应取两份独立分取的重复样品，根据所用样品盒直径的大小，每份样品重量为：

直径小于 8 cm：4～5 g；直径等于或大于 8 cm：10 g。

在分取测定样品以前，送检样品须按下列方法之一进行充分混合：

a）用匙在样品罐内搅拌；

b）将原样品容器的口对准另一个同样大小的空容器口，把种子在两个容器中往返倾倒。每个测定样品应按检验规程 3.4.2 所规定方法取得，样品曝露在空气中时间应尽可能地缩短。

9.5.4 切片

大粒种子（每千克少于 5 000 粒）以种及皮坚硬的种子（如豆科），每个种粒应当切成小片。粒径等于或大于 15 mm 的种子应至少切成 4 或 5 片。切片动作要快。

落入容器中的切片用骨勺迅速搅拌，并从中随机提取大致相当于 5 粒完整种子重量的测定样品。整个操作中曝露在空气里的时间不得超过 60 min。

9.5.5 烘干

9.5.5.1 低恒温烘干法

此法适用于所有的林木种子。按 9.5.3 和 9.5.4 取得的测定样品，必须均匀地铺在样品盒里。在盛入样品之前，称取样品盒连同盒盖的重量。装入样品后称取样品及样品盒（连同盒盖）的重量。将样品盒迅速置于盖上，放入已经保持在 103℃±2℃的烘箱中烘 17 h±1 h。烘箱回升至所需温度时开始计算烘干时间。达到规定的时间后，迅速盖好样品盒的盖子，并放入干燥器里冷却 30～45 min。

冷却后，称出样品盒连盖及样品的重量。测定时，实验室的空气相对湿度必须低于 70%。

9.5.5.2 高恒温烘干法

其程序与 9.5.5.1 法规定相同，但烘箱温度须保持 130～133℃。样品烘干时间为 1～4 h（具体时间应与 9.5.5.1 对照后确定）。测定时，对实验室的空气相对湿度没有特别要求。

9.5.5.3 预先烘干

含水量高于 17%的种子，在按 9.5.5.1 烘干前应当经受预先烘干。称取两个预备样品，每个样品至少称取 25 g±0.2 mg，放入已称过重量的样品盒内，在 70℃的烘箱中预烘 2～5 h，使水分降至 17%以下，取出后置于干燥器内冷却，称重。将预烘过的种子切片，称取测定样品，用低恒温烘干法或高恒温烘干法测定含水量。对于需要切片的种子预先烘干并非强制性要求。

9.5.5.4 使用其他方法测定含水量时，需与低恒温烘干法（9.5.5.1）相对照并在质量检验证书中说明所用方法。

9.6 结果计算

9.6.1 恒温烘干

含水量以重量百分率表示，用式（6）计算到一位小数：

$$含水量(\%) = \frac{M_2 - M_3}{M_2 - M_1} \times 100 \qquad \cdots\cdots(6)$$

式中：M_1——样品盒和盖的重量，g；

M_2——样品盒和盖及样品的烘前重量，g；

M_3——样品盒和盖及样品的烘后重量，g。

9.6.2 预先烘干

可按式（7）从第一次（预先烘干）和第二次（恒温烘干）所得结果计算样品含水量。式（7）中 S_1 是第一

·次失去的水分，S_2是第二次失去的水分，两次均按上法计算，以百分率表示：

$$含水量(\%) = S_1 + S_2 - \frac{S_1 \times S_2}{100} \quad \cdots\cdots (7)$$

9.6.3 容许差距

依据种子大小和原始水分的不同，两个重复间的容许差距范围为0.3%～2.5%，见表8。

表8 含水量测定两次重复间的容许差距

种子大小类别	平均原始水分		
	<12%	12%～25%	>25%
1	2	3	4
小种子[1]	0.3%	0.5%	0.5%
大种子[2]	0.4%	0.8%	2.5%

1）小种子是指每千克超过5 000粒的种子。

2）大种子是指每千克最多为5 000粒的种子。

9.7 结果报告

含水量测定结果在质量检验证书上填报，精度为0.1%。

10 重量测定

10.1 目的

测定送验样品每1 000粒种子的重量。

10.2 原则

从纯净种子中数取一定数量的种子，称重，并计算每1 000粒种子的重量。

10.3 仪器

可以采用发芽测定使用的数粒器。

10.4 程序

可以采用整个测定样品(10.4.2)，也可以采用从中分取的若干个重复(10.4.3)。

10.4.1 测定样品

按本规程第4章，以净度分析后的全部纯净种子作为测定样品。

10.4.2 对整个测定样品计数并称重

将整个测定样品通过仪器，并读出在数粒器上显示的种子粒数。也可以人工计数。将计数后的测定样品称重(g)，小数的位数与净度分析(表1)相同。

10.4.3 按重复计数并称重

用手或数粒器，从测定样品中随机数取8个重复，每个重复100粒，各重复分别称重(g)，小数的位数与净度分析(表1)相同。

计算方差、标准差、变异系数和平均重量如下：

$$方差 = \frac{n(\Sigma x^2) - (\Sigma x)^2}{n(n-1)} \quad \cdots\cdots (8)$$

式中：x——每个重复的重量，g；

n——重复次数。

$$标准差(S) = \sqrt{方差} \quad \cdots\cdots (9)$$

$$变异系数 = \frac{S}{\bar{x}} \times 100 \quad \cdots\cdots (10)$$

式中：$\bar{x}$——100粒种子的平均重量。

种粒大小悬殊的种子和粘滞性种子，变异系数不超过6.0，一般种子的变异系数不超过4.0，就可计

算测定结果。如变异系数超过上述限度,则应再数取 8 个重复,称重,并计算 16 个重复的标准差。凡与平均数之差超过两倍标准差的各重复舍弃不计,将剩下的各个重复用于计算。

10.5 结果计算和表示

按 10.4.2 数取并称量整个测定样品的重量,根据整个测定样品的粒数和重量换算出 1 000 粒种子的重量。

按 10.4.3 数取并称量各个重复的重量,将 8 个或 8 个以上的各个重复 100 粒的重量换算成 1 000 粒种子的重量(即 $10\times\bar{x}$)。

计算结果的小数位数与表 1 的规定相同。

10.6 结果报告

重量测定计算结果填写在质量检验证书上。

11 X 射线检验

11.1 目的

X 射线摄影检验的目的是:

a) 以 X 射线图像可见的形态特征为依据,为区分饱满种子、空瘪种子、虫害种子和机械损伤种子提供一种无损的快速检测方法;

b) 为饱满种子、空瘪种子、虫害种子和机械损伤种子在样品中所占的比例建立一个永久性的摄影图像记录。

11.2 定义

11.2.1 射线图像

射线图像是位于感光胶片(或相纸)同 X 射线源之间的物体在感光胶片(或相纸)上所成的像。照相冲洗技术可以把潜像变成可见图像。

11.2.2 X 射线

X 射线是电磁波谱中以光速移动的电磁波,所不同的是波长,X 射线的波长只相当于光的波长的 1/10 000~1/100 000。高能量的 X 射线波长较短,比较适合于拍摄大而(或)致密的物体。低能量的 X 射线波长较长,适合于拍摄小的物体,例如种子。

11.3 基本原理

种子放在低能量 X 射线源和感光胶片(或相纸)之间。种子各种组织吸收的 X 射线量不同,取决于它们的厚度和(或)密度。光敏成像乳胶被激活的程度随所接受的辐射量而异,由此造成潜像。冲洗胶片或相纸,便会形成明暗深浅不同的可见图像。以下几个因素会影响 X 射线图像的质量。

电压(kV)。千伏乃是 X 射线管中两个电极之间电位的度量。提高电压会生成波长较短的 X 射线。电压影响图像的反差:较低的电压会改善分辨率,较高的电压则降低密度差。

电流(mA)。毫安乃是对 X 射线管施加的电流的度量。提高电流会增加一定时间内生成的 X 射线的数量。电流影响图像的密度,但不影响图像的反差。电流太高会使图像曝光过度(变黑)。

曝光时间(s)。曝光时间乃是拍摄射线图像时样品接受 X 射线的时间。调整曝光时间会改变图像密度。一定的曝光量所需的毫安数同曝光时间成反比。因此曝光量应当用毫安秒(mAs)或毫安分(mAm)表示。为了保持相同的图像质量,增加曝光时间必须相应地降低电流。例如,5 mA 的管电流和 20 s 的曝光时间得到 100 mAs 的曝光量,所生成的图像密度同 10 mA 曝光 10 s 是一样的。

焦斑(即电流靶)同胶片表面之间的距离为焦片距(FFD)。加大 FFD 就会按平方反比定律降低辐射强度。因此,将 FFD 加倍(2FFD),就要将曝光量变成四倍(4 mAs),才能在胶片或相纸上得到相同程度的图像密度。被摄物体同胶片表面的距离称为物片距(OFD)。OFD 影响图像质量:距离越大,细部越模糊,因而图像质量越差。如果要求细部清晰,种子可以直接放在胶片表面。不过常规作业中通常都是把胶片保藏在封套里,便于操作。

有些衬比剂在物体中的渗透状况不同，为了加强图像的某些特征，采用衬比剂有可能使物体某些部位比其余部位的射线图像密度大。

11.4 设备

下列设备为必需设备：

X射线机；

胶片或相纸，显影剂，定影剂；

X射线胶片或相纸；

胶片暗袋；

种子托板。

11.5 程序

11.5.1 将胶片或相纸装入暗盒或暗袋，或者使用本身已带包装的胶片或相纸。

11.5.2 射线检验采用4个重复，每个重复100粒种子，都从纯净种子中随机提取。X射线检验的种子可以是即将用于发芽测定的种子。

11.5.3 使用托板（也可以不用托板），将种子均匀地排放在胶片或相纸上面。

11.5.4 在胶片或相纸上摆放铅字或不透X射线的其他标记物，以便区分样品。

11.5.5 曝光。为了生成最佳图像，不同的X射线机可能要求各不相同的曝光时间和电压组合。树种不同组合也不同。为了得到最佳结果，每当使用新的材料或采用新的射线机时，应当就曝光时间、电压和曝光量先做试验。

11.5.6 冲洗胶片或相纸。相纸通常是用快速显影剂来显影的，这种快速显影剂可以在数秒钟内显出影像。胶片则必须在暗室显影。

11.6 图像判读

根据射线图像显示的内部结构将种子归类。

11.6.1 饱满粒：具有发芽所需的各种基本组织的种子或果实。

11.6.2 空粒：所含种子组织小于50%的种子或果实。

11.6.3 虫害粒：内含成虫、幼虫、虫粪或有其他迹象表明遭受过虫害足以影响种子发芽能力的种子或果实。

11.6.4 机械损伤粒：皮壳开裂或破损的饱满粒。

11.7 结果报告

检验结果以饱满粒、空粒、虫害粒和机械损伤粒的百分率表示，并在质量检验证书"备注"栏填写。填报形式如下：

X射线检验结果：

饱满粒占________%；

空粒占________%；

虫害粒占________%；

机械损伤粒占________%。

12 质量检验证书

12.1 目的

本章的目的是为签发林木种子质量检验证书制定规则。本标准规定的质量检验证书只能在按照本规定抽样、检验后，由林业主管部门授权或国家技术监督部门依法设置的检验机构签发。质量检验证书分为种批质量检验证书和种子样品质量检验证书两类。

12.1.1 种批质量检验证书

送检样品由授权的检验机构自身或在其监督下，按本标准规定的程序和方法从种批中抽取送检样

品，由授权的检验机构检验后签发的质量检验证书。

12.1.2 种子样品质量检验证书

授权的检验机构对非自身或未在其监督下，抽取的送检样品检验后签发的质量检验证书。检验机构只对送检样品的检验负责，不对送检样品的代表性负责。

12.1.3 质量检验证书的副本

除印有醒目的“副本”字样外，形式和内容与所签发的质量检验证书完全相同。

12.2 原则

如果对下面各条的确切含义存有疑问，应当按照符合下列原则的理解进行解释。

12.2.1 只有按照与所签发的那类质量检验证书相符的规则进行抽样和检验时，才能签发该类质量检验证书。

12.2.2 签发种批质量检验证书时，该种批每个容器必须封缄，并标有与质量检验证书上相同的标记或编号，清楚地表明质量检验证书所指的是哪些容器。

12.2.3 一份质量检验证书始终有效，除非后来被同一种批签发的质量检验证书所替代。

12.2.4 对一个种批的任何质量项目，在同一时期内，有效的种批质量检验证书不能多于一份。

12.2.5 质量检验证书可以用中文或中、英两种文字印制。

12.3 签发质量检验证书的条件

12.3.1 质量检验证书只能由检验机构签发，并具备下列条件：

——该机构经林业主管部门授权或国家技术监督部门依法设置，目前从事这项工作。

——被检种是本规程所列的种，未列的种也可以检验，但应在质量检验书上说明：“送检树种是国家标准 GB 2772—1999 没有列入的树种，本次检验条件为：　　　。”

——检验是按本标准规定的程序方法进行的。但应送检者要求，采用本标准未规定的程序和方法检验时，检验结果也可填报。

12.3.2 抽样时把一个种批视为一个整体，抽样人员对样品的代表性负责，检验人员对样品的检验结果负责。

12.3.2.1 种批质量检验证书上填报的结果是指抽样时该种批的整体。

12.3.2.2 种子样品质量检验证书上填写的结果是指收到时的送检样品。

12.3.3 如果根据种子形态不能确定被检种的种名，质量检验证书中可以填写属名，例如在中文栏填写“松属的一种”，在学名栏填写“*Pinus* sp.”，遇有同属种子混杂的情况，可以填写“松属的几个种”和“*Pinus* spp.”。

12.3.4 所有日期都按国际标准化组织(ISO)规定填写：年填四位数，月、日填两位数，年和月、月和日之间用短横连接，如 1995-08-01。

12.4 质量检验证书的格式和内容

12.4.1 质量检验证书中除写送检者和地址外，还应按送检者的陈述写入必要的信息。在种批质量检验证书中，这种信息是指供检种的中名、学名和产地。在种子样品质量检验证书中，这种信息是指供检种的中名、学名、产地、种批重、种批编号、容器件数、抽样日期和送检单位对送检样品的编号。

12.4.2 种批质量检验证书正式报告部分需填报：

——抽样和封缄的机构和人员；

——种批的正式标记和封缄情况；

——种批的重量和容器件数；

——抽样日期；

——样品收到日期和检验结束日期；

——样品重量和样品编号；

——检验结果；

——签发机构的名称和地址；

——签发日期；

——签发机构的声明(见附录D表D2种批质量检验证书的背面)。

12.4.3 种子样品质量检验证书需填报：

——送检样品编号；

——送检样品封缄情况；

——送检样品重量；

——送检样品收到日期；

——检验结束日期；

——检验结果；

——签发机构的名称和地址；

——签发日期；

——签发机构的声明(见附录D表D1种子样品质量检验证书的背面)。

12.4.4 根据送检者的要求，可以签发质量检验证书“副本”。

12.5 检验结果填报

12.5.1 一次抽样只能提交一个送检样品，根据送检者的要求，这个送检样品可按本标准所规定的一种或几种方法予以测定。某些项目的测定，例如含水量和种子健康状况测定，则要求在同一份混合样品中分取另外的样品，专门包装送检。

12.5.2 一份送检样品各个检验项目的检验结果，可以合并填报于一份，或分别填报于多份检验证书上。但填报发芽率、生活力、优良度或X射线测定结果时，应同时填报净度测定结果。

12.5.3 一个检测项目有几种测定方法时，应说明测定方法。

12.5.4 检验结果的填报应当符合本规程有关各章对检验结果计算、表述和填报的规定。未测定的项目在质量检验证书的相应栏中填写“未测定”或“N”字样。

12.5.5 根据GB 7908对检验结果划分等级。对三级以下种子，可根据具体情况提出处理意见。

12.6 质量检验证书的效力

12.6.1 不允许同一检验机构在前次抽样后的一个月内对同一种批再次抽样(或监督抽样)、检验并签发同一类型的检验证书。但如果前次检验后种批经过重新处理则属例外。

12.6.2 如果要求签发不同类型的证书，或证书将由另一检验机构签发，或12.6.1规定的一个月的期限已经超过，可以签发另一份检验证书，但需注明原检验证书类别、编号和原抽样日期，或对该种批重新编写标签、重新封缄。如果种批的标记情况未变，就应当声明原证书作废，应以后面这份证书中填写的检验结果为准。声明的方式可以是：“本种批曾于____(年、月、日)抽样，所发的种批证书号为____。该证书上的检验结果至此作废。”

12.6.3 检验证书应当有签发机构的名称及其地址和签发日期，并经签发机构加盖公章、技术负责人签名后才能生效。签发的证书不得有增删、修改、涂抹痕迹，否则无效。

12.6.4 抽样时把该种批视为一个整体(并非某一部分)。如果该批种子分别调往不同用种单位，应重新抽样检验，原检验证书无效。

12.7 结果争议

如果另一授权检验机构检验结果与原质量检验证书中填报的结果抵触，可由双方共同的上一级授权检验机构进行仲裁检验。

附 录 A
（标准的附录）
种批和样品重量表

表 A1

顺序号	树 种	种批的最大重量,kg	样品最低重量,g	
			送检样品	净度分析测定样品
1	冷 杉<br>*Abies fabri* (Mast.) Craib	1 000	100	50
2	岷江冷杉<br>*A. faxoniana* Rehd. et Wils.	1 000	50	30
3	日本冷杉<br>*A. firma* Sieb. et Zucc.	1 000	200	100
4	杉松(沙松)<br>*A. holophylla* Maxim.	1 000	250	150
5	雪 松<br>*Cedrus deodara* (Roxb.) G. Don	1 000	600	300
6	日本扁柏<br>*Chamaecyparis obtusa* (Sieb. et Zucc.) Endl.	250	12	6
7	日本花柏<br>*Ch. Pisifera* Endl.	250	10	3
8	柳 杉<br>*Cryptomeria fortunei* Hooibrenk	1 000	20	10
9	日本柳杉<br>*C. japonica* (L. f.) D. Don	1 000	20	10
10	杉 木<br>*Cunninghamia lanceolata* (Lamb.) Hook.	1 000	50	30
11	干香柏(冲天柏)<br>*Cupressus duclouxiana* Hickel	1 000	35	15
12	柏 木<br>*C. funebris* Endl.	1 000	35	15
13	福建柏<br>*Fokienia hodginsii* (Dunn.) Henry et Thomas	1 000	60	25
14	银 杏<br>*Ginkgo biloba* L.	10 000	>500 粒	>500 粒
15	落叶松(兴安落叶松)<br>*Larix gmelinii* (Rupr.) Rupr.	1 000	25	10
16	日本落叶松<br>*L. kaempferi* (Lamb.) Carr.	1 000	25	10
17	四川红杉<br>*L. mastersiana* Rehd. et Wils.	1 000	25	10
18	黄花落叶松(长白落叶松)<br>*L. olgensis* Henry	1 000	25	10

表 A1（续）

顺序号	树　　种	种批的最大重量,kg	样品最低重量,g	
			送检样品	净度分析测定样品
19	红　杉<br>*L. potaninii* Batal.	1 000	25	10
20	华北落叶松<br>*L. principis-rupprechtii* Mayr.	1 000	25	10
21	西伯利亚落叶松<br>*L. sibirica* (Munchh.) Ledeb.	1 000	25	10
22	水　杉<br>*Metasequoia glyptostroboides* Hu et Cheng	250	15	5
23	云　杉<br>*Picea asperata* Mast.	1 000	25	7
24	鱼鳞云杉<br>*P. jezoensis* var. *microsperma* (Lindl.) Cheng et L. K. Fu	1 000	25	7
25	红皮云杉<br>*P. koraiensis* Nakai	1 000	25	9
26	白　杆<br>*P. meyeri* Rehd. et Wils.	1 000	35	15
27	天山云杉<br>*P. schrenkiana* var. *tianshanica* Cheng et S. H. Fu	1 000	25	9
28	青　杆<br>*P. wilsonii* Mast.	1 000	35	15
29	华山松<br>*Pinus armandi* Franch.	3 500	1 000	700
30	白皮松<br>*P. bungeana* Zucc. ex Eandl.	3 500	850	500
31	加勒比松<br>*P. caribaea* Morele.	1 000	100	50
32	赤　松<br>*P. densiflora* Sieb. et Zucc.	1 000	60	30
33	萌芽松<br>*P. echinata* Mill.	1 000	4	5
34	湿地松<br>*P. elliottii* Engelm.	1 000	160	80
35	思茅松<br>*P. kesiya* Royle ex Gord. var. *langbianensis* (A. Chev.) Gaussen	1 000	85	35
36	红　松<br>*P. koraiensis* Sieb. et Zucc.	5 000	2 000	1 000
37	南亚松<br>*P. latteri* Mason	1 000	120	60
38	马尾松<br>*P. massoniana* Lamb.	1 000	85	35

表 A1（续）

顺序号	树种	种批的最大重量,kg	样品最低重量,g 送检样品	样品最低重量,g 净度分析测定样品
39	卵果松<br>*P. oocarpa* Schiede	1 000	70	35
40	长叶松<br>*P. palustris* Mill.	1 000	500	250
41	日本五针松<br>*P. parviflora* Sieb. et Zucc.	1 000	500	250
42	展叶松<br>*P. patula* Schlecht. et Cham.	1 000	40	20
43	辐射松<br>*P. radiata* D. Don	1 000	160	80
44	刚　松<br>*P. rigida* Mill.	1 000	50	30
45	晚　松<br>*P. rigida* Mill. var. *serotina* (Michx.) Loud. ex Hoopes	1 000	85	35
46	樟子松<br>*P. sylvestris* var. *mongolica* Litv.	1 000	40	20
47	油　松<br>*P. tabulaeformis* Carr.	1 000	100	50
48	火炬松<br>*P. taeda* L.	1 000	140	70
49	黄山松<br>*P. taiwanensis* Hayata	1 000	100	50
50	黑　松<br>*P. thunbergii* Parl.	1 000	85	35
51	云南松<br>*P. yunnanensis* Franch.	1 000	85	35
52	侧　柏<br>*Platycladus orientalis* (L.) Franco	1 000	120	60
53	竹　柏<br>*Podocarpus nagi* (Thunb.) Zoll. et Mor.	5 000	1 200	1 000
54	大叶竹柏<br>*P. olifera* Tsiang et Chun	500	>500 粒	>500 粒
55	金钱松<br>*Pseudolarix kaempferi* (Lindl.) Gord.	1 000	200	100
56	圆　柏<br>*Sabina chinensis* (L.) Ant.	1 000	180	90

表 A1（续）

顺序号	树　　种	种批的最大重量,kg	样品最低重量,g	
			送检样品	净度分析测定样品
57	铅笔柏<br>*S. virginiana*(L.) Ant.	1 000	70	35
58	池　杉<br>*Taxodium ascendens* Brongn.	1 000	500	250
59	落羽杉<br>*T. distichum* (L.) Rich.	1 000	500	250
60	金合欢属<br>*Acacia* spp.	1 000	70	35
61	台湾相思(相思树)<br>*A. richii* A. Gray	1 000	200	80
62	黑荆树<br>*A. mearnsii* De Wild.	1 000	70	35
63	鸡爪槭<br>*Acer palmatum* Thunb.	10 000	100	50
64	元宝枫<br>*A. truncatum* Bunge	3 500	850	400
65	七叶树<br>*Aesculus chinensis* Bunge	10 000	>500 粒	>500 粒
66	臭　椿<br>*Ailanthus altissima* (Mill.) Swingle	1 000	160	80
67	合　欢<br>*Albizia julibrissin* Durazz.	1 000	200	100
68	三年桐<br>*Aleurites fordii* Hemsl.	10 000	>500 粒	>500 粒
69	千年桐<br>*A. montana* (Lour.) Wils.	10 000	>500 粒	>500 粒
70	桤　木<br>*Alnus cremastogyne* Burkill	250	25	4
71	旱冬瓜(蒙自桤木)<br>*A. nepalensis* D. Don	250	12	6
72	紫穗槐<br>*Amorpha fruticosa* L.	1 000	85	50
73	腰　果<br>*Anacardium occidentale* L.	10 000	>300 粒	>300 粒
74	团　花<br>*Anthocephalus chinensis* (Lam.) A. Rich. ex Walp.	250	6	1
75	木菠萝<br>*Artocarpus heterophyllus* Lam.	10 000	>300 粒	>300 粒
76	羊蹄甲<br>*Bauhinia purpurea* L.	3 500	1 000	700
77	垂枝桦<br>*Betula pendula* Roth	250	10	1
78	白　桦<br>*B. platyphylla* Suk.	250	10	1

表 A1（续）

顺序号	树　　种	种批的最大重量,kg	样品最低重量,g	
			送检样品	净度分析测定样品
79	油　茶<br>*Camellia oleifera* Abel.	5 000	>500 粒	>500 粒
80	茶<br>*C. sinensis* Kuntze	5 000	>500 粒	>500 粒
81	喜　树<br>*Camptotheca acuminata* Decne.	1 000	200	100
82	柠条锦鸡儿<br>*Caragana korshinskii* Kom.	1 000	200	100
83	小叶锦鸡儿<br>*C. microphylla* Lam.	1 000	200	100
84	薄壳山核桃<br>*Carya illinoensis* (Wangenh.)<br>K. Koch	10 000	>300 粒	>300 粒
85	铁刀木<br>*Cassia siamea* Lam.	1 000	200	80
86	锥　栗<br>*Castanea henryi* (Skan)<br>Rehd. et Wils.	10 000	>500 粒	>500 粒
87	板　栗<br>*C. mollissima* Blume	10 000	>300 粒	>300 粒
88	红　椎<br>*Castanopsis hystrix* A. DC.	5 000	1 200	900
89	青钩栲(格氏栲)<br>*C. kawakamii* Hay.	10 000	>500 粒	>500 粒
90	木麻黄<br>*Casuarina equisetifolia* L.	250	15	2
91	梓　属<br>*Catalpa* spp.	1 000	120	60
92	麻　楝<br>*Chukrasia tabularis* A. Juss.	1 000	85	35
93	樟　树<br>*Cinnamomum camphora* (L.) Presl	3 500	600	300
94	肉　桂<br>*C. cassia* Presl	3 500	1 000	600
95	银　木<br>*C. septentrionale* Hand. -Mazz	3 500	600	300
96	南岭黄檀<br>*Dalbergia balansae* Prain	1 000	250	150
97	降香黄檀<br>*D. odorifera* T. Chen	1 000	1 000	500
98	凤凰木<br>*Delonix regia* (Bojea.) Raf.	3 500	1 200	900
99	君迁子<br>*Diospyros lotus* L.	1 000	400	250
100	坡柳(车桑子)<br>*Dodonaea viscosa* (L.) Jacq.	250	5	2

表 A1（续）

顺序号	树种	种批的最大重量,kg	样品最低重量,g	
			送检样品	净度分析测定样品
101	沙枣<br>*Elaeagnus angustifolia* L.	1 000	800	400
102	翅果油树<br>*E. mollis* Diels	5 000	1 200	900
103	泡火绳<br>*Eriolaena malvacea* (Levl.) Hand.-Mzt.	1 000	70	35
104	格木<br>*Erythrophleum fordii* Oliv.	5 000	>500 粒	>500 粒
105	赤桉<br>*Eucalyptus camaldulensis* Dehnh	250	15	—
106	柠檬桉<br>*Eu. citriodora* Hook.	1 000	40	15
107	窿缘桉<br>*Eu. exserta* F. Muell.	250	6	—
108	蓝桉<br>*Eu. globulus* Labill.	250	60	—
109	葡萄桉<br>*Eu. botryoides* Sm.	250	6	—
110	直干蓝桉<br>*Eu. maidenii* F. V. M.	250	40	15
111	王桉<br>*Eu. regnans* F. Muell	250	30	10
112	大叶桉<br>*Eu. robusta* Smith	250	15	—
113	蜡皮桉<br>*Eu. rubida* Decne et Maiden	250	15	—
114	谷桉<br>*Eu. smithii* R. T. Baker	250	30	10
115	细叶桉<br>*Eu. tereticornis* Smith	250	15	—
116	多枝桉<br>*Eu. viminalis* Labill	250	30	10
117	杜仲<br>*Eucommia ulmoides* Oliv.	1 000	400	250
118	梧桐<br>*Firmiana simplex* (L.) W. F. Wight	3 500	850	500
119	白蜡树<br>*Fraxinus chinensis* Roxb.	1 000	200	100
120	水曲柳<br>*F. mandshurica* Rupr.	1 000	400	200
121	皂荚<br>*Gleditsia sinensis* Lam.	3 500	1 200	800

表 A1（续）

顺序号	树种	种批的最大重量,kg	样品最低重量,g	
			送检样品	净度分析测定样品
122	云南石梓<br>*Gmelina arborea* Roxb.	3 500	1 200	900
123	海南石梓<br>*G. hainanensis* Oliv.	3 500	1 200	900
124	银　桦<br>*Grevillea robusta* A. Cunn.	1 000	85	35
125	梭　梭<br>*Haloxylon ammodendron* (Mey.) Bunge	1 000	35	15
126	白梭梭<br>*H. persicum* Bunge ex Boiss. et Buhse	1 000	35	15
127	蒙古岩黄蓍(羊柴)<br>*Hedysarum mongolicum* Turcz.	1 000	160	80
128	花棒(细枝岩黄蓍)<br>*H. scoparium* Fisch. et Mey.	1 000	200	100
129	沙　棘<br>*Hippophae rhamnoides* L.	1 000	85	35
130	红花天料木(母生)<br>*Homaliun hainanense* Gagnep.	250	15	1
131	坡　垒<br>*Hopea hainanensis* Merr. et Chun	5 000	1 200	900
132	核　桃<br>*Juglans rigia* L.	10 000	＞300 粒	＞300 粒
133	非洲桃花心木<br>*Khaya senegalensis* A. Juss.	3 500	850	500
134	栾　树<br>*Koelreuteria paniculata* Laxm.	1 000	800	400
135	紫　薇<br>*Lagerstroemia indica* L.	250	15	5
136	胡枝子<br>*Lespedeza bicolor* Turcz.	1 000	60	25
137	枫　香<br>*Liquidambar formosana* Hance	1 000	35	15
138	鹅掌楸<br>*Liriodendron chinense* (Hemsl.) Sarg.	1 000	180	90
139	北美鹅掌楸<br>*L. tulipifera* L.	1 000	180	90
140	金银花(忍冬)<br>*Lonicera japonica* Thunb.	1 000	35	15
141	枸　杞<br>*Lycium chinense* Mill.	250	15	15

表 A1（续）

顺序号	树种	种批的最大重量,kg	样品最低重量,g	
			送检样品	净度分析测定样品
142	绿楠(海南木莲)<br>*Manglietia hainanensis* Dandy	1 000	200	100
143	楝 树<br>*Melia azedarach* L.	5 000	>500 粒	>500 粒
144	川 楝<br>*M. toosendan* Sieb. et Zucc.	5 000	>500 粒	>500 粒
145	醉香含笑(火力楠)<br>*Michelia macclurei* Dandy	3 500	600	300
146	桑 属<br>*Morus* spp.	250	20	5
147	壳菜果(米老排)<br>*Mytilaria laosensis* Lec.	3 500	850	500
148	兰考泡桐<br>*Paulownia elongata* S. Y. Hu	250	6	1
149	白花泡桐<br>*P. fortunei* (Seem.) Hemsl.	250	6	1
150	毛泡桐<br>*P. tomentosa* (Thunb.) Steud.	250	6	1
151	黄菠萝(黄檗)<br>*Phellodendron amurense* Rupr.	1 000	85	50
152	毛 竹<br>*Phyllostachys pubescens*<br>Mazel ex H. de Lehaie	1 000	85	50
153	黄连木<br>*Pistacia chinensis* Bunge	1 000	350	200
154	悬铃木属<br>*Platanus* spp.	250	25	6
155	杨属<br>*Populus* spp.	250	5	2
156	山 杏<br>*Prunus armeniaca* var.<br>*ansu* Maxim.	5 000	>500 粒	>500 粒
157	山 桃<br>*P. davidiana* (Carr.) Franch.	10 000	>500 粒	>500 粒
158	枫 杨<br>*Pterocarya stenoptera* C. DC.	1 000	400	200
159	葛 藤<br>*Pueraria lobata* (Willd) Ohwi.	1 000	85	35
160	栎 属<br>*Quercus* spp.	10 000	>500 粒	>500 粒
161	火炬树<br>*Rhus typhina* L.	1 000	50	30
162	刺 槐<br>*Robinia pseudoacacia* L.	1 000	100	50
163	旱 柳<br>*Salix matsudana* Koidz.	250	5	2

表 A1（完）

顺序号	树种	种批的最大重量,kg	样品最低重量,g	
			送检样品	净度分析测定样品
164	乌桕<br>*Sapium sebiferum* (L.) Roxb.	3 500	850	400
165	檫木<br>*Sassafras tsumu* (Hemsl.) Hemsl.	1 000	400	200
166	木荷<br>*Schima superba* Gardn. et Champ.	1 000	35	15
167	箭竹<br>*Sinarundinaria nitida* (Mitf.) Nakai	1 000	35	15
168	槐树<br>*Sophora japonica* L.	3 500	100	50
169	大叶桃花心木<br>*Swietenia macrophylla* King	3 500	1 000	900
170	丁香属<br>*Syringa* spp.	1 000	30	15
171	乌墨(海南蒲桃)<br>*Syzygium cumini* (L.) Skeels	5 000	1 200	900
172	柚木<br>*Tectona grandis* L. f.	5 000	2 000	1 000
173	鸡尖(海南榄仁树)<br>*Terminalia hainanensis Exell.*	3 500	850	350
174	椴属<br>*Tilia* spp.	1 000	500	250
175	香椿<br>*Toona sinensis* (A. Juss.) Roem.	1 000	80	40
176	漆树<br>*Toxicodendron verniciflum* (Stokes) F. A. Barkley	1 000	250	150
177	棕榈<br>*Trachycarpus fortunei* (Hook. f.) H. Wendl.	3 500	1 000	800
178	榔榆<br>*Ulmus parvifolia* Jacq.	1 000	20	8
179	白榆<br>*U. pumila* L.	1 000	30	15
180	青梅<br>*Vatica astrotricha* Hance	3 500	1 000	800
181	文冠果<br>*Xanthoceras sorbifolia* Bunge	5 000	>500 粒	>500 粒
182	大叶榉<br>*Zelkova schneideriana* Hand.-Mzt.	1 000	200	75

附 录 B

（标准的附录）

种子检验技术条件表

B1 发芽测定技术条件见表 B1。

表 B1 发芽测定技术条件表

顺序号	树 种	发芽床	温度 ℃	初次计数，天	末次计数，天	备 注
1	岷江冷杉 *Abies faxoniana* Rehd. et Wils.	纸	25	14	28	始温 45℃水浸种 24 h
2	杉松（沙松） *A. holophylla* Maxim.	纸	20—30	10	28	—
3	柳 杉 *Cryptomeria fortunei* Hooibrenk	纸	25	18	28	—
4	杉 木 *Cunninghamia lanceolata* (Lamb.) Hook.	纸	25	10	21	—
5	干香柏（冲天柏） *Cupressus duclouxiana* Hickel	纸	25	14	28	始温 45℃水浸种 24 h
6	柏 木 *C. funebris* Endl.	纸	20	20	35	—
7	福建柏 *Fakienia hodginsii* (Dunn.) Henry et Thomas	纸	25	14	28	—
8	银 杏 *Ginkgo biloba* L.	沙	25，20—30	14	28	1～5℃层积 60 天
9	落叶松（兴安落叶松） *Larix gmelinii* (Rupr.) Rupr.	纸	20—25	14	28	始温 45℃水浸种 24 h
10	日本落叶松 *L. kaempferi* (Lamb.) Carr.	纸	20	14	21	1～5℃层积 10～15 天
11	四川红杉 *L. mastersiana* Rehd. et Wils.	纸	20	10	24	始温 45℃水浸种 24 h
12	黄花落叶松（长白落叶松） *L. olgensis* Henry	纸	25	14	21	始温 45℃水浸种 24 h
13	红 杉 *L. potaninii* Batal.	纸	20	10	24	始温 45℃水浸种 24 h
14	华北落叶松 *L. principis-rupprechtii* Mayr.	纸	25，20—30	12	21	始温 45℃水浸种 24 h
15	西伯利亚落叶松 *L. sibirica* (Münchh.) Ledeb.	纸	25—30	14	28	始温 45℃水浸种 24 h
16	水 杉 *Metasequoia glyptostroboides* Hu et Cheng	纸	25	10	21	—
17	云 杉 *Picea asperata* Mast.	纸	20～25	10	24	始温 45℃水浸种 24 h

表 B1（续）

顺序号	树　　种	发芽床	温度<br>℃	初次计数，天	末次计数，天	备　　注
18	红皮云杉<br>*P. koraiensis* Nakai	纸	25,20—30	14	21	始温 45℃水浸种 24 h
19	白　杆<br>*P. meyeri* Rehd. et Wils.	纸	25	9	19	—
20	天山云杉<br>*P. schrenkiana* var. *tianshanica* Cheng et S. H. Fu	纸	25	10	25	—
21	青　杆<br>*P. wilsonii* Mast.	纸	20,25	10	21	—
22	华山松<br>*Pinus armandi* Franch	沙	20—30	14	42	1）始温 45℃水浸种 72 h；<br>2）有休眠的种源 1～5℃层积 30 天
23	白皮松<br>*P. bungeana* Zucc. ex Endl.	沙	20—25	14	35	1～5℃层积 45 天
24	湿地松<br>*P. elliottii* Engelm.	纸	25,20—30	14	28	—
25	思茅松<br>*P. kesiya* Royle ex Gord. var. *langbianensis* (A. Chev.) Gaussen	纸	25	10	27	始温 45℃水浸种 24 h
26	南亚松<br>*P. latteri* Mason	纸	30	7	14	—
27	马尾松<br>*P. massoniana* Lamb.	纸	25	10	21	—
28	刚　松<br>*P. rigida* Mill.	纸	20—30	10	16	始温 45℃水浸种 24 h
29	晚　松<br>*P. rigida Mill* var. *serotina* (Michx.) Loud. ex Hoopes	纸	20—30	10	21	—
30	樟子松<br>*P. sylvestris* var. *mongolica* Litv.	纸	25	10	18	—
31	油　松<br>*P. tabulaeformis* Carr.	纸	25	10	21	始温 45℃水浸种 24 h
32	火炬松<br>*P. taeda* L.	纸	25,20—30	14	21	1～5℃层积 28 天
33	黄山松<br>*P. taiwanensis* Hayata	纸	20—30	10	21	—
34	黑　松<br>*P. thunbergii* Parl.	纸	25	14	28	—
35	云南松<br>*P. yunnanensis* Franch.	纸	25	10	27	—
36	侧　柏<br>*Platycladus orientalis* (L.) Franco	纸	25,20—25	14	28	始温 45℃水浸种 24 h

表 B1（续）

顺序号	树　　种	发芽床	温度 ℃	初次计数，天	末次计数，天	备　　注
37	竹　柏 *Podocarpus nagi* (Thunb.) Zoll. et Mor.	沙	25	25	42	—
38	金钱松 *Pseudolarix kaempferi* (Lindl.) Gord.	纸	20,20—30	21	35	—
39	铅笔柏 *Sabina virginiana* (L.) Ant.	纸	15	14	28	20℃层积 60 天后转入 3～5℃下 45 天
40	池　杉 *Taxodium ascendens* Brongn.	沙	20～30	14	28	1) 室温水浸 2 周，每天换水；2) 1～5℃层积 90 天；3) 染色法测定生活力
41	落羽杉 *T. distichum* (L.) Rich	纸	20,20—30	7	28	1) 3～5℃层积 30 天；2) 染色法测定生活力
42	台湾相思（相思树） *Acacia richii* A. Gray	纸	25	7	21	1) 始温 100℃水浸种 2 min，自然冷却 24 h；2) 浓硫酸浸种 15～20 min 后充分冲洗；3) 染色法测定生活力
43	黑荆树 *A. mearnsii* De Wild.	纸	30	7	21	1) 沸水浸种 5 min，自然冷却 24 h；2) 浓硫酸浸种 15～20 min 后充分冲洗；3) 染色法测定生活力
44	元宝枫 *Acer truncatum* Bunge	纸	25	7	14	去翅，室温水浸 2 天后剥去果皮及种皮
45	臭　椿 *Ailanthus altissima* (Mill.) Swingle	纸	30	10	16	去翅，始温 45℃水浸种 24 h
46	合　欢 *Albizia julibrissin* Durazz.	纸	20—30	7	14	始温 80℃水浸种 24 h，余硬粒再处理 1～2 次
47	三年桐 *Aleurites fordii* Hemsl.	纸	25	14	21	去掉种皮
48	千年桐 *A. montana* (Lour.) Wils.	纸	25	14	21	去掉种皮
49	桤　木 *Alnus cremastogyne* Burkill	纸	25	7	21	—
50	旱冬瓜（蒙自桤木） *A. nepalensis* D. Don	纸	25	7	21	—
51	紫穗槐 *Amorpha fruticosa* L.	纸	20—25	7	14	去外种皮，始温 60℃水浸种 24 h
52	腰　果 *Anacardium occidentale* L.	纸	20—30	7	18	—
53	团　花 *Anthocephalus chinensis* (Lam.) A. Rich ex Walp.	纸	25	14	28	1～5℃层积 60 天

表 B1（续）

顺序号	树种	发芽床	温度 ℃	初次计数，天	末次计数，天	备注
54	木菠萝 *Artocarpus heterophyllus* Lam.	纸	25—30	7	21	—
55	羊蹄甲 *Bauhinia purpurea* L.	纸	25	7	14	浓硫酸浸种 10 min 后充分冲洗
56	垂枝桦 *Betula pendula* Roth	纸	20—30	5	10	—
57	白　桦 *B. platyphylla* Suk.	纸	20—30	7	14	—
58	紫　珠 *Callicarpa dichotama* (lour.) K. Koch	纸	20—30	14	21	—
59	油　茶 *Camellia oleifera* Abel.	纸	25	8	12	5～10℃层积 45 天
60	茶 *C. sinensis* Kuntze	纸	25	8	12	5～10℃层积 45 天
61	喜　树 *Camptotheca acuminata* Decne.	纸	20—30	17	28	冷水浸 3 天，剥去果皮
62	柠条锦鸡儿 *Caragana korshinskii* Kom.	纸	25	9	17	—
63	小叶锦鸡儿 *C. microphylla* Lam.	纸	25	10	21	—
64	薄壳山核桃 *Carya illinoensis* (Wangenh.) K. Koch	沙	20—30	21	49	1～5℃层积 60 天
65	铁刀木 *Cassia siamea* Lam.	纸	25	10	20	浓硫酸浸种 3 min 后充分冲洗
66	锥　栗 *Castanea henryi* (Skan) Rehd. et Wils.	沙	25	7	21	—
67	板　栗 *C. mollissima* Blume	沙	20—25	12	35	0.5%$KMnO_4$ 消毒 20 min，45℃水浸种 48 h
68	红　椎 *Castanopsis hystrix* A. DC.	沙	25	7	21	—
69	青钩栲(格氏栲) *C. kawakamii* Hay.	纸	25	7	21	—
70	木麻黄 *Casuarina equisetifolia* L.	纸	30	7	14	—
71	梓　属 *Catalpa* spp.	纸	20—30	4	7	室温水浸 24 h
72	麻　楝 *Chukrasia tabularis* A. Juss.	纸	30	7	14	—
73	樟　树 *Cinnamomum camphora* (L.) Presl	纸	30	7	14	1) 15～20℃层积 30 天；<br>2) 室温水浸种 10 天，去果皮；<br>3) 19%H_2O_2 浸种 2 h
74	肉桂 *C. cassia* Presl	沙	30	7	21	—

表 B1（续）

顺序号	树种	发芽床	温度 ℃	初次计数，天	末次计数，天	备注
75	降香黄檀 *Dalbergia odorifera* T. Chen	纸	30	24	48	—
76	凤凰木 *Delonix regia* (Bojea) Raf.	沙	30	7	14	1）始温 100℃水浸种 24 h；2）浓硫酸浸种 30 min 后充分冲洗
77	君迁子 *Diospyros lotus* L.	沙	20—30	6	13	始温 45℃水浸种 24 h
78	坡柳（车桑子） *Dodonaea viscosa* (L.) Jacq.	纸	25	8	24	始温 70℃水浸种 2 h
79	沙枣 *Elaeagnus angustifolia* L.	沙	25	14	21	1）29%H_2O_2 浸种 15～20 min；2）始温 60℃水浸种 48 h
80	翅果油树 *E. mollis* Diels	纸	25	10	28	—
81	泡火绳 *Eriolaena malvacea* (Levl.) Hand. -Mzt.	纸	25	5	21	始温 70℃水浸种 24 h
82	格木 *Erythrophleum fordii* Oliv.	沙	30	7	15	1）始温 100℃水浸种 24 h；2）清除种皮上的胶状物后浓硫酸浸 30 min，充分冲洗
83	赤桉 *Eucalyptus camaldulensis* Dehnh	纸	30	7	14	称量发芽法，0.5 g
84	柠檬桉 *Eu. citriodora* Hook.	纸	25	7	14	称量发芽法，1.0 g
85	窿缘桉 *Eu. exserta* F. Muell.	纸	25	7	14	称量发芽法，1.0 g
86	蓝桉 *Eu. globulus* Labill.	纸	25	7	24	称量发芽法，1.0 g
87	葡萄桉 *Eu. botryoides* Sm.	纸	25	7	14	称量发芽法，1.0 g
88	直干蓝桉 *Eu. maidenii* F. V. M.	纸	25	7	24	称量发芽法，0.1 g
89	大叶桉 *Eu. robusta* Smith	纸	20	7	14	称量发芽法，0.25 g
90	蜡皮桉 *Eu. rubida* Decne et Maiden	纸	25，35	7	14	称量发芽法，0.25 g
91	多枝桉 *Eu. viminalis* Labill	纸	25	5	14	称量发芽法，0.5 g
92	杜仲 *Eucommia ulmoides* Oliv.	纸	25	14	21	1）划去胚根一侧，浸 24 h；2）5℃催芽 40 天
93	梧桐 *Firmiana simplex* (L.) W. F. Wight	沙	20—30	11	24	水浸 24 h 后切破种皮
94	白蜡树 *Fraxinus chinensis* Roxb.	纸	25	5	15	始温 45℃水浸种 21 天，每天换水一次
95	皂荚 *Gleditsia sinensis* Lam.	纸	20—25	12	21	1）浓硫酸浸 1 h，充分冲洗，温水浸 48 h；2）始温 45℃水浸种 24 h，余硬粒再处理 1～2 次

表 B1（续）

顺序号	树　　种	发芽床	温度 ℃	初次计数，天	末次计数，天	备　　注
96	云南石梓 *Gmelina arborea* Roxb.	纸	25—35	15	44	室温水浸种 24 h
97	银　桦 *Grevillea robusta* A. Cunn.	纸	25	14	28	始温 45℃水浸种 24 h
98	梭　梭 *Haloxylon ammodendron* (Mey.) Bunge	纸	25	3	10	—
99	白梭梭 *H. persicum* Bunge ex Boiss. et Buhse	纸	20	5	7	—
100	蒙古岩黄耆(羊柴) *Hedysarum mongolicum* Turcz.	纸	25	12	20	始温 45℃水浸种 24 h，去掉外种皮
101	花棒(细枝岩黄耆) *H. scoparium* Fisch et Mey.	纸	25	14	21	始温 45℃水浸种 24 h，去掉外种皮
102	沙　棘 *Hippophae rhamnoides* L.	纸	25	10	19	始温 45℃水浸种 24 h
103	红花天料木(母生) *Homaliun hainanense* Gagnep.	纸	30	8	27	冷水浸种后湿润 3 天；称量发芽法
104	坡　垒 *Hopea hainanensis* Merr. et Chun	纸	30	5	10	剥去翅状萼片
105	核　桃 *Juglans rigia* L.	沙	25	12	18	室温水浸 4 天，夹裂核壳，埋入沙中
106	非洲桃花心木 *Khaya senegalensis* A. Juss.	纸	20—30	10	28	室温水浸种 24 h
107	紫　薇 *Lagerstroemia indica* L.	纸	20—30	4	11	—
108	胡枝子 *Lespedeza bicolor* Turcz.	纸	20—25	3	5	去掉果皮后擦破种皮，水浸 24 h
109	枫　香 *Liquidambar formosana* Hance	纸	25	10	21	—
110	鹅掌楸 *Liriodendron chinense* (Hemsl.) Sarg.	纸	25	14	28	1～12℃层积 90 天
111	金银花(忍冬) *Lonicera japonica* Thunb.	纸	20—30	4	11	始温 45℃水浸种 72 h 后再置 1～5℃条件下层积 14 天
112	金银忍冬 *L. maackii* (Rupr.) Maxim.	纸	20—30	17	28	始温 45℃水浸 48 h
113	枸　杞 *Lycium chinense* Mill.	纸	20	7	21	—
114	绿楠(海南木莲) *Manglietia hainanensis* Dandy	纸	25	5	46	25℃层积 36 天；切开法测定优良度
115	楝　树 *Melia azedarach* L.	纸	25	10	21	剖开果核取出种子

表 B1（续）

顺序号	树　　种	发芽床	温度 ℃	初次计数,天	末次计数,天	备　　注
116	川　楝<br>*M. toosendan* Sieb. et Zucc.	纸	25	10	21	剖开果核取出种子
117	醉香含笑(火力楠)<br>*Michelia macclurei* Dandy	沙	25—30	7	21	2～17℃层积 80 天
118	桑　树<br>*Morus alba* L.	纸	30	7	21	—
119	壳菜果(米老排)<br>*Mytilaria laosensis* Lec	沙	20—30	15	30	始温 50℃水浸种 24 h
120	兰考泡桐<br>*Paulownia elongata* S. Y. Hu	纸	25—30	7	22	—
121	白花泡桐<br>*P. fortunei* (Seem). Hemsl.	纸	25—30	9	14	—
122	毛泡桐<br>*P. tomentosa* (Thunb.) Steud.	纸	25—30	7	22	—
123	黄菠萝(黄檗)<br>*Phellodendron amurense* Rupr.	纸	20—30	9	30	1) 1～5℃层积 30 天;<br>2) 染色法测定生活力
124	毛　竹<br>*Phyllostachys pubescens* Mazel ex. H. de Lehaie	纸	25	14	28	—
125	黄连木<br>*Pistacia chinensis* Bunge	沙	20—30	14	28	1～5℃层积 60 天;
126	二球悬铃木<br>*Platanus hispanica* Muenchh.	纸	20—25	5	10	—
127	杨　属<br>*Populus* spp.	纸	20—25	7	14	—
128	枫　杨<br>*Pterocarya stenoptera* C. DC.	沙	30	10	21	1～5℃层积 90 天
129	葛　藤<br>*Pueraria lobata* (Willd) Ohwi.	纸	20—30	4	7	室温水浸种 24 h,剩下的硬粒反复用 80℃水浸,每次 1～2 min,然后换常温水至 24 h
130	栎　属<br>*Quercus* spp.	沙	25	14	28	—
131	火炬树<br>*Rhus typhina* L.	纸	25—30	7	14	1) 浓硫酸浸泡 5 min,冲洗干净,再用室温水浸 48 h;<br>2) 始温 90℃水浸种 48 h
132	刺　槐<br>*Robinia pseudoacacia* L.	纸	20—30	7	14	始温 85℃～90℃水浸种 24 h,余硬粒再处理 1～2 次
133	旱　柳<br>*Salix matsudana* Koidz.	纸	20—30	7	12	—
134	乌　桕<br>*Sapium sebiferum* (L.) Roxb.	沙	25	6	21	4%NaOH 水溶液浸泡去蜡层后 1～5℃层积 30 天
135	檫　木<br>*Sassafras tsumu* (Hemsl.) Hemsl.	纸	25	14	28	—

表 B1（完）

顺序号	树 种	发芽床	温度 ℃	初次计数，天	末次计数，天	备 注
136	木 荷<br>*Schima superba* Gardn. et Champ.	纸	20—30	17	35	
137	箭 竹<br>*Sinarundinaria nitida* (Mitf.) Nakai	纸	20	9	12	—
138	槐 树<br>*Sophora japonica* L.	纸	20—25	9	14	始温 85℃～90℃水浸种 24 h，余硬粒再处理 1～2 次
139	大叶桃花心木<br>*Swietenia macrophylla* King	纸	30	7	21	室温水浸种 24 h
140	乌墨（海南蒲桃）<br>*Syzygium cumini* (L.) Skeels	纸	30	7	30	—
141	柚 木<br>*Tectona grandis* L. f.	纸	25—35	25	40	烈日下曝晒 7 天，后将种子浸沤于石灰水内 7 天，然后水浸 2 天
142	鸡尖（海南榄仁树）<br>*Terminalia hainanensis* Exell.	纸	30	20	35	露天（25℃～30℃）层积 8 天
143	香 椿<br>*Toona sinensis* (A. Juss.) Roem.	纸	25	7	21	—
144	漆 树<br>*Toxicodendron verniciflum* (Stokes) F. A. Barkley	沙	20—25	14	28	1）1～5℃层积 15～30 天；<br>2）浓硫酸浸种 30～40 min 充分洗净后 1～5℃层积 30 天
145	棕 榈<br>*Trachycarpus fortunei* (Hook. f.) H. Wendl.	纸	25	14	21	1）5～10℃层积 30 天；<br>2）染色法测定生活力；<br>3）解剖法测定优良度
146	白 榆<br>*Ulmus pumila* L.	纸	20—25	5	7	—
147	青 梅<br>*Vatica astrotricha* Hance	纸	30	5	10	剥去翅状萼片，不能浸种
148	荆 条<br>*Vitex negundo* var. *heterophylla* (Franch.) Rehd.	纸	25	5	18	—
149	大叶榉<br>*Zelkova schneideriana* Hand.-Mzt.	纸	20	14	21	1～5℃层积 40 天

注

1 备注栏提供的内容是：

a）为破除休眠建议采用的预处理方法；

b）称量种子发芽法的最低量。

2 温度栏下的"—"表示变温；

3 几种温度或几种预处理条件并列，并不表示优劣顺序。

B2 生活力的四唑和靛蓝测定技术条件见表 B2。

表 B2 生活力的四唑和靛蓝测定技术条件表

顺序号	树　种	预处理	染色前的准备	试剂种类		于 30～35℃染色		鉴定的准备	四唑鉴定不染色的最大面积；靛蓝染色的最大面积	备　注
		方式、时间<br>h		四唑	靛蓝	浓度<br>%	时间<br>h			
1	冷杉属<br>*Abies* spp.	温水浸 18	1）切开两端，打开胚腔；<br>2）在胚旁纵切	√		0.5～1.0	18～24	1）纵切胚乳，使胚露出；<br>2）除去种皮	胚乳末端有少量表面坏死	
2	杉木<br>*Cunninghamia lanceolata* (Lamb.) Hook.	温水浸 24	剥去种皮	√		0.5～1.0	4～5		无，包括胚乳	
3	银杏<br>*Ginkgo biloba* L.	温水浸 24	取“胚方”	√		0.5～1.0	15～20		1/4 胚方子叶末端	
4	刺柏属<br>*Juniperus* spp.	温水浸 24	1）从末端去 1/4；	√		0.5～1.0	4	1）纵切，使胚与胚乳露出；	无，包括子叶	
			2）在胚旁纵切	√		0.5～1.0	24	2）露出胚和胚乳	无，包括子叶	
5	松属<br>*Pinus* spp.	温水浸 18	1）从两端横切，打开胚腔；	√		0.5～1.0	18～24	1）通过胚乳纵切，使胚露出除去种皮；	无，包括胚乳	种胚短于胚腔 1/3 者，为无生活力
			2）在胚旁纵切	√		0.5～1.0	12～18	2）暴露出胚，除去种皮		
6	华山松<br>*P. armandi* Franch.	温水浸 24～48	除去种皮	√		0.5～1.0	15～20	1）纵切种子，使胚与胚乳露出；<br>2）剥出种仁	无，包括胚乳	种胚短于胚腔 1/3 者，为无生活力
7	白皮松<br>*P. bungeana* Zucc. ex Endl.	温水浸 24～48	除去种皮	√		0.5～1.0	20～24	1）纵切种子使胚与胚乳露出；<br>2）剥出种仁	无，包括胚孔	种胚短于胚腔 1/3 者，为无生活力
		温水浸 24～48	除去种皮		√	0.1	20～24	1）纵切种子使胚与胚乳露出；<br>2）剥出种仁	无，包括胚乳	种胚短于胚腔 1/3 者，为无生活力
8	湿地松<br>*P. elliottii* Engelm.	温水浸 24	除去种皮	√		0.5～1.0	2～3	1）纵切种子使胚与胚乳露出；<br>2）剥出种仁	无，包括胚乳	种胚短于胚腔 1/3 者，为无生活力
9	思茅松<br>*P. kesiya* Royle ex Gord. var. *langbianensis* (A. Chev.) Gaussen	温水浸 24	除去种皮	√		0.5～1.0	2～3	1）纵切种子使胚与胚乳露出；<br>2）剥出种仁	无，包括胚乳	种胚短于胚腔 1/3 者，为无生活力

表 B2（续）

顺序号	树　　种	预处理 方式、时间 h	染色前的准备	试剂种类 四唑	试剂种类 靛蓝	于 30～35℃染色 浓度 %	于 30～35℃染色 时间 h	鉴定的准备	四唑鉴定不染色的最大面积；靛蓝染色的最大面积	备　注
		温水浸 24	除去种皮		√	0.1	2～3	1) 纵切种子使胚与胚乳露出；2) 剥出种仁	无，包括胚乳	种胚短于胚腔 1/3 者，为无生活力
10	红松 *P. koraiensis* Sieb. et Zucc.	水浸 48	除去种皮	√		0.5～1.0	20～24	1) 纵切种子使胚与胚乳露出；2) 剥出种仁	无，包括胚乳	种胚短于胚腔 1/3 者，为无生活力
11	油松 *P. tabulaeformis* Carr.	温水浸 24	除去种皮	√		0.5～1.0	3～4	1) 纵切种子使胚与胚乳露出；2) 剥出种仁	无，包括胚乳	种胚短于胚腔 1/3 者，为无生活力
12	火炬松 *P. taeda* L.	温水浸 24	除去种皮	√		0.5～1.0	2～3	1) 纵切种子使胚与胚乳露出；2) 剥出种仁	无，包括胚乳	种胚短于胚腔 1/3 者，为无生活力
13	黄山松 *P. taiwanensis* Hayata	温水浸 24	除去种皮	√		0.5～1.0	2～3	1) 纵切种子使胚与胚乳露出；2) 剥出种仁	无，包括胚乳	种胚短于胚腔 1/3 者，为无生活力
14	云南松 *P. yunnanensis* Franch.	温水浸 24	除去种皮	√		0.5～1.0	2～3	1) 纵切种子使胚与胚乳露出；2) 剥出种仁	无，包括胚乳	种胚短于胚腔 1/3 者，为无生活力
		温水浸 24	除去种皮		√	0.1	2～3	1) 纵切种子使胚与胚乳露出；2) 剥出种仁	无，包括胚乳	种胚短于胚腔 1/3 者，为无生活力
15	侧　柏 *Platycladus orientalis* (*L.*) Franco	温水浸 24	除去种皮	√		0.5～1.0	3～4	1) 纵切种子使胚与胚乳露出；2) 剥出种仁	无，包括胚乳	种胚短于胚腔 1/3 者，为无生活力
16	圆柏 *Sabina chinensis* (L.) Ant.	温水浸 48	1) 从末端切去 1/4；2) 在胚旁纵切	√		0.5～1.0	15～20	1) 纵切使胚与胚乳露出；2) 露出胚和胚乳	无，包括子叶	
17	落羽杉 *Taxodium distichum* (L.) Rich.	温水浸 18	1) 从两端横切去 1/4，打开胚腔；2) 胚旁纵切	√		0.5～1.0	24～48	1) 纵切胚乳，使胚露出；除去种皮使胚露出；2) 除去种皮	无，包括胚乳	

表 B2（续）

顺序号	树　种	预处理 方式、时间 h	染色前的准备	试剂种类 四唑	试剂种类 靛蓝	于30～35℃染色 浓度 %	于30～35℃染色 时间 h	鉴定的准备	四唑鉴定不染色的最大面积；靛蓝染色的最大面积	备　注
18	紫杉属 *Taxus* spp.	温水浸 18	1）末端 1 mm横切（包括一小块胚乳）；2）胚旁纵切	√		0.5～1.0	24～48	纵切胚乳，使胚露出	无，包括胚乳	
19	茶条槭 *Acer ginnala* Maxim.	1）温水浸 18；2）3～5℃预冷冻 10～14 天	从果翅末端将果实切去 1/6	√		0.5～1.0	24～48	从果皮和种皮中取出胚	胚根尖端、子叶末端小面积坏死	
20	鸡爪槭 *A. palmatum* Thunb.	1）温水浸 18；2）3～5℃预冷冻 10～14 天	除两果的连接部位外，沿另三边切开果皮	√		0.5～1.0	24	从果皮和种皮中取胚	胚根尖端、子叶末端小面积坏死	
21	楹树 *Albizzia chinensis* (Osb.) Merr.	98%浓硫酸蚀 20 min 后充分冲洗，再冷水浸 24	剥去种皮	√		0.5～1.0	2～3		1/4 子叶末端	
		98%浓硫酸蚀 20 min 后充分冲洗，再冷水浸 24	剥去种皮		√	0.1	2～3		1/4 子叶末端	
22	黑格 *A. odoratissima* (L. f.) Benth.	98%浓硫酸蚀 20 min 后充分冲洗，再冷水浸 24	剥去种皮	√		0.5～1.0	2～3		1/4 子叶末端	
		98%浓硫酸蚀 20 min 后充分冲洗，再冷水浸 24	剥去种皮		√	0.1	2～3		1/4 子叶末端	
23	白格 *A. procera* (Willd.) Benth.	98%浓硫酸蚀 20 min 后冲洗，再冷水浸 24	剥去种皮	√		0.5～1.0	2～3		1/4 子叶末端	

表 B2（续）

顺序号	树　种	预处理	染色前的准备	试剂种类		于 30～35℃染色		鉴定的准备	四唑鉴定不染色的最大面积；靛蓝染色的最大面积	备　注
		方式、时间 h		四唑	靛蓝	浓度 %	时间 h			
		98%浓硫酸蚀 20 min 后充分冲洗，再冷水浸 24	剥去种皮		√	0.1	2～3		1/4 子叶末端	
24	鹅耳枥属 *Carpinus* spp.	温水浸种 18	从末端去 1/3	√		0.5～1.0	10～24	除去种皮，使胚露出	无	吸水前切割，可改进染色
25	板栗 *Castanea mollissima* Blume	温水浸 48	取"胚方"	√		0.5～1.0	3～4		1/4 胚方子叶末端	
		温水浸 48	取"胚方"		√	0.1	7～8		1/4 胚方子叶末端	
26	榛属 *Corylus* spp.	打开坚果，温水浸 18	切去子叶末端 1～2 mm，沿子叶之间劈开，不应切开成片	√		1.0<br>0.5	12～15<br>18～24	分开子叶，特别应沿不会染色部分切开	胚根尖端，子叶末端表面坏死不大于子叶腹面直径的 1/3	
27	山楂属 *Crataegus* L.	温水浸 18	从末端切去 1/3	√		0.5～1.0	10～24	取出胚	胚根尖端，1/3 子叶顶端部分，如在表面则为 1/2	
28	胡颓子属 *Elaeagnus* spp.	温水浸 18	1）沿胚边纵切；<br>2）切去两端，打开种腔	√		0.5～1.0	18～24	沿胚乳纵切露出种胚；去种皮，露出种胚	胚根尖端，1/3 子叶顶端部分，如在表面则为 1/2	
29	卫矛属 *Euonymus* spp.	温水浸 18	1）从顶端横切 1/3；<br>2）将胚乳纵切成两片，打开胚腔	√		0.5～1.0	24～48	纵切胚乳，露出种胚	无，包括胚乳	
30	白蜡树 *Fraxinus chinensis* Roxb.	除去果皮及翅，温水浸 48	避开胚中轴纵切种子，取出胚	√		0.5～1.0	30	纵切种子	1/4 顶端部分	
31	水曲柳 *F. mandshurica* Rupr.	除去果皮及翅，温水浸 48	避开胚中轴纵切种子，取出胚	√		0.5～1.0	20～30	纵切种子	1/4 顶端部分	

表 B2（续）

顺序号	树种	预处理 方式、时间 h	染色前的准备	试剂种类 四唑	试剂种类 靛蓝	于 30～35℃染色 浓度 %	于 30～35℃染色 时间 h	鉴定的准备	四唑鉴定不染色的最大面积；靛蓝染色的最大面积	备注
		除去果皮及翅，温水浸 48	避开胚中轴纵切种子，取出胚		√	0.2	20～30	纵切种子	1/4 顶端部分	
32	核桃 *Juglans regia* L.	冷水浸 48	取"胚方"	√		0.5～1.0	3～4		1/4 胚方子叶末端	
		冷水浸 48	取"胚方"		√	0.2	3～4		1/4 胚方子叶末端	
33	银合欢 *Leucaena glauca* (L.) Benth.	98%浓硫酸酸蚀 20 min 后充分冲洗，再冷水浸	剥去种皮	√		0.5～1.0	2～3		1/4 子叶末端	
34	女贞属 *Ligustrum* spp.	温水浸 18	1）从末端 1/4 横切；	√		0.5～1.0	20～24	1）纵切通过胚和胚乳；	无，包括胚乳	
			2）沿两边各纵切去一片	√		0.5～1.0	18～24	2）除去种皮		
35	鹅掌楸属 *Liriodendron* spp.	温水浸 18	1）从果翅末端对面横切一片果皮和胚乳；<br>2）纵切胚乳	√		0.5～1.0	24～48	纵切，使胚和胚乳露出	无，包括胚乳	
36	苹果属 *Malus* spp.	温水浸 18	从末端切去 1/3	√		0.5～1.0	20～24	取出种胚	胚根尖端，1/3 子叶顶端部分，如在表面则为 1/2	
37	山荆子 *M. baccata* (L.) Borkh.	温水浸 24～48	从末端切去 1/3，剥种仁	√		0.5～1.0	20	取出种胚	胚根尖端，1/3 子叶顶端部分，如在表面则为 1/2	
					√	0.2	2～4			
38	黄菠萝（黄檗） *Phellodendron amurense* Rupr.	温水浸 24	除去种皮		√	0.2	2～3	1）纵切种子使胚与胚乳露出；<br>2）剥出种仁	无，包括胚乳	
39	李属 *Prunus* spp.	打开核，取出种子，温水浸 18	除去种皮，浸水，每小时换水一次，至少浸 5 h	√		0.5～1.0	4～12	将子叶展开	胚根尖端，1/3 子叶顶端部分	

表 B2（续）

顺序号	树种	预处理	染色前的准备	试剂种类		于 30～35℃染色		鉴定的准备	四唑鉴定不染色的最大面积；靛蓝染色的最大面积	备注
		方式、时间 h		四唑	靛蓝	浓度 %	时间 h			
40	山 杏 *P. armeniaca* var. *ansu* Maxim.	打开核，取出种子，温水浸 18	除去种皮，浸水，每小时换水一次，至少浸 5 h	√		0.5～1.0	3～4	将子叶展开	胚根尖端，1/3 子叶顶端部分	
		打开核，取出种子，温水浸 18	除去种皮，浸水，每小时换水一次，至少浸 5 h		√	0.1	20	将子叶展开	胚根尖端，1/3 子叶顶端部分	
41	山 桃 *P. davidiana* (Carr.)Franch.	打开核，取出种子，温水浸 18	除去种皮，浸水，每小时换水一次，至少浸 5 h	√		0.5～1.0	3～4	将子叶展开	胚根尖端，1/3 子叶顶端部分	
		打开核，取出种子，温水浸 18	除去种皮，浸水，每小时换水一次，至少浸 5 h		√	0.1	20	将子叶展开	胚根尖端，1/3 子叶顶端部分	
42	梨 属 *Pyrus* spp.	温水浸 18	从末端切去 1/3	√		0.5～1.0	16～24	使胚露出	胚根尖端，1/3 子叶顶端部分	
43	刺 槐 *Robinia pseudoacacia* L.	用 80℃始温水浸 24	除去种皮	√		0.5～1.0	2～3		1/4 子叶末端	
		用 80℃始温水浸 24	除去种皮		√	0.2	2～3		1/4 子叶末端	
44	蔷薇属 *Rosa* spp.	温水浸 18	从末端切去 1/3	√		0.5～1.0	16～24	使胚露出	胚根尖端部分，1/3子叶顶端部分	
45	花楸属 *Sorbus* spp.	温水浸 18	从末端切去 1/3	√		0.5～1.0	18～24	使胚露出	胚根尖端部分，1/3子叶顶端部分	
46	椴 属 *Tilia* spp.	除去果皮 18	切去黑斑及一薄片胚乳	√		0.5～1.0	24～28	沿胚乳纵切，剥开外壳，轻轻挤压种子，使胚露出	无，在胚乳的表面上有微小坏死则除外	
47	鸡树条荚蒾 *Viburnum sargentii* Koehne	温水浸 48，沿纵轴切取有胚的一半染色	剥去种皮	√		0.5～1.0	6～9	取出胚	无	

表 B2（完）

顺序号	树种	预处理	染色前的准备	试剂种类		于 30～35℃染色		鉴定的准备	四唑鉴定不染色的最大面积；靛蓝染色的最大面积	备注
		方式、时间<br>h		四唑	靛蓝	浓度<br>%	时间<br>h			
48	文冠果<br>*Xanthoceras sorbifolia*	用浓硫酸浸种 3，再用温水浸 48	除去种皮	✓		0.5～1.0	8～10		1/4 子叶末端	
	Bunge	用浓硫酸浸种 3，再用温水浸 48	除去种皮		✓	0.1	8～10		1/4 子叶末端	

B3 四唑和靛蓝染色示意图见表 B3、表 B4。

表 B3 四唑和靛蓝染色示意图（四唑染色鉴定）

序号	树种及其种子染色示意图	说明
1	银杏（取胚方）<br>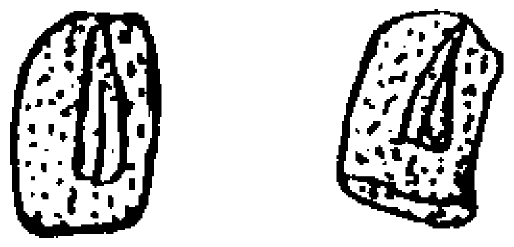	有生活力者：<br>胚、胚乳均染色或胚乳少部分未染色。
2	松属、杉木属、侧柏属<br>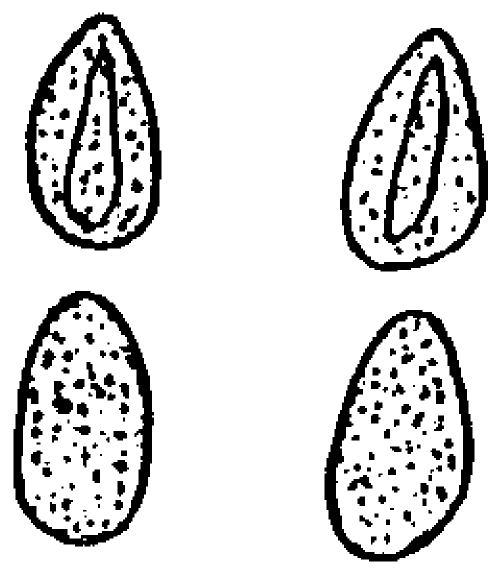	有生活力者：<br>胚乳、胚全部染色，仅有少部分胚乳染色较淡。
3	台湾相思、肯氏相思、黑荆、孔雀豆、南洋楹、新银合欢<br>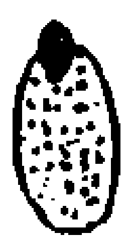<br>a)<br>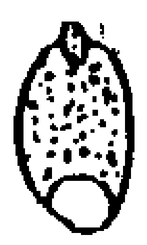<br>b)	有生活力者：<br>a）胚根、胚轴和子叶完全染色；<br>b）胚根、胚轴染色，子叶大部分染色。
4	元宝枫<br>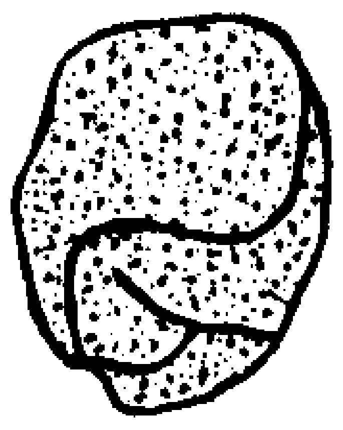<br>a)<br>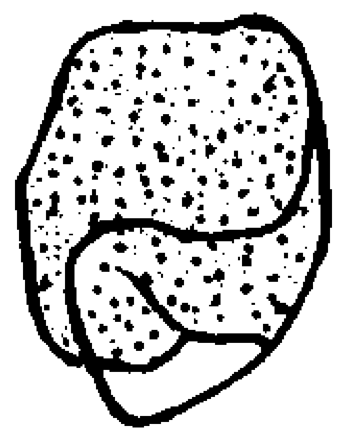<br>b)	有生活力者：<br>a）胚全部染色；<br>b）胚根染色，子叶大部分染色。

表 B3（续）

序号	树种及其种子染色示意图	说　　明
5	香椿、臭椿、黄连木<br>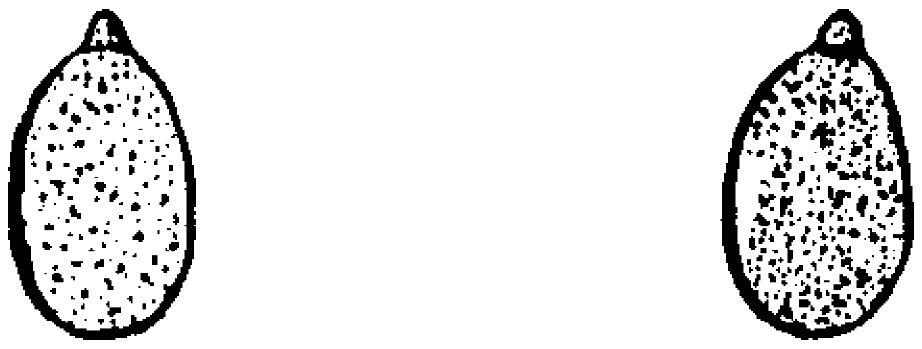<br>a)　　　　b)	有生活力者：<br>a）胚根、下胚轴、子叶全部染色；<br>b）胚根、下胚轴染色，子叶大部分染色。
6	板栗、锥栗（取胚方）<br>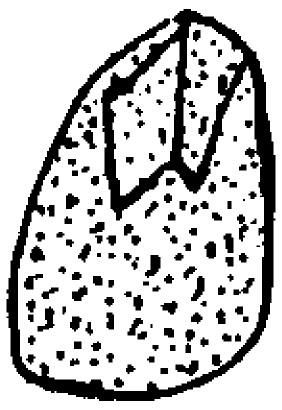	有生活力者：<br>胚根、下胚轴和子叶完全染色，或子叶少部分未染色。
7	沙枣<br>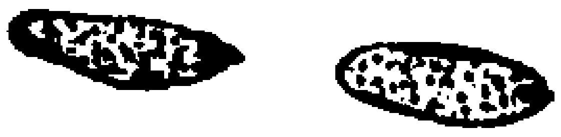	有生活力者：<br>胚根、下胚轴、子叶均染色，或子叶少部分未染色。
8	水曲柳、白蜡<br>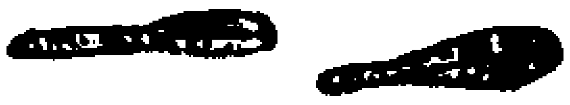	有生活力者：<br>胚根、胚轴、子叶均染色，或子叶少部分未染色。
9	核桃（取胚方）<br>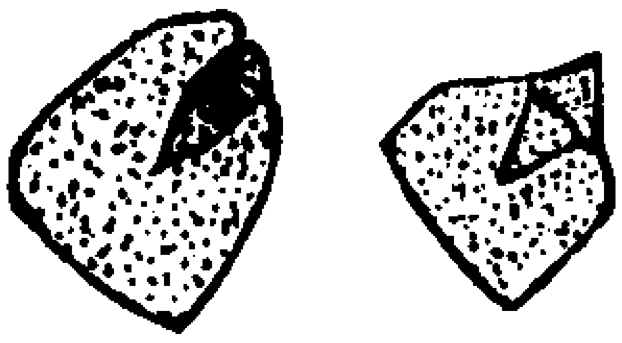	有生活力者：<br>胚根、下胚轴、子叶均染色，或子叶少部分未染色。
10	枸杞<br>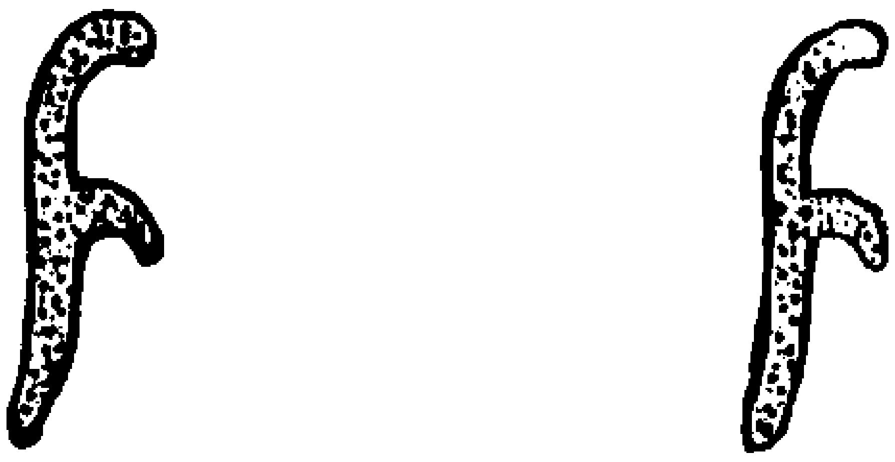<br>a)　　　　b)	有生活力者：<br>a）胚全部染色；<br>b）胚根染色，子叶少许未染色。
11	苦楝、川楝<br>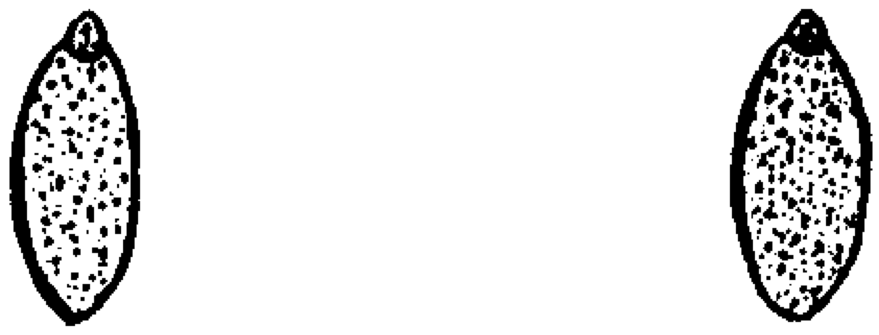<br>a)　　　　b)	有生活力者：<br>a），b）胚根染色，子叶全部或大部分染色。

表 B3（续）

序号	树种及其种子染色示意图	说明
12	壳莱果（米老排）<br>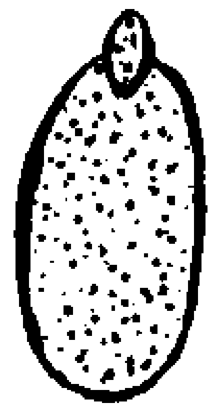	有生活力者：<br>胚全部染色，子叶全部染色或大部分染色。
13	黄菠萝（黄檗）<br>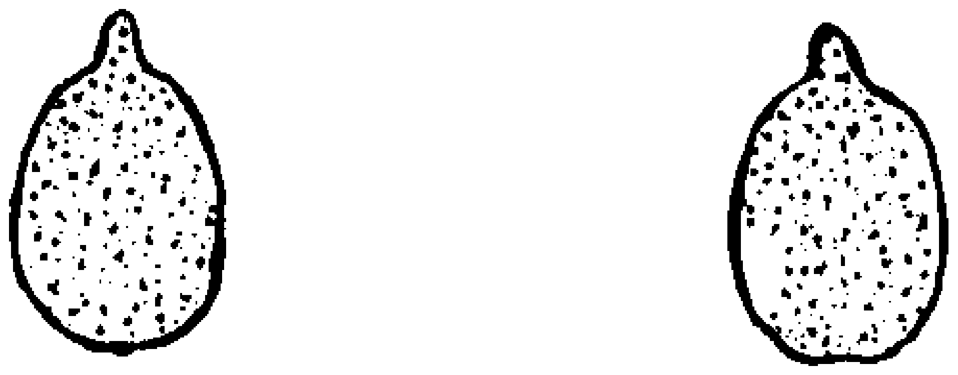<br>a)　　　　b)	有生活力者：<br>a）胚全部染色；<br>b）胚根染色，子叶大部分染色。
14	山桃、山杏<br>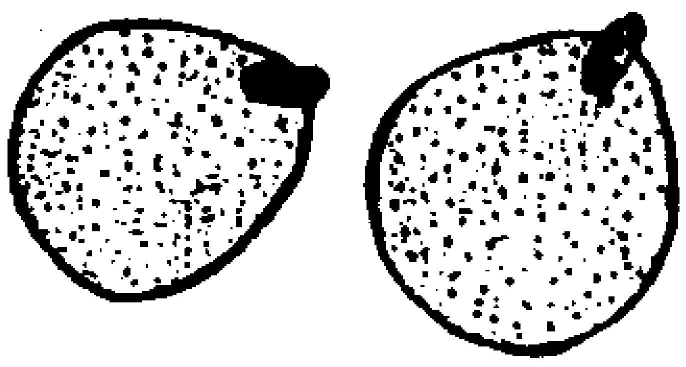	有生活力者：<br>胚根、胚轴、子叶均染色，或子叶少部分未染色。
15	刺槐、槐树、皂角、凤凰木、铁刀木<br>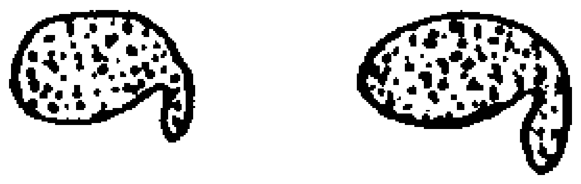<br>a)<br>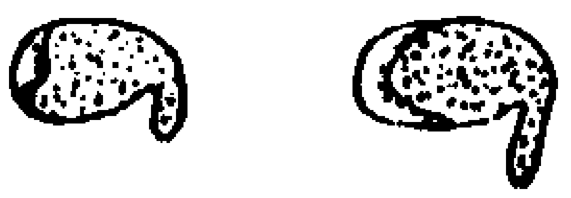<br>b)	有生活力者：<br>a）胚根、下胚轴、子叶均染色；<br>b）胚根、下胚轴全部染色，子叶少部分未染色或部分染色较淡。
16	安息香<br>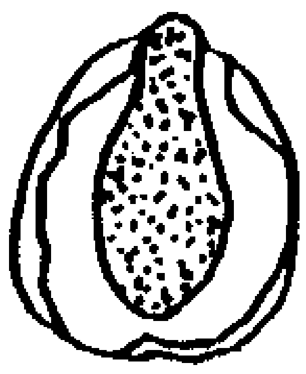	有生活力者：<br>胚根、子叶全部染色。
17	椴树<br>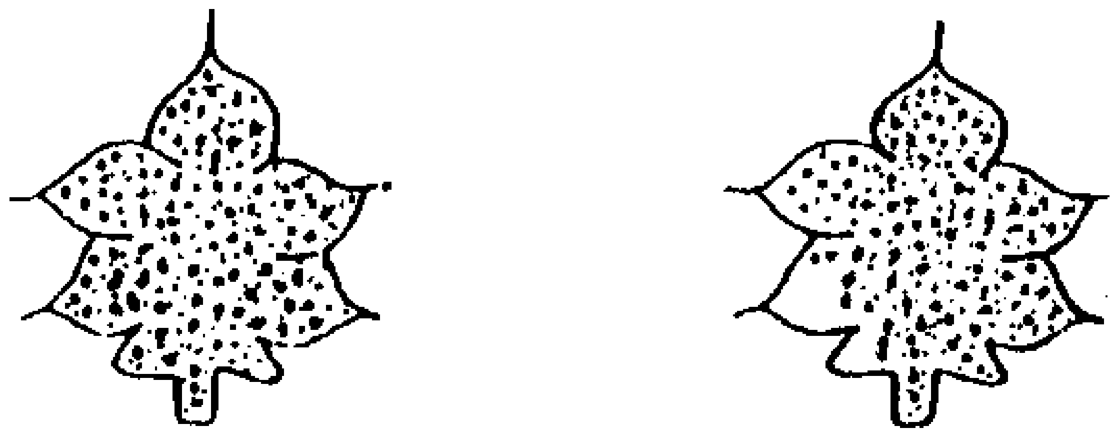<br>a)　　　　b)	有生活力者：<br>a）胚根、子叶全部染色；<br>b）胚根染色，子叶大部分染色。

表 B3（完）

序号	树种及其种子染色示意图	说　　明
18	漆树<br>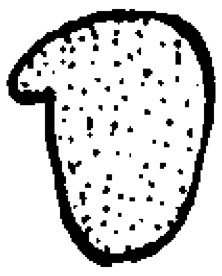	有生活力者：<br>胚完全染色，胚根、胚轴染色，子叶完全染色或少部分未染色。
19	棕榈<br>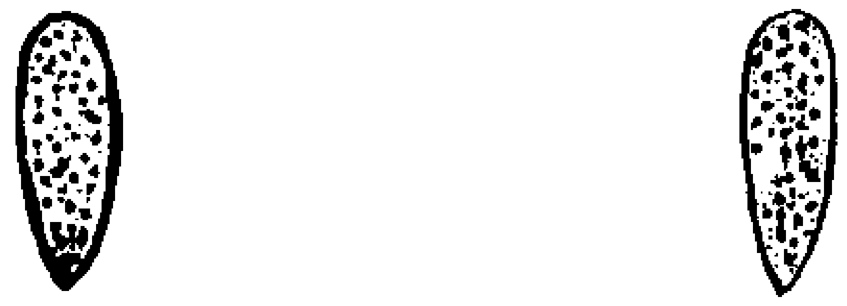<br>a)　　b)	有生活力者：<br>a) 胚全部染色；<br>b) 胚中部边缘少部分未染色。
20	文冠果<br>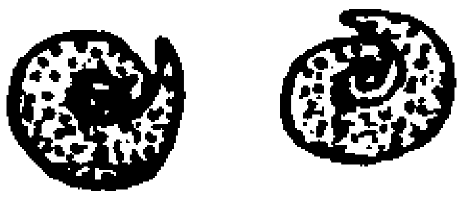	有生活力者：<br>胚根、子叶均染色，或子叶少部分未染色。

表 B4　四唑和靛蓝染色示意图(靛蓝染色鉴定)

序号	树种及其种子染色示意图	说　　明
1	松属、杉木属<br>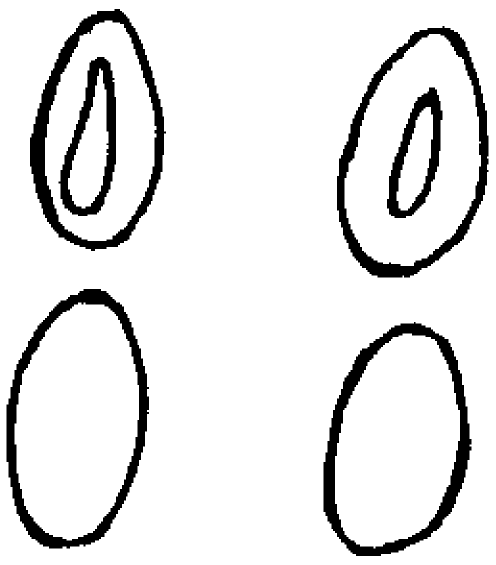	有生活力者：<br>胚乳、胚全部未染色，或仅有少部分胚乳染色较淡。
2	元宝枫<br>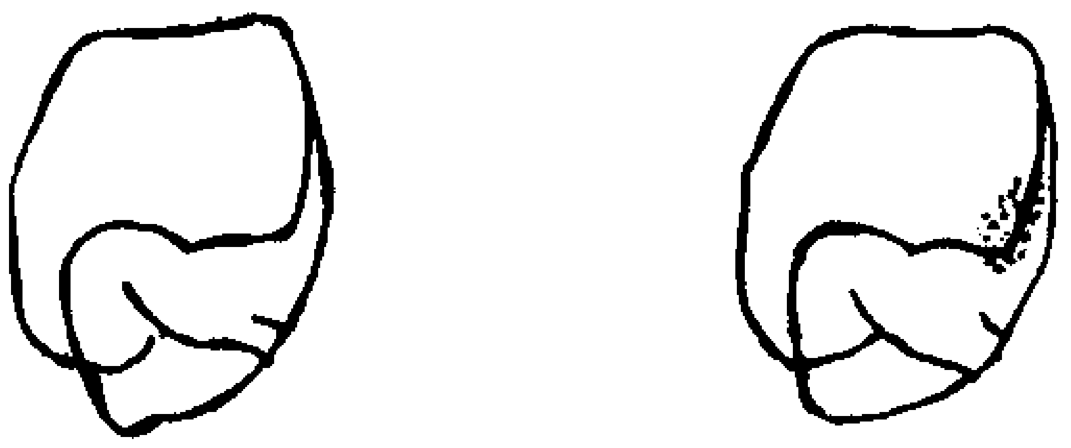<br>a)　　b)	有生活力者：<br>a) 胚全部未染色；<br>b) 子叶少许染色。
3	香椿、臭椿、黄连木<br>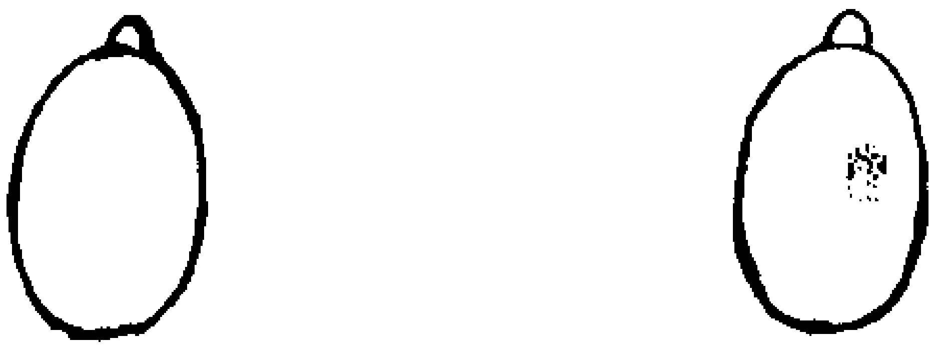<br>a)　　b)	有生活力者：<br>a) 胚全部未染色；<br>b) 子叶少许染色。

表 B4（续）

序号	树种及其种子染色示意图	说　　明
4	沙枣<br>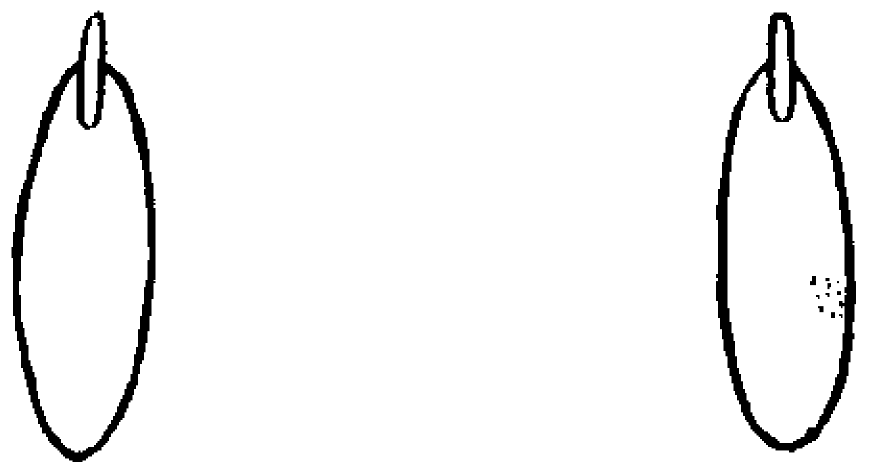<br>a)　　b)	有生活力者：<br>a）胚全部未染色；<br>b）子叶少许染色。
5	水曲柳、白蜡<br>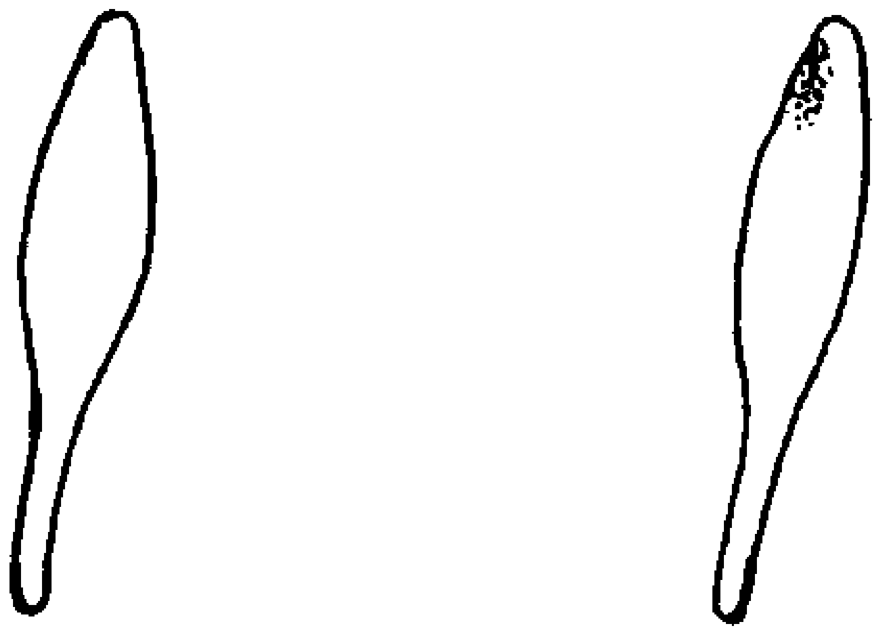<br>a)　　b)	有生活力者：<br>a）胚全部未染色；<br>b）子叶少许染色。
6	枸杞<br>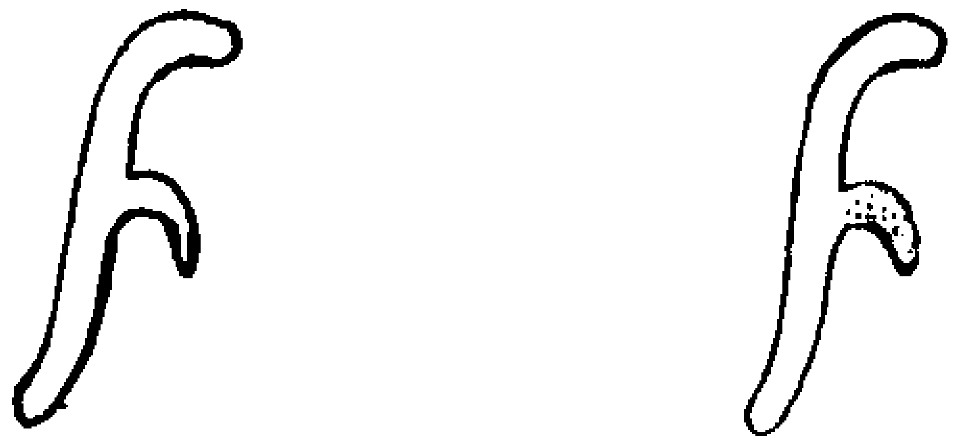<br>a)　　b)	有生活力者：<br>a）胚全部未染色；<br>b）子叶少许染色。
7	苦楝、川楝<br>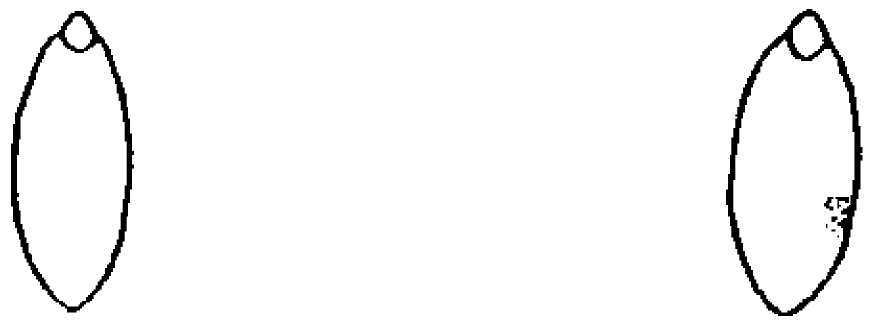<br>a)　　b)	有生活力者：<br>a）胚全部未染色；<br>b）子叶少许染色。
8	壳莱果（米老排）<br>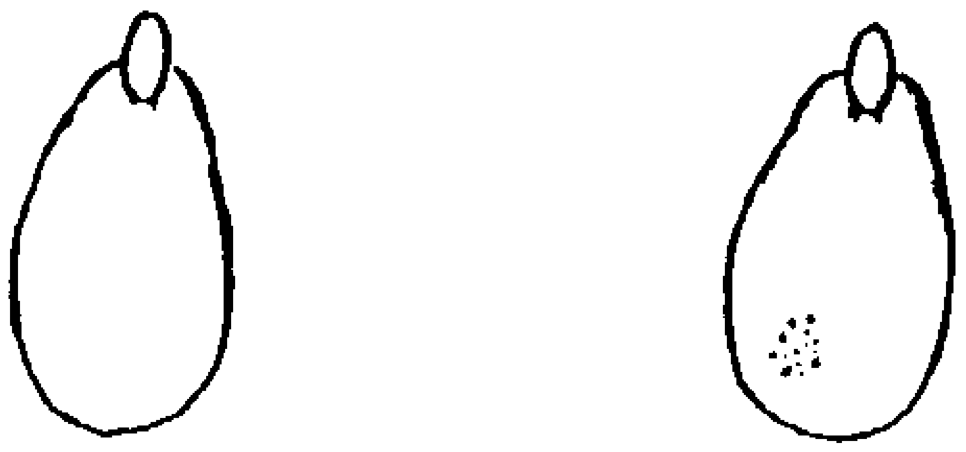<br>a)　　b)	有生活力者：<br>a）胚全部未染色；<br>b）子叶少许染色。

表 B4（完）

序号	树种及其种子染色示意图	说　　明
9	黄菠萝（黄檗）<br>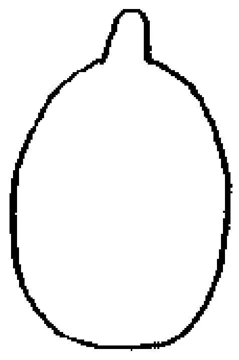 a）<br>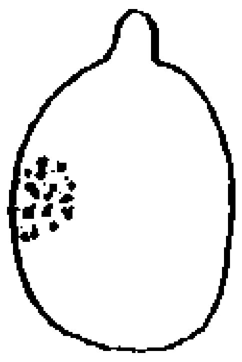 b）	有生活力者：<br>a）胚全部未染色；<br>b）子叶少许染色。
10	刺槐、槐树、皂角、凤凰树、铁刀木<br>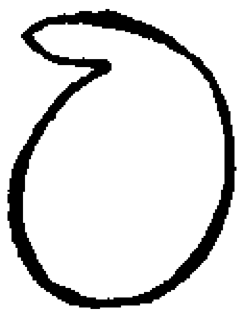 a）<br>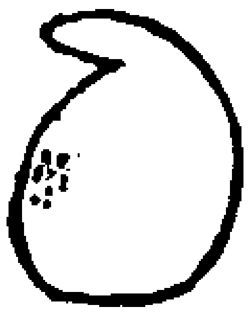 b）	有生活力者：<br>a）胚全部未染色；<br>b）子叶少许染色。
11	椴树<br>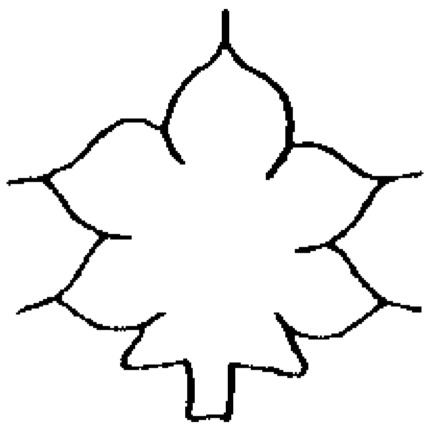 a）<br>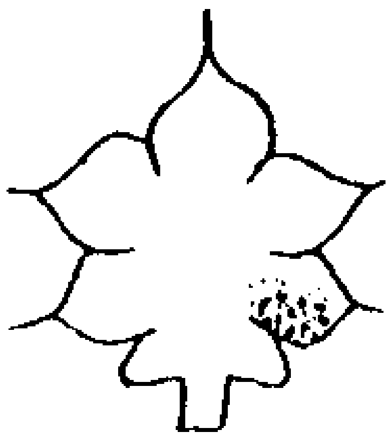 b）	有生活力者：<br>a）胚全部未染色；<br>b）子叶少许染色。
12	棕榈<br>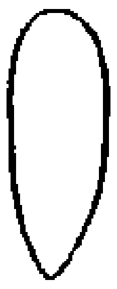 a）<br>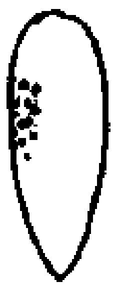 b）	有生活力者：<br>a）胚全部未染色；<br>b）胚少许染色。
13	文冠果<br>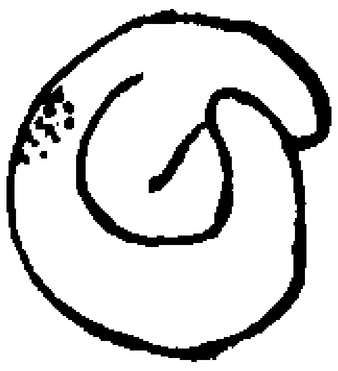 a）<br>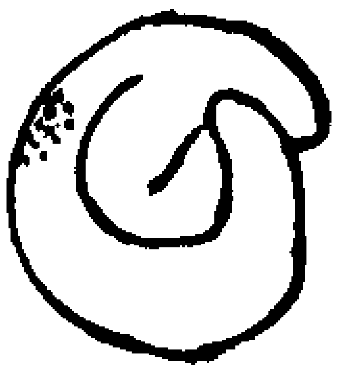 b）	有生活力者：<br>a）胚全部未染色；<br>b）子叶少许染色。

B4　优良种子鉴别见表 B5。

表 B5 优良种子鉴别表

顺序号	树种	优良种子
1	岷江冷杉 *Abies faxoniana* Rehd. et Wils.	种皮表面黑褐色,有光泽,富含松脂香味;胚乳乳白色,饱满,胚根淡清白色,子叶淡清绿色,新鲜
2	杉松(沙松) *A. holophylla* Maxim.	种粒饱满,胚乳、胚白色
3	三尖杉 *Cephalotaxus fortunei* Hook. f.	胚乳饱满,白色或淡黄色
4	柳 杉 *Cryptomeria fortunei* Hooibrenk	种子饱满;胚呈暗白色
5	杉木 *Cunninghamia lanceolala* (Lamb.) Hook.	种皮赤褐色,有光泽;种仁饱满,胚完好,胚根稍带粉红色,胚尖淡红色,胚乳白色、淡白色
6	干香柏 *Cupressus duclouxiana* Hickel	胚乳外被黄褐色,内呈乳白色,先端褐黄色,胚白色,饱满,新鲜
7	柏 木 *C. funebris* Endl.	种仁黄白色,先端黄褐色,中部淡褐色,基部棕色
8	银 杏 *Ginkgo biloba* L.	胚乳饱满,表面浅黄色,切开后胚乳黄绿色,胚浅黄绿色
9	杜 松 *Juniperus rigida* Sieb. et Zucc.	种皮棕白色;种仁饱满,胚、胚乳呈白色
10	落叶松(兴安落叶松) *Larix gmelinii* (Rupr.) Rupr.	种子腹面褐色,背面浅褐色,有光泽,胚、胚乳乳白色,饱满,有弹性
11	华北落叶松 *L. principis-rupprechtii* Mayr	种皮腹面褐色,背面黄白色,有光泽;种仁饱满,胚乳尖端部分呈乳色,钝部白色,胚乳白色,有松脂香味,种仁切面平滑;浸种后种仁膨胀,质硬脆,胚乳乳白色,胚靠根尖部呈浅黄或淡黄绿色,其靠子叶部呈鲜白色;用84%的酒精浸种可快速测出饱满度
12	白 杄 *Picea meyeri* Rehd. et Wils.	种皮黑褐色或灰褐色,种粒饱满;胚、胚乳皆白色,有松脂香味;浸种后种仁膨胀,色鲜,质硬脆
13	青 杄 *P. wilsonii* Mast.	种皮黑褐色或灰褐色,种粒饱满;胚、胚乳皆白色,有松脂香味;浸种后种仁膨胀,色鲜,质硬脆
14	华山松 *Pinus armandi* Franch.	种仁饱满,有松脂香味,胚乳乳白色,胚白色,其两端呈微淡黄色或微淡黄绿色;浸种后种仁膨胀,胚乳白色,有鲜嫩感
15	白皮松 *P. bungeana* Zucc. ex Endl.	种仁饱满,有松脂香味,胚乳乳白色,胚白色,其两端呈微淡黄色或微淡黄绿色;浸种后种仁膨胀,胚乳白色,有鲜嫩感,胚根部微黄色或黄绿色,质硬脆
16	赤 松 *P. densiflora* Sieb. et Zucc.	种皮赤褐色,胚、胚乳皆白色,有松脂香味,有弹性
17	红 松 *P. koraiensis* Sieb. et Zucc.	种粒饱满,浅红棕色;胚、胚乳乳白色,饱满,有弹性,富松脂香味
18	马尾松 *P. massoniana* Lamb.	种皮黑褐、灰褐、灰棕或黄白色;温水浸种 20~24 h,胚乳白色,胚黄或红色

表 B5（续）

顺序号	树　　种	优　良　种　子
19	樟子松 *P. sylvestris* L. var. *mongolica* Litv.	种粒饱满，胚、胚乳白色，有弹性，具松脂香味
20	油　松 *P. tabulaeformis* Carr.	种皮黑褐色或灰褐色，有光泽；种仁饱满，胚乳白色，胚白色，其靠根部具微浅黄色，有松脂香味；浸种后种仁膨胀，质硬脆，胚乳、胚鲜白色，胚靠根尖部呈鲜淡黄色或鲜淡黄绿色
21	黄山松 *P. tewwanensis* Hayata	种粒饱满，种皮黑褐色或灰褐色；胚、胚乳白色，有松脂香味
22	黑　松 *P. thunbergii* Parl.	种粒饱满，种皮黑褐色或灰褐色；胚、胚乳白色，有松脂香味
23	云南松 *P. yunnanensis* Franch.	胚乳乳白色，胚白色，饱满，新鲜
24	侧柏 *Platycladus orientalis* (L.) Franco	种粒饱满，种皮棕褐色，有光泽；胚白色，胚乳乳白色或黄白色；浸种后种仁膨胀，色鲜，质硬脆
25	罗汉松 *Podocarpus macrophyllus* (Thunb.) D. Don	胚乳白色，胚黄绿色或淡黄绿色
26	竹　柏 *P. nagi* (Thunb.) zoll. et Mor.	胚乳白色，胚黄绿色或淡黄绿色
27	圆　柏 *Sabinia chinensis* (L.) Ant.	胚、胚乳白色，饱满，较软
28	南方红豆杉 *Taxus mairei* (Lemee et Levl.) S. Y. Hu ex Lin	种粒饱满，种皮棕色，有光泽；胚、胚乳皆白色
29	儿茶 *Acacia catechu* Willd.	种子暗绿色，扁平，饱满，稍有光泽；子叶淡黄色，光滑，较硬，有弹性，远离胚根有少量白色凹陷部分种仁带青色
30	青榨槭 *Acer davidi* Franch.	翅果橙棕色，饱满；子叶黄色或浅黄绿色，胚根较白
31	茶条槭 *A. ginnala* Maxim.	翅果橙棕色，饱满；子叶黄色或浅黄绿色，胚根较白
32	五角枫 *A. mono* Maxim.	翅果褐色，饱满；种仁嫩绿，饱满，有弹性
33	地锦槭 *A. pictum* thunb.	翅果嫩绿，饱满；种仁嫩绿，饱满，有弹性
34	元宝枫 *A. truncatum* Bunge	翅果橙棕色，饱满；子叶黄色或浅黄绿色，胚根白色
35	海红豆 *Adenanthera pavonina* L.	种子鲜红色，有光泽；内种皮透明，无色，凝胶状，有弹性；胚淡黄色
36	七叶树 *Aesculus chinensis* Bunge	胚浅黄色，湿润膨大，有油脂

表 B5（续）

顺序号	树　种	优　良　种　子
37	臭　椿 *Ailanthus altissima* (Mill.) Swingle	翅果褐色，饱满，种皮黄白色，种仁浅黄色
38	楹　树 *Albizia chinensis* (Osb.) Merr.	种子青褐色；子叶淡黄色，较厚
39	合　欢 *A. julibrissin* Durazz.	种子褐色，饱满，有光泽；子叶黄色不透明
40	油　桐 *Aleurites fordii* Hemsl.	内种皮纸质，粉白色，种仁饱满，有弹性，胚乳光滑，黄白色，子叶白色
41	赤　杨 *Alnus japonica* (Thunb.) Steud.	种粒饱满，子叶白色，种仁乳白色，饱满
42	辽东桤木（水冬瓜） *A. sibirica* Fisch.	种仁乳白色，饱满
43	紫穗槐 *Amorpha fruticosa* L.	荚赤褐色；种皮棕色或浅灰绿色；种仁鲜黄绿色，子叶和胚根均为淡黄色
44	白花羊蹄甲 *Bauhinia acuminata* L.	种子黄色，饱满，有光泽；子叶黄白色
45	羊蹄甲 *B. purpurea* L.	内种皮透明；子叶黄色；较肥大，有皱纹
46	黄芦木 *Berberis amurensis* Rupr.	胚稍具黄绿色，胚乳乳白色，饱满而硬，切面光滑
47	重阳木 *Bischofia javanica* Bl.	种子棕色或棕褐色，饱满；胚、胚乳均白色
48	油　茶 *Camellia olifera* Abel.	内种皮紧贴子叶，子叶肥厚，乳黄色，饱满，有弹性
49	喜　树 *Camptotheca acuminata* Decne.	胚淡绿色，胚乳白色
50	柠条锦鸡儿 *Caragana korshinskii* Kom.	种皮黄褐或栗褐色，光滑，子叶米黄色，种粒饱满均匀
51	小叶锦鸡儿 *C. microphylla* Lam.	种粒饱满，种皮棕褐色或灰褐色，子叶淡黄色
52	山核桃 *Carya cathayensis* Sarg.	子叶饱满，乳白色，有油香味
53	薄壳山核桃 *C. illinoensis* (Wangenh.) K. Koch	子叶饱满，乳白色，有油香味
54	铁刀木 *Cassia siamea* Lam.	种子红黑色，有光泽；子叶黄绿色，胚根白色，有弹性
55	板　栗 *Castanea mollissima* Blume	种壳栗褐色至浓褐色，饱满，坚硬，表面光洁；子叶浅黄色，较硬，有弹性及清香味，内外均无异状，胚芽键全，无黑点；子叶上虽有暗棕色条纹，但面积不超过 1/4
56	梓　树 *Catalpa ovata* Don	种子灰色或灰棕色，饱满；子叶白色

表 B5（续）

顺序号	树　　种	优良种子
57	南蛇藤<br>*Celastrus orbiculatus* Thunb.	胚浅白绿色，硬，胚乳粉白色
58	麻　楝<br>*Chukrasia tabularis* A. Juss.	胚、胚乳均白色，无病虫
59	樟　树<br>*Cinnamomum camphora* (L.) Presl	胚、胚乳均白色，具樟油香味，油分多
60	灯台树<br>*Cornus controversa* Hemsl.	胚淡黄色，饱满
61	毛　梾<br>*C. walteri* Wanger.	胚、胚乳均白色
62	山　楂<br>*Crataegus pinnatifida* Bunge	子叶乳白色，饱满，胚根白色
63	青　冈<br>*Cyclobalanopsis glauca* (Thunb.) Oerst.	种子暗褐色，具淡黄色绒毛，坚硬；子叶较硬，有弹性，浅灰黄色，种仁接触空气即呈深暗色
64	黄　檀<br>*Dalbergia hupeana* Hance	种子黄褐色，饱满，有光泽；子叶淡黄色
65	降香黄檀<br>*D. odorifera* T. Chen	种子红褐色，薄，饱满，有光泽；子叶鲜红色
66	凤凰木<br>*Delonix regia* (Bojea.) Raf.	胚淡黄色，胚乳灰白，坚硬，饱满
67	君迁子<br>*Diospyros lotus* L.	种粒坚硬较厚；胚乳灰白色坚硬，胚白色
68	猫尾树<br>*Dolichandrone caunda-felina* (Hance) Benth. et Hook. f.	子叶、胚根、胚芽均白色，健壮，或子叶离开胚根部的边缘带有黑色斑点，子叶较硬，有弹性
69	沙　枣<br>*Elaeagnus angustifolia* L.	种粒饱满，肉质；子叶白色，有光泽，剖面淡黄色或近于白色
70	青皮象耳豆<br>*Enterolobium contortisiliquum* Morong	种子黑红色，饱满，有光泽；子叶淡黄色
71	杜　仲<br>*Eucommia ulmoides* Oliv.	种子淡栗色，饱满，有光泽；胚乳完整，有弹性，胚白色
72	卫矛<br>*Euonymus alatus* (Thunb.) sieb.	胚完整，新鲜，浅黄色或黄绿色，胚乳白色
73	水青冈<br>*Fagus longipetiolata* Seem.	子叶、胚根均白色，饱满，有光泽
74	梧　桐<br>*Firmiana simplex* (L.) W. F. Wight	种粒饱满，略有香味；胚乳白色，新鲜，无味或有香味，胚黄色
75	连　翘<br>*Forsythia suspensa* (Thunb.) Vahl	种皮棕黄、微红，种仁白色，饱满

表 B5（续）

顺序号	树 种	优 良 种 子
76	美国白蜡树<br>*Fraxinus americana* L.	胚白色或淡白色，有弹性，胚乳乳白色或淡蓝色，较硬
77	水曲柳<br>*F. mandshurica* Rupr.	胚白色或淡白色，有弹性，胚乳乳白色或淡蓝色，较硬
78	花曲柳<br>*F. rhynchophylla* Hance	胚白色或淡白色，有弹性，胚乳乳白色或淡蓝色，较硬
79	皂 荚<br>*Gleditsia sinensis* Lam.	种子黄褐色；胚根、子叶浅黄色，饱满，子叶多开展
80	蒙古岩黄蓍（羊柴）<br>*Hedysarum mongolicum* Turcz.	种粒饱满，种皮淡黄色；子叶较硬，黄色
81	花棒（细枝岩黄蓍）<br>*H. scoparium* Fisch. et Mey.	种皮黄色，皮上有少量绒毛，种粒饱满；子叶较硬，黄色
82	沙 棘<br>*Hippophae rhamnoides* L.	胚根浅黄色，子叶乳白色，饱满，有弹性
83	冬 青<br>*Ilex purpurea* Hassk.	种子深褐色，饱满；胚乳乳白色，饱满
84	核桃楸<br>*Juglans mandshurica* Maxim.	内种皮淡黄色，有光泽；子叶淡黄白色，饱满，有弹性，具香味
85	核 桃<br>*J. regia* L.	内种皮淡黄色，有光泽；子叶饱满，淡黄白色，有油香味
86	栾 树<br>*Koelreuteria paniculata* Laxm.	子叶淡黄色；偶带绿色，饱满，有弹性
87	胡枝子<br>*Lespedeza bicolor* Turcz.	荚褐色，饱满；种仁黄白色，饱满
88	女 贞<br>*Ligustrum lucidum* Ait.	种粒饱满；胚、胚乳均白色
89	枸 杞<br>*Lycium chinense* Mill.	种粒饱满，胚、胚乳均白色
90	仪 花<br>*Lysidice rhodostegia* Hance	浸种 2 日后，种皮变软，切开后子叶灰白色，无斑点
91	玉 兰<br>*Magnolia denudata* Desr.	种仁饱满，与壳同大，有油质，种仁尖端呈黄色，胚乳黄色，胚白色，油分多
92	厚 朴<br>*M. officinalis* Rehd. et wils.	种仁饱满，与壳同大，有油质，种仁尖端呈黄色，胚乳黄色，胚白色，油分多
93	天女花<br>*M. sieboldii* K. Koch	种子棕色或棕黄色，饱满；胚乳表面黄色，切开呈清白色，油分多
94	山荆子<br>*Malus baccata* (L.) Borkh.	种皮有光泽；子叶、胚白色，饱满；浸种后种仁白色，有新鲜感，质硬脆
95	西府海棠<br>*M. micromalus* Makino	种皮有光泽；子叶、胚白色，饱满；浸种后种仁白色，有新鲜感，质硬脆
96	楝 树<br>*Melia azedarach* L.	种粒饱满；胚根淡黄色，子叶白色，有光泽
97	川 楝<br>*M. toosendan* sieb. et Zucc.	种粒饱满；胚根淡黄色，子叶白色，有光泽

表 B5（续）

顺序号	树　　种	优　良　种　子
98	壳莱果(米老排)<br>*Mytilaria laosensis* Lec.	种仁白色，有带苦的油香味
99	蓝果树<br>*Nyssa sinensis* Oliv.	胚、胚乳均白色，完整
100	木蝴蝶(千张纸)<br>*Oroxylum indicum* (L.) Vent.	子叶黄色或淡黄色
101	银　珠<br>*Peltophorum tonkinense* (pierre) Gagnep.	种子扁平，光滑；内种皮无色，透明，有弹性；子叶、胚根淡黄色
102	黄菠萝(黄檗)<br>*Phellodendron amurense* Rupr.	种子黑褐色，饱满，有光泽；胚、胚乳均白色，有弹性，或胚淡黄色
103	桢　楠<br>*Phoebe zhennan* S. Lee et F. N. wei	种仁饱满，胚、胚乳皆白色
104	紫　楠<br>*Ph. sheareri* (Hemst.) Gamble	种子黑褐色或灰黄色，有灰、黑条纹，有光泽；子叶淡黄色，较硬，有弹性
105	石　楠<br>*Photinia serrulata* Lindl.	胚白色
106	黄连木<br>*Pistacia chinensis* Bunge.	子叶淡黄色或淡黄绿色，胚根白色
107	小叶杨<br>*Populus simonii* Carr.	种粒饱满，子叶白色
108	山　杏<br>*Prunus armeniaca* var. *ansu* Maxim.	子叶乳白色，饱满，坚硬，胚根比子叶色白
109	山　桃<br>*P. davidiana* (Carr.) Franch.	子叶乳白色，饱满，较硬，有弹性
110	紫　檀<br>*Pterocarpus indicus* Willd.	种子灰黄色，有光泽；子叶米黄色，饱满
111	枫　杨<br>*Pterocarya stenoptera* C. DC.	种子深褐色；种仁乳白色，饱满
112	翻白叶树<br>*Pterospermum heterophyllum* Hance	胚根、子叶白色
113	杜　梨<br>*Pyrus betulaefolia* Bunge	子叶乳白色，饱满，坚硬，有弹性，胚根白色
114	麻　栎<br>*Quercus acutissima* Carr.	种粒饱满，棕黄色，有光泽；子叶硬，有弹性，浅黄白色或带红色，胚芽、胚根正常，无虫害
115	两广梭罗树<br>*Reevesia thyrsoidea* Lindl.	种粒饱满，胚乳肥厚，胚白色或黄白色，子叶叶脉明显

表 B5（完）

顺序号	树　　种	优 良 种 子
116	刺　槐<br>*Robinia pseudoacacia* L.	种粒饱满，种皮黑褐色或棕褐色，有光泽；子叶、胚根均为淡黄色，发育正常
117	乌　桕<br>*Sapium sebiferum* (L.) Roxb.	胚乳、胚根、子叶均为白色，新鲜，有弹性
118	檫　木<br>*Sassafras tsumu* (Hemsl.) Hemsl.	手捏硬、饱满；子叶饱满新鲜，剖面中部颜色较深，带油光，最外圈色淡，呈淡白色或绿色
119	木　荷<br>*Schima superba* Gardn. et Champ.	胚和胚乳白色
120	硬　核<br>*Scleropyrum wallichianum* (Wight et Arn.) Arn.	胚和胚乳白色
121	仿　栗<br>*Sloanea hemsleyana* (Ito) Rehd. et Wils.	种壳褐色，有光泽；子叶淡黄色，饱满
122	槐　树<br>*Sophora japonica* L.	种粒饱满，子叶浅绿色，胚根黄色
123	紫丁香<br>*Syringa oblata* Lindl.	种粒饱满，子叶浅绿色，胚根黄色
124	厚皮香<br>*Ternstroemia gymnanthera* (Wight et Arn) Sprague	胚白色
125	紫　椴<br>*Tilia amurensis* Rupr.	种粒饱满，种皮深褐色；种仁淡黄白色，有弹性
126	糠　椴<br>*T. mandschurica* Rupr. et Maxim.	种粒饱满，干种解剖时，胚黄色，胚乳黄白色；浸种后解剖，胚淡黄色，胚乳白色，子叶舒展
127	香　椿<br>*Toona sinensis* (A. Juss.) Roem.	种粒饱满，种仁淡黄白色
128	棕　榈<br>*Trachycarpus fortunei* (Hook. f.) H. Wendl.	果皮蓝黑色，种皮黑褐色，饱满；胚乳白色，透明，胚黄白色
129	石笔木<br>*Tutcheria spectabilis* Dunn	胚根黄白色，新鲜，饱满
130	文冠果<br>*Xanthoceras sorbifolia* Bunge	种粒饱满，有光泽；胚白色

附 录 C
（标准的附录）
测定原始记录表

C1 检验申请见表 C1。

表 C1 检验申请表

编号__________

____

现有送检样品一份，简要情况如下，请给予检验。

一、树种名称______________________

二、采种地点______________________

三、采种时间______________________

四、送检样品重(g)______________________

五、种批编号______________________

六、本批种子重量(kg)__________ 容器件数__________

七、要求检验项目______________________

____

八、质量检验证书寄往地点和单位名称______________________

（附：林木采种登记表）

送检单位（盖章）

扦样人__________

联系人__________

日 期__________

C2 净度分析记录见表 C2。

表 C2 净度分析记录表

编号__________

树种__________ 样品号__________ 样品情况__________

测试地点______________________

环境条件：室内温度__________℃ 湿度__________%

测试仪器：名称__________ 编号__________

方 法	试样重 g	纯净种子重 g	其他植物种子重 g	夹杂物重 g	总 重 g	净 度 %	备 注
实际差距				容许差距			

本次测定：有效 □

无效 □

测定人__________

校核人__________

测定日期______年______月______日

C3 发芽测定记录见表C3。

表C3 发芽测定记录表

编号＿＿＿＿

树种＿＿＿＿ 样品号＿＿＿＿ 样品情况＿＿＿＿ 测试地点＿＿＿＿

环境条件:室内温度＿＿＿＿℃ 湿度＿＿＿＿% 测试仪器:名称＿＿＿＿ 编号＿＿＿＿

预处理＿＿＿＿ 置床日期＿＿＿＿ 测定条件＿＿＿＿

项目		正常幼苗数								不正常幼苗数	未萌发粒分析							
项目		样品重 g	初次计数					末次计数	合计		新鲜粒	死亡粒	硬粒	空粒	无胚粒	涩粒	虫害粒	合计
日期																		
重复	1																	
	2																	
	3																	
	4																	
平均																		

组间最大差距＿＿＿＿ 容许差距＿＿＿＿ 本次测定:有效□ 无效□

测定人＿＿＿＿ 校核人＿＿＿＿ 测定结束日期＿＿＿＿年＿＿＿月＿＿＿日

C4 生活力测定记录见表C4。

表C4 生活力测定记录表

编号＿＿＿＿

树种＿＿＿＿ 样品号＿＿＿＿ 样品情况＿＿＿＿

染色剂＿＿＿＿ 浓度＿＿＿＿

测试地点＿＿＿＿

环境条件:温度＿＿＿＿℃ 湿度＿＿＿＿%

测试仪器:名称＿＿＿＿ 编号＿＿＿＿

重复	测定种子粒数	种子解剖结果					进行染色粒数	染色结果				平均生活力%	备注
								无生活力		有生活力			
		腐烂粒	涩粒	病虫害粒	空粒			粒数	%	粒数	%		
1													
2													
3													
4													
平均													
测定方法													

实际差距＿＿＿＿ 容许差距＿＿＿＿

本次测定:有效 □ 测定人＿＿＿＿

无效 □ 校核人＿＿＿＿

测定日期＿＿＿年＿＿＿月＿＿＿日

C5 优良度测定记录见表C5。

表C5 优良度测定记录表

编号________

树种____________ 样品号____________ 样品情况____________

测试地点________________________________

环境条件:温度____________℃ 湿度____________%

测试仪器:名称____________ 编号____________

重复	测定种子粒数	观察结果						优良度%	备注
		优良粒	腐烂粒	空粒	涩粒	病虫粒			
1									
2									
3									
4									
平均									
实际差距			容许差距						
测定方法									

本次测定:有效 □

无效 □

测定人________

校核人________

测定日期____年____月____日

C6 种子健康状况测定记录见表C6。

表C6 种子健康状况测定记录表

编号________

树种____________ 样品号____________ 样品情况____________

测试地点________________________________

环境条件:温度____________℃ 湿度____________%

测试仪器:名称____________ 编号____________

测定种子粒数	观察结果				病害感染度%	虫害感染度%	病虫害感染度%	备注
	健康粒	虫粒	病粒					
测定方法								

测定人________

校核人________

测定日期____年____月____日

C7 含水量测定记录见表C7。

表C7 含水量测定记录表

编号＿＿＿＿

树种＿＿＿＿ 样品号＿＿＿＿ 样品情况＿＿＿＿

测试地点＿＿＿＿

环境条件:温度＿＿＿＿℃ 湿度＿＿＿＿%

测试仪器:名称＿＿＿＿编号＿＿＿＿

测定方法＿＿＿＿

容器号			
容器重,g			
容器及测定样品原重,g			
烘至恒重,g			
测定样品原重,g			
水分重,g			
含水量,%			
平均	%		
实际差距	%	容许差距	%

本次测定:有效 □ 测定人＿＿＿＿

无效 □ 校核人＿＿＿＿

测定日期＿＿年＿＿月＿＿日

C8 重量测定记录见表C8。

表C8 重量测定记录表

编号＿＿＿＿

树种＿＿＿＿ 样品号＿＿＿＿ 样品情况＿＿＿＿ 测试地点＿＿＿＿

环境条件:室内温度＿＿＿＿C 湿度＿＿＿＿% 测试仪器:名称＿＿＿＿ 编号＿＿＿＿

测定方法＿＿＿＿

重复号	1	2	3	4	5	6	7	8	9	10	11	12	13	14	15	16
x,g																
标准差(S)																
$\bar{x}$																
变异系数																
千粒重,g $10\times\bar{x}$																

第 组数据超过了容许误差,本次测定根据第 组计算。

本次测定:有效□ 测定人＿＿＿＿ 校核人＿＿＿＿ 测定日期＿＿年＿＿月＿＿日

无效□

附 录 D
（标准的附录）
质量检验证书

表 D1 种子样品质量检验证书

编号__________

据送检人陈述

树种中名__________树种学名__________产地__________

种批编号	种批重,kg	容器件数	抽样日期	送检样品号

送检人__________单位地址__________邮政编码__________

正式报告

样品编号	样品封缄	样品重,g	样品收到日期	检验结束日期

检验结果

被检树种中名__________树种学名__________

净度测定			发芽测定						千粒重 %	含水量 %	生活力 %	优良度 %	病虫害感染度 %
%			天数	%									
纯净种子（即净度）	夹杂物	其他种子		正常幼苗（即发芽率）	不正常幼苗	新鲜粒	硬粒	死亡粒					
1	2	3	4	5	6	7	8	9	10	11	12	13	14
分级依据								质量等级					
备注													

检验机构全称__________ 主检人__________

地址__________ 校核人__________

邮编__________ 技术负责人__________

电话__________ 签发日期______年____月____日

检验机构（章）

（背面）

签发机构声明

1. 检验的程序和方法符合国家标准 GB 2772—1999《林木种子检验规程》，所有检验均在本机构进行，（省级以上行政机构）已授权本机构签发种子样品质量检验证书。
2. 证书无检验单位盖章及技术负责人签字无效，涂改无效。
3. 本证书检验结果只对送检样品负责。

表 D2　种批质量检验证书

编号＿＿＿＿＿＿＿＿

据送检人陈述

树种中名＿＿＿＿＿＿树种学名＿＿＿＿＿＿产地＿＿＿＿＿＿
送检人＿＿＿＿＿＿地址＿＿＿＿＿＿邮政编码＿＿＿＿＿＿

正式报告

抽样、封缄单位和人员＿＿＿＿＿＿
种批标记＿＿＿＿＿＿
种批封缄＿＿＿＿＿＿

种批重 kg	容器名称	容器件数	抽样日期	样品重 g	样品编号	样品收到日期	检验结束日期

检验结果

被检树种中名＿＿＿＿＿＿树种学名＿＿＿＿＿＿

净度测定			发芽测定						千粒重 %	含水量 %	生活力 %	优良度 %	病虫害感染度 %
%			天数	%									
纯净种子（即净度）	夹杂物	其他种子		正常幼苗（即发芽率）	不正常幼苗	新鲜粒	硬粒	死亡粒					
1	2	3	4	5	6	7	8	9	10	11	12	13	14
分级依据								质量等级					
备注													

检验机构全称＿＿＿＿＿＿　　主检人＿＿＿＿＿＿
地址＿＿＿＿＿＿　　校核人＿＿＿＿＿＿
邮编＿＿＿＿＿＿　　技术负责人＿＿＿＿＿＿
电话＿＿＿＿＿＿　　签发日期＿＿＿＿年＿＿月＿＿日

检验机构（章）

（背面）

签发机构声明

1. 本种批的抽样、封缄和检验均符合国家标准 GB 2772—1999《林木种子检验规程》，所有检验均在本机构进行，　　　（省级以上行政机构）已授权本机构签发种批质量检验证书。

2. 证书无检验单位盖章及技术负责人签字无效，涂改无效。

3. 本批种子分别调往不同用种单位时，本证书检验结果无效。

附　录　E
（提示的附录）
检验情况综合表

表 E1　　　　编号

卷　宗　号					
样品登记号					
样品收到日期					
据送检人陈述	送检单位				
	检验申请表编号				
	种批号				
	树　　种				
	产　　地				
	送检样品重<br>g				
	本批种子重<br>kg				
检验结果	检验结束日期				
	净　度，%				
	千粒重，g				
	发芽率，%				
	优良度，%				
	生活力，%				
	含水量，%				
	病虫害感染度，%				
	本批种子等级意见				
	质量检验证书名称				
	质量检验证书号				
	发证日期				
	备　　注				

注：本表用于检验机构存档

登记人__________　　日期______年______月______日

附 录 F

（提示的附录）

乔灌木种子示意图

表 F1

1 冷杉属 *Abies*

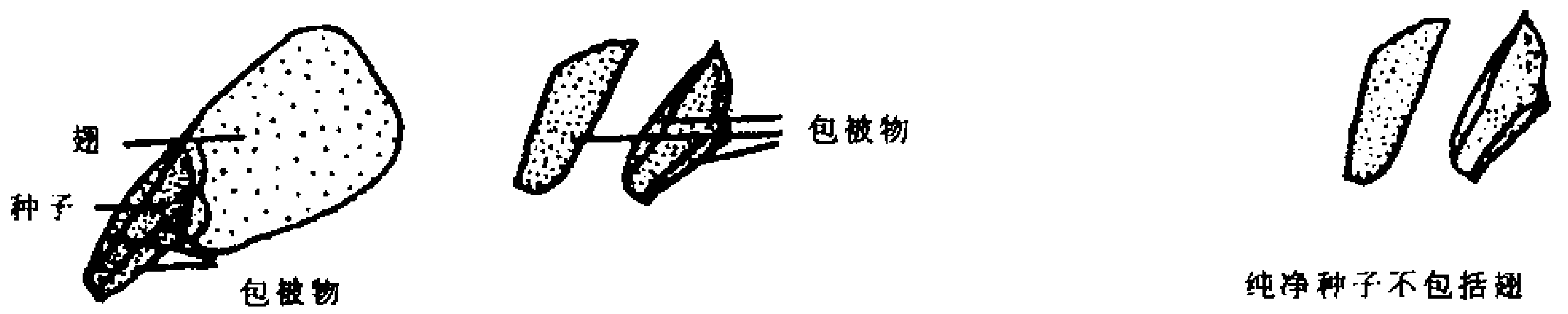

2 雪松属 *Cedrus*

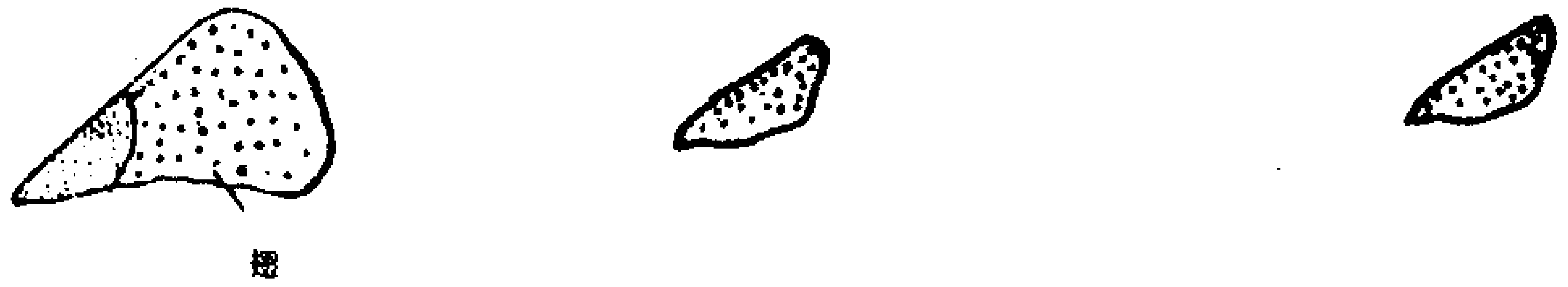

3 扁柏属 *Chamaecyparis*

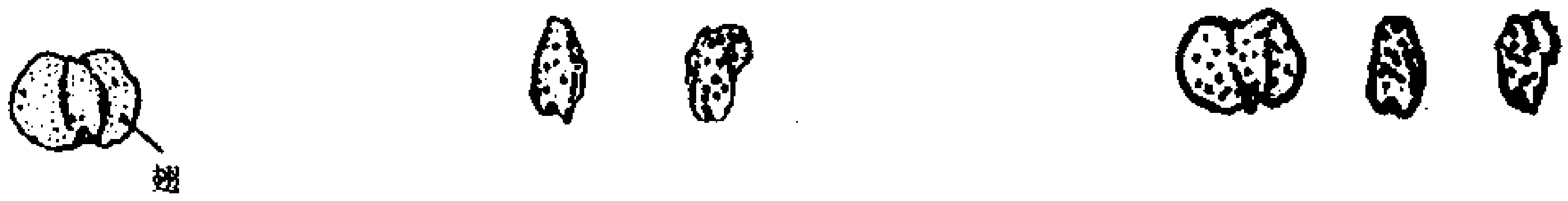

4 柳杉属 *Cryptomeria*

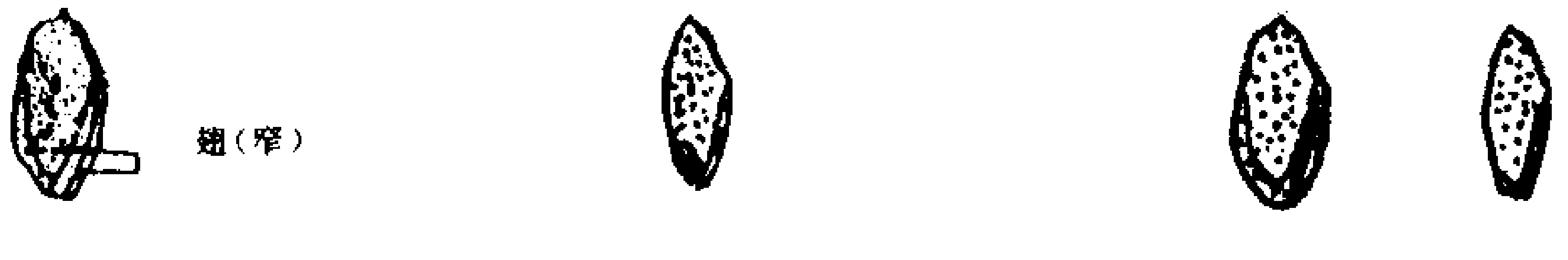

表 F1（续）

5 柏木属 *Cupressus*

带翅种子

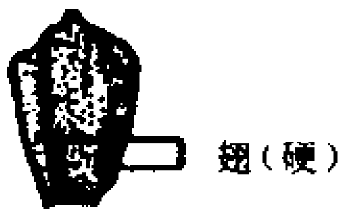

纯净种子

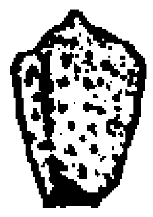

6 刺柏属 *Juniperus*

种子

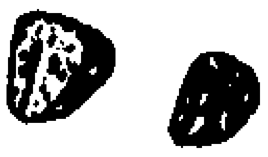

纯净种子

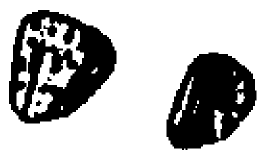

7 落叶松属 *Larix*

带翅种子

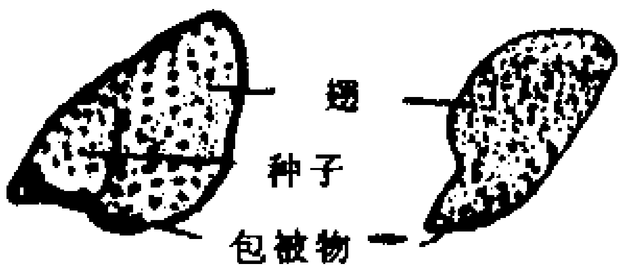

正面 背面

去翅种子
但仍具包被物

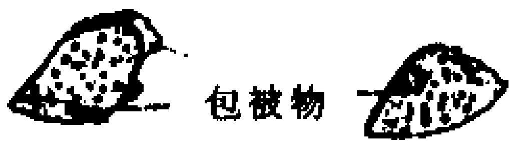

正面 背面

纯净种子

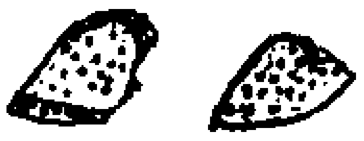

纯净种子不包括翅

8 云杉属 *Picea*

带翅种子

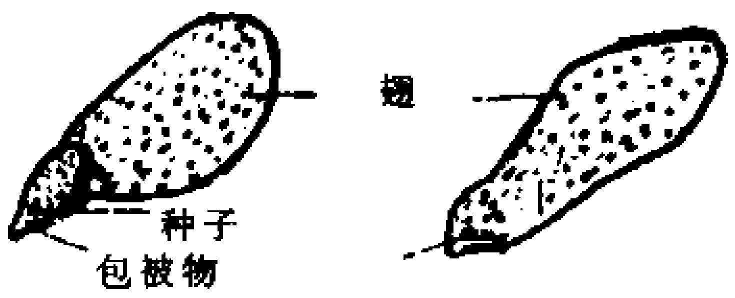

正面 背面

去翅种子
但仍具包被物

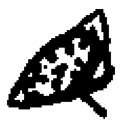

种子

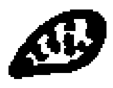

包被物

背面

纯净种子

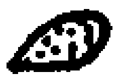

纯净种子不包括翅和包被物

表 F1（续）

9　松属　*Pinus*

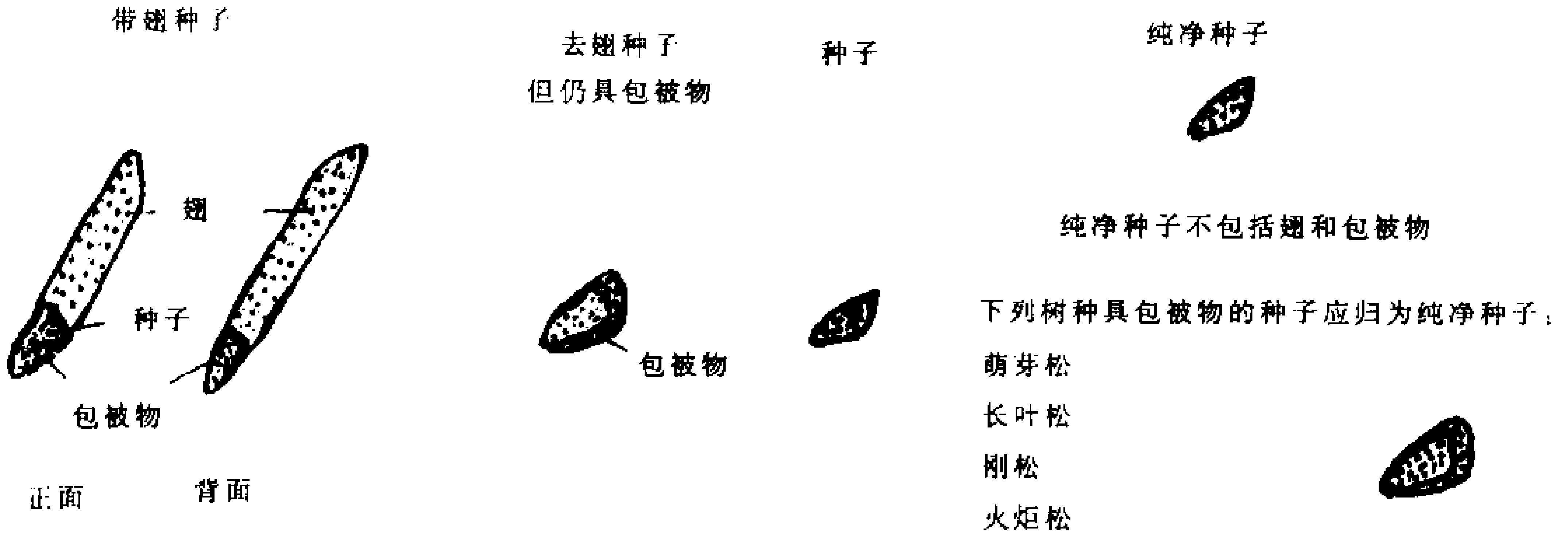

纯净种子不包括翅和包被物

下列树种具包被物的种子应归为纯净种子：

萌芽松

长叶松

刚松

火炬松

10　落羽杉属　*Taxodium*

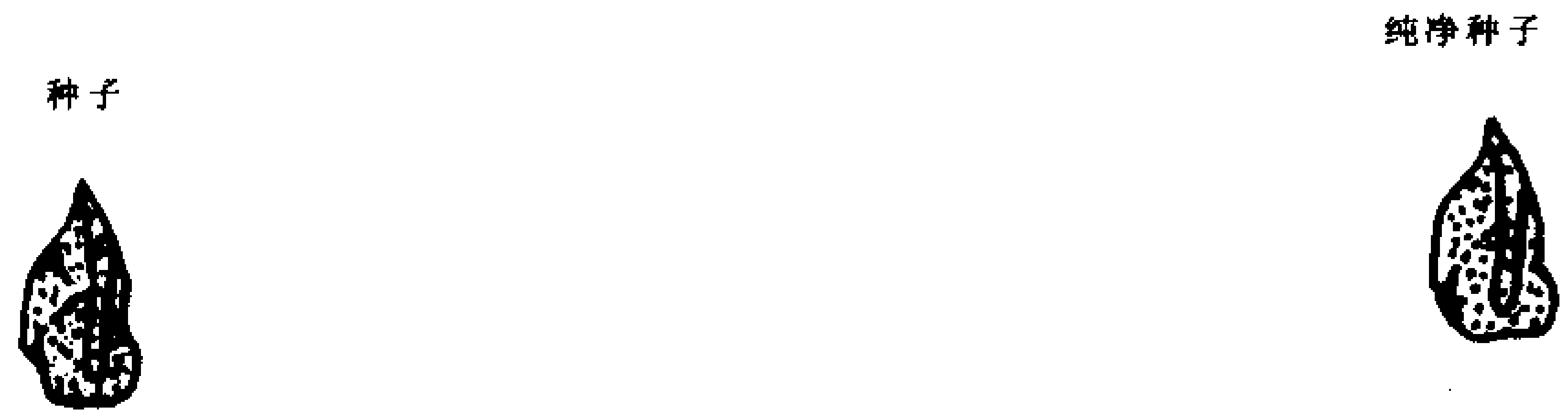

11　紫杉属（红豆杉属）　*Taxus*

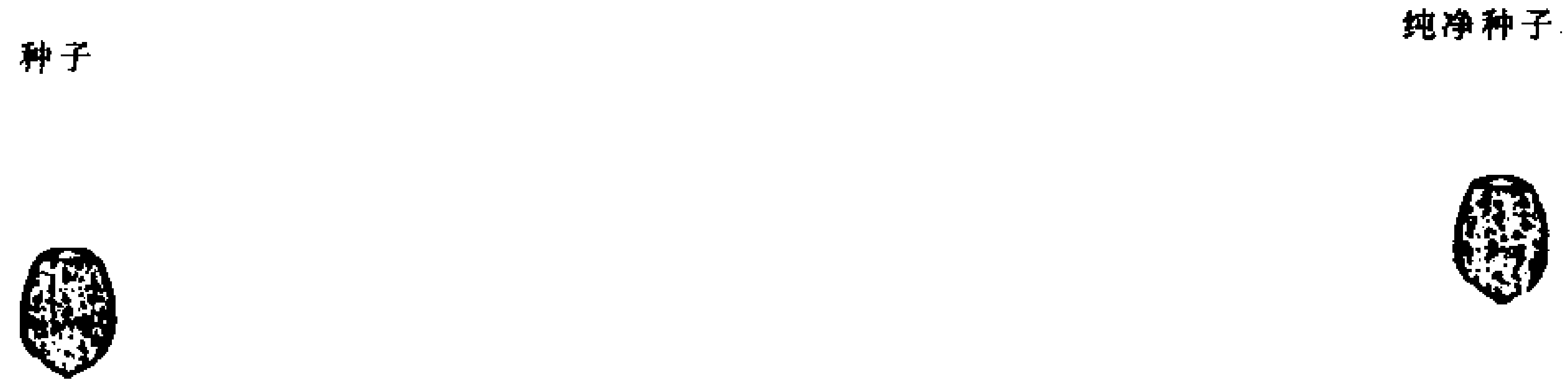

12　金合欢属　*Acacia*

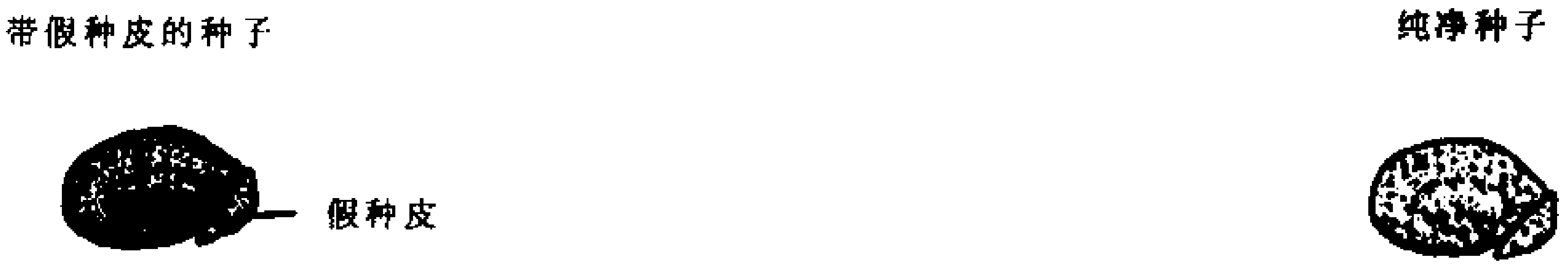

13　槭属　*Acer*

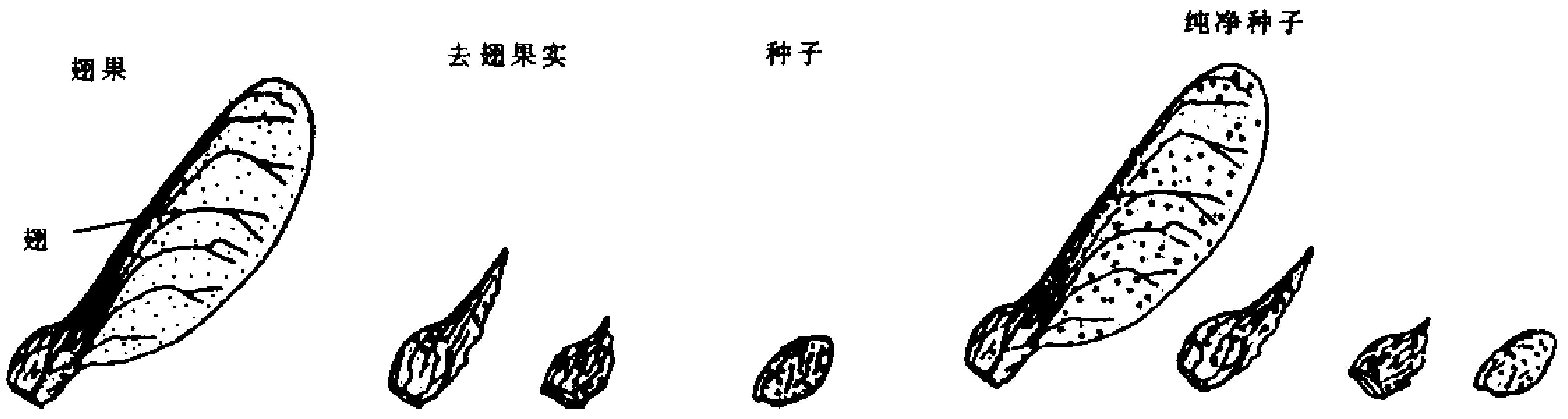

表 F1（续）

14 臭椿属 *Ailanthus*

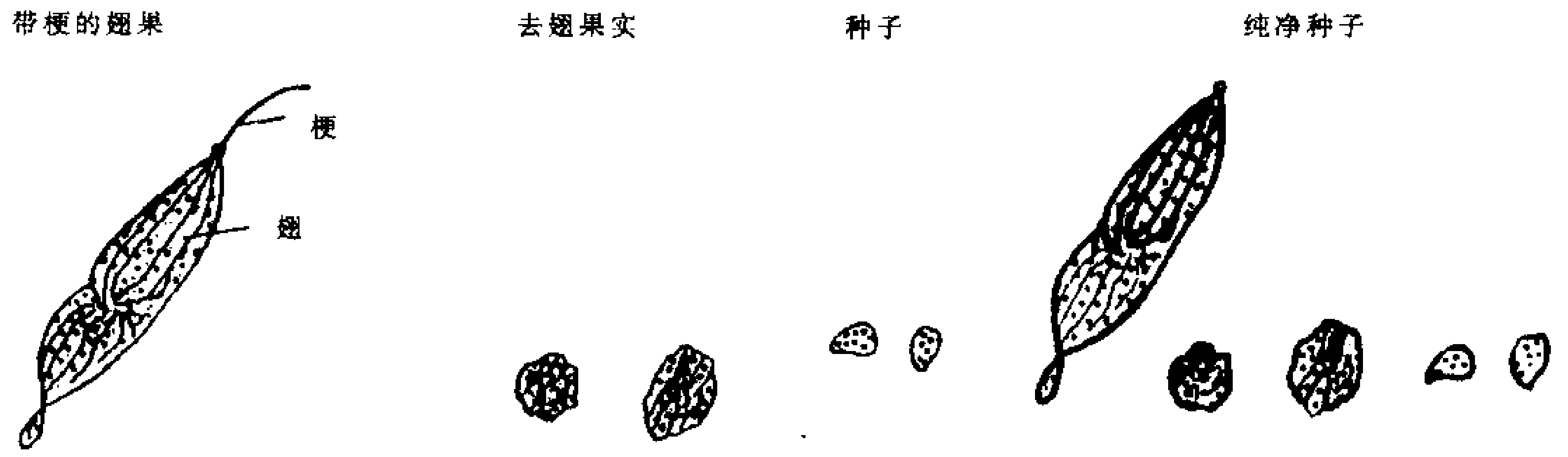

纯净种子不包括梗

15 桤木属 *Alnus*

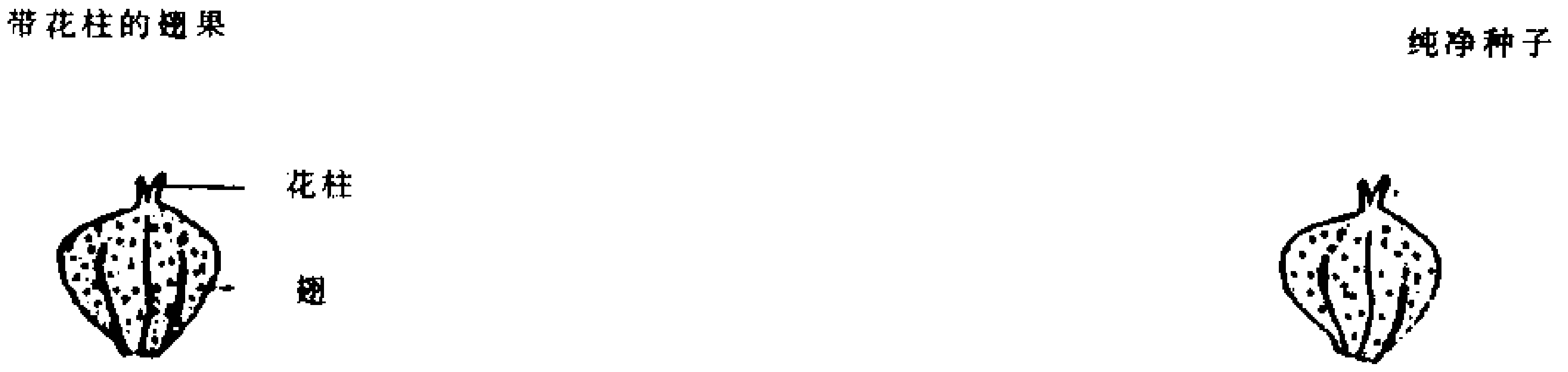

16 小檗属 *Berberis*

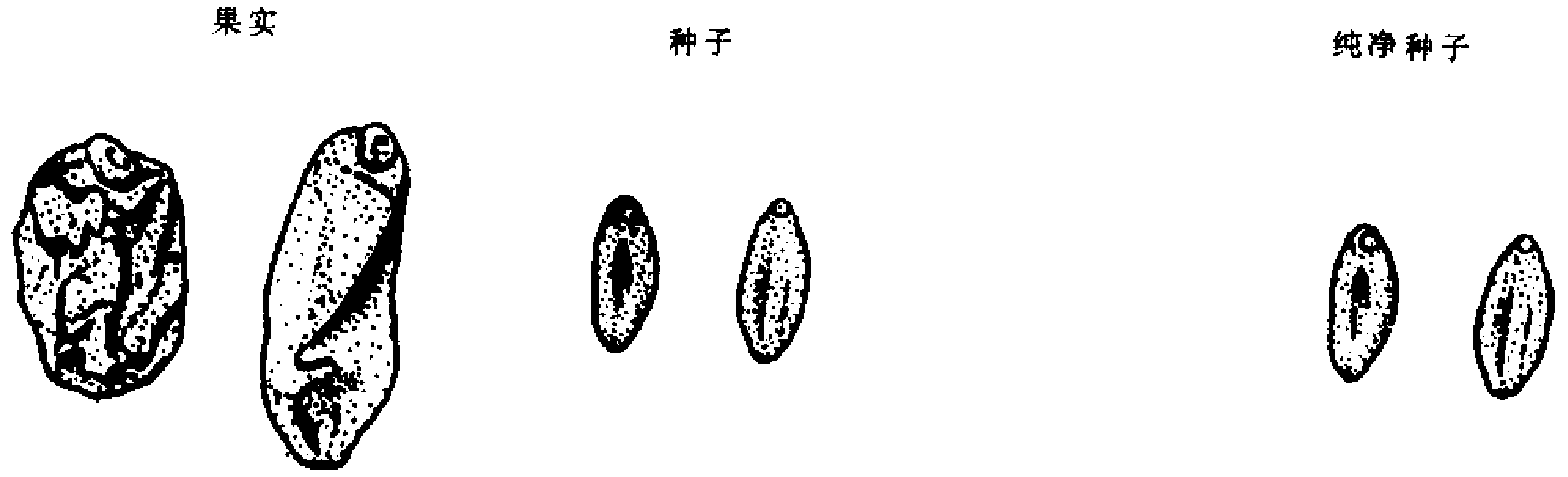

17 桦木属 *Betula*

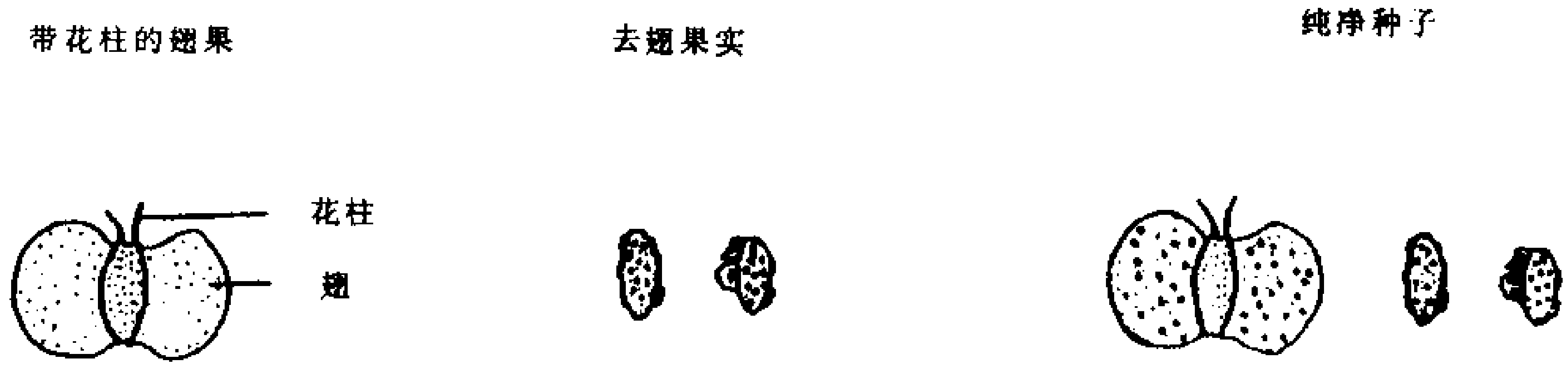

表 F1（续）

18 鹅耳枥属 *Carpinus*

带柄及总苞的坚果 | 坚果 | 纯净种子

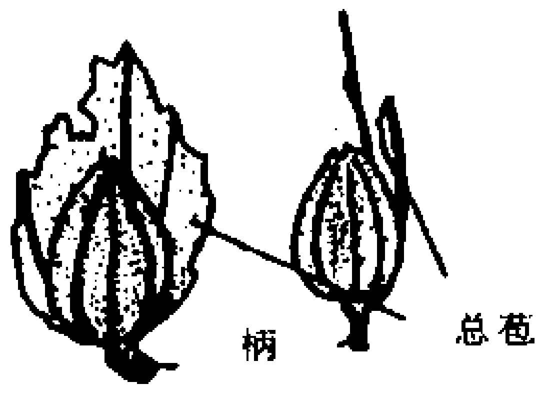

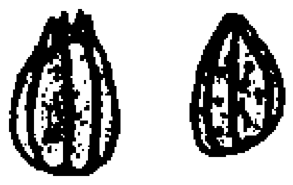

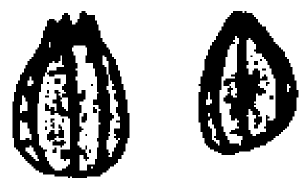

纯净种子不包括总苞和柄

19 山核桃属 *Carya*

果实 | 小坚果 | 纯净种子

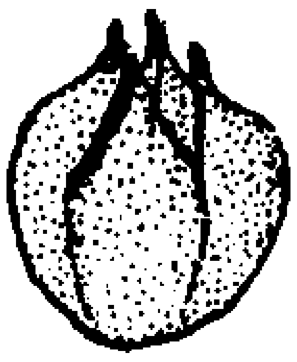

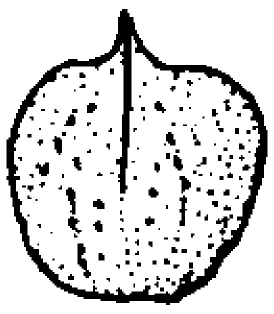

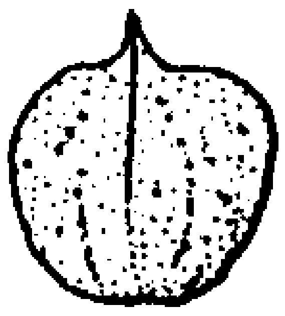

20 梓属 *Catalpa*

带翅的种子 | 纯净种子

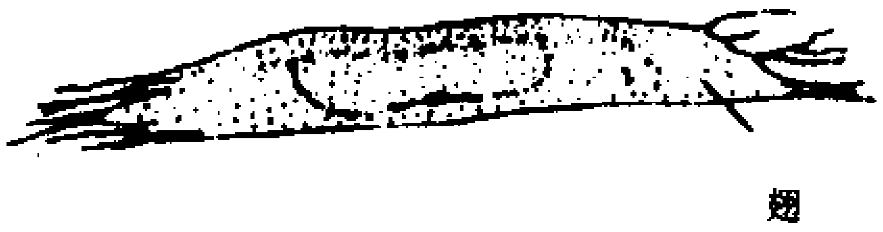

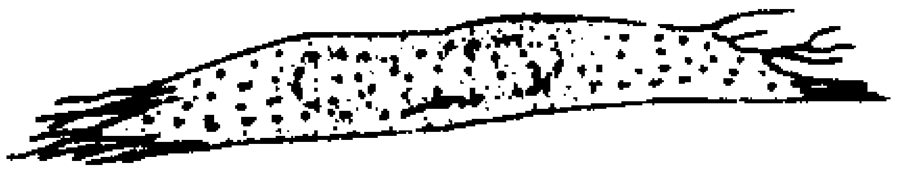

21 梾木属 *Cornus*

果实 | 小坚果 | 种子 | 纯净种子

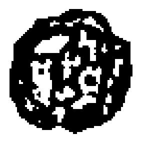

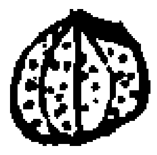

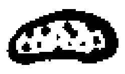

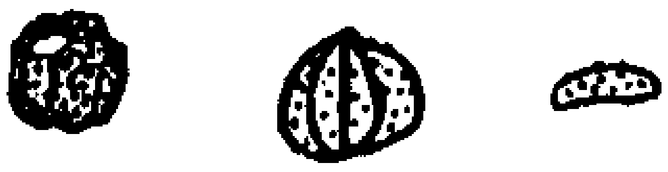

22 榛属 *Corylus*

坚果 | 种子 | 纯净种子

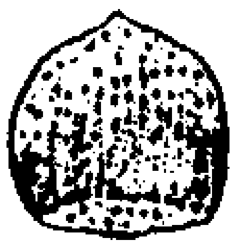

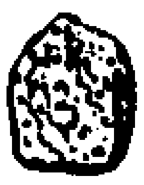

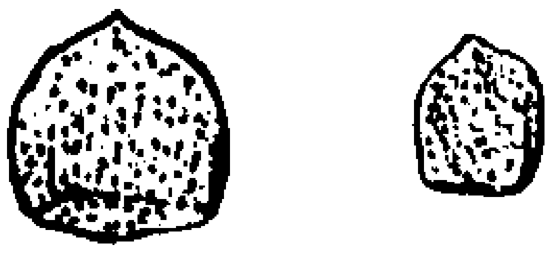

表 F1（续）

23　黄栌属　*Cotinus*

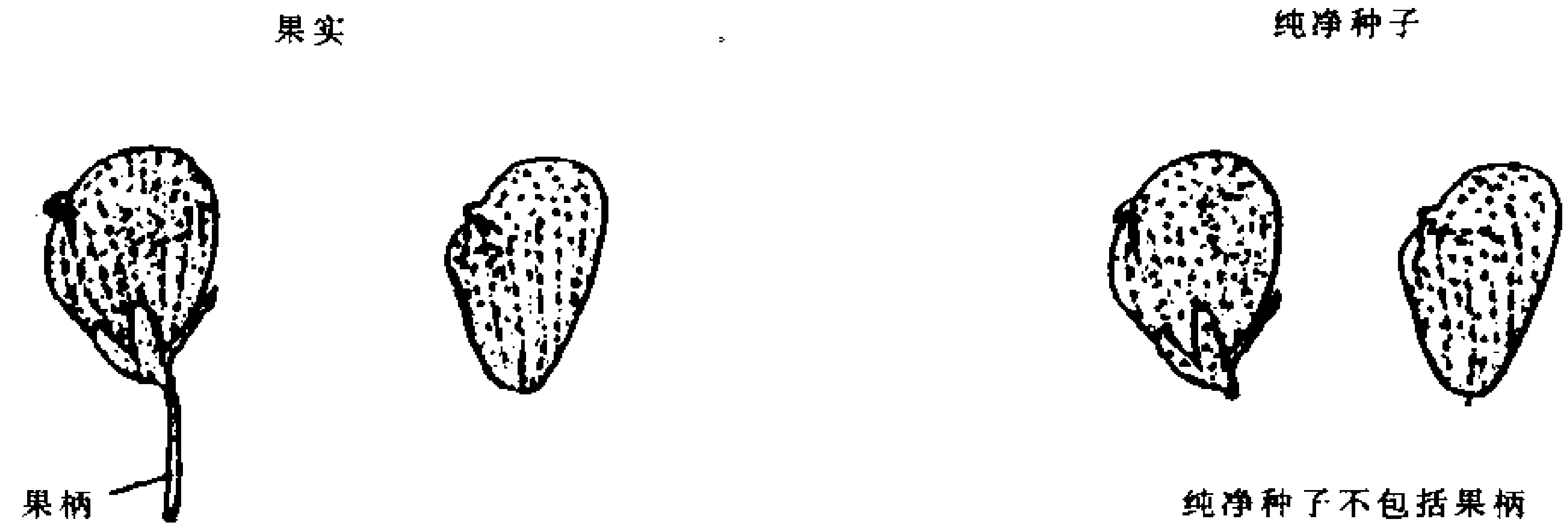

24　山楂属　*Crataegus*

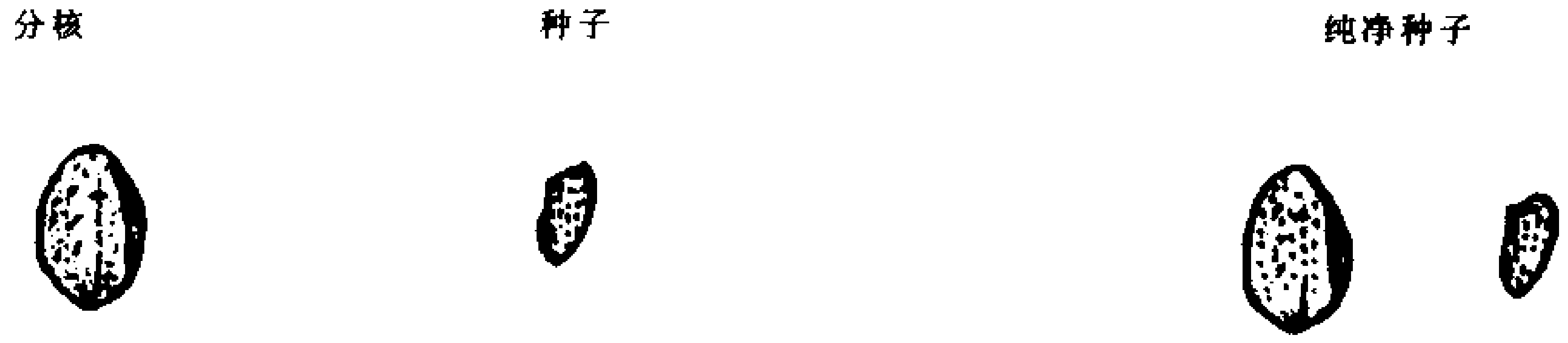

25　沙枣属(胡颓子属)　*Elaeagnus*

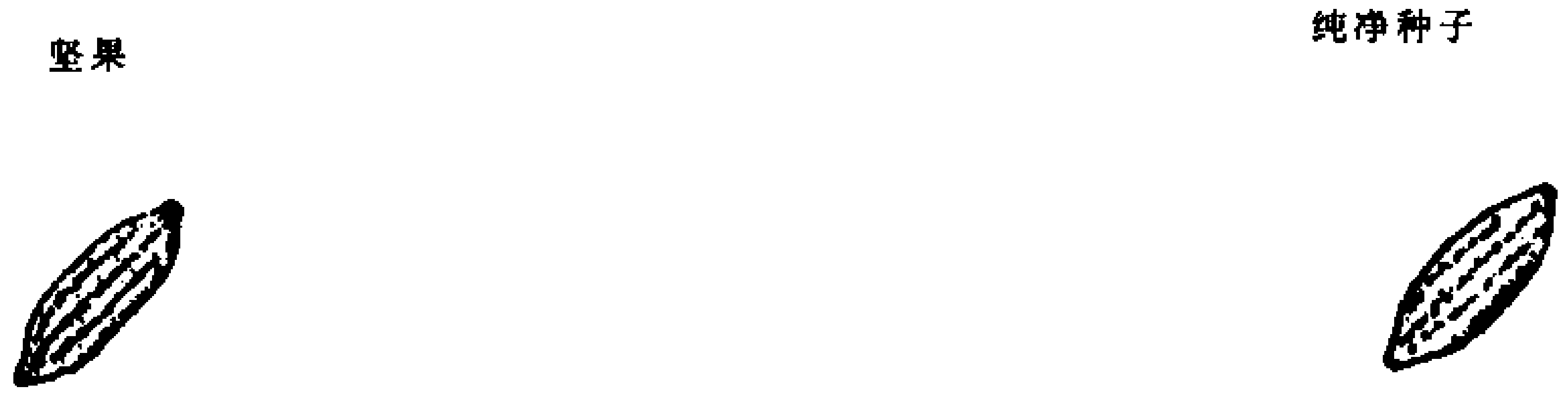

26　桉属　*Eucalyptus*

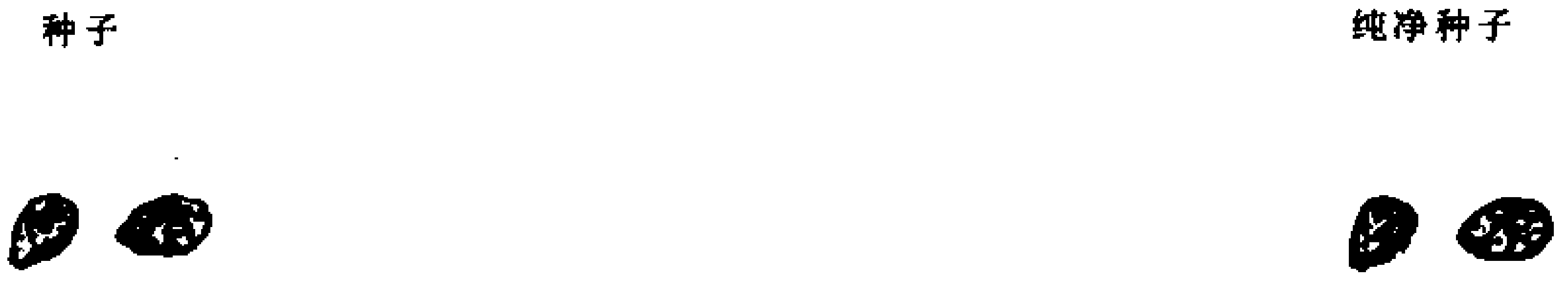

27　卫矛属　*Euonymus*

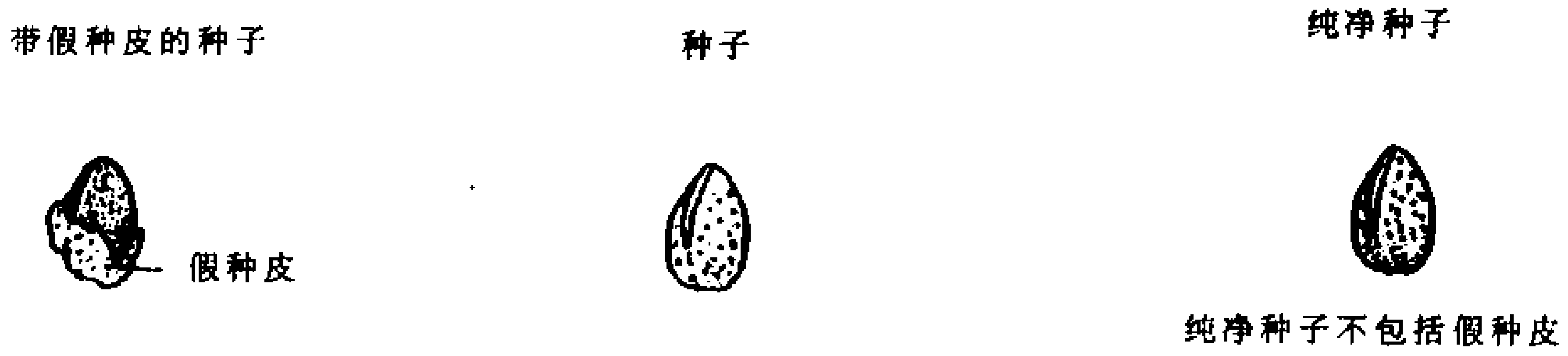

表 F1（续）

28 水青冈属 *Fagus*

坚果　　种子　　纯净种子

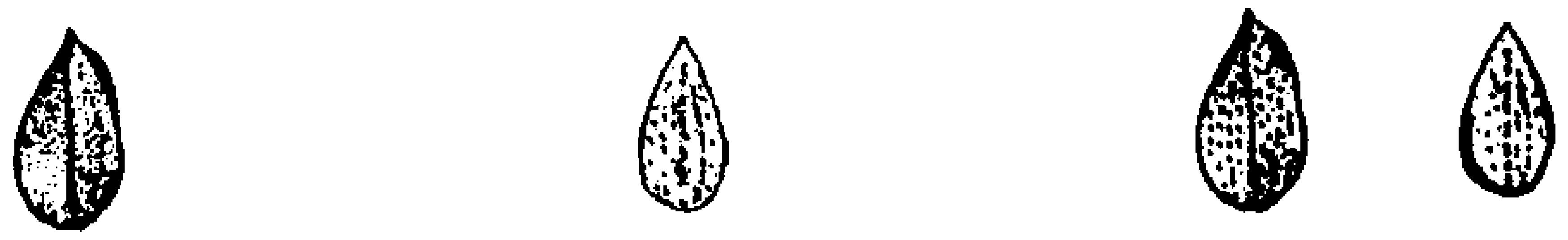

29 白蜡树属 *Fraxinus*

带果柄的翅果　　去翅果实　　种子　　纯净种子

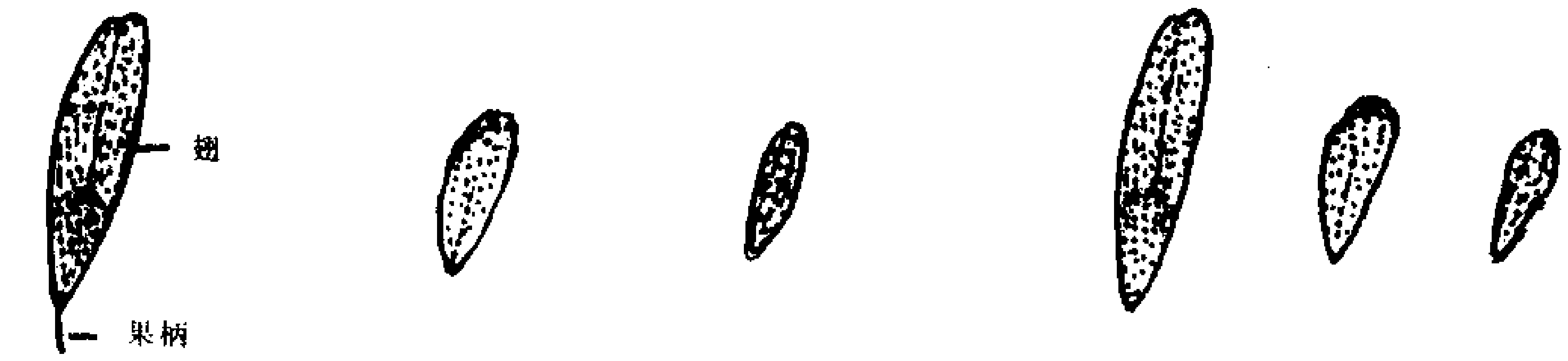

纯净种子不包括果柄

30 沙棘属 *Hippophae*

分核　　纯净种子

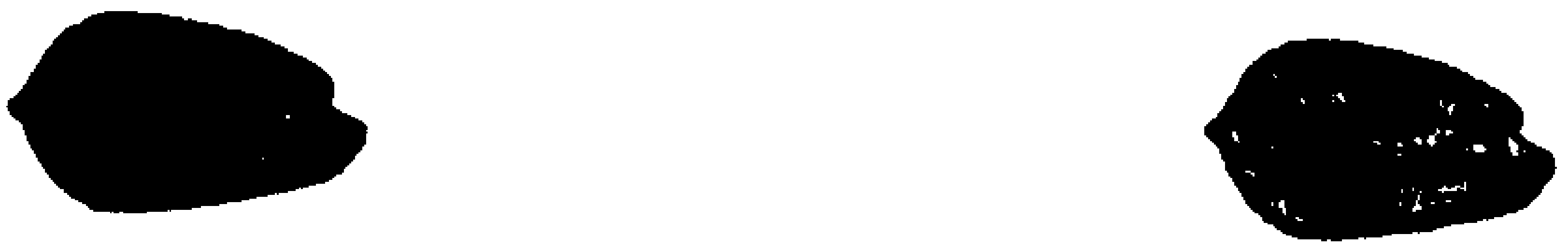

31 冬青属 *Ilex*

果实　　分核　　纯净种子

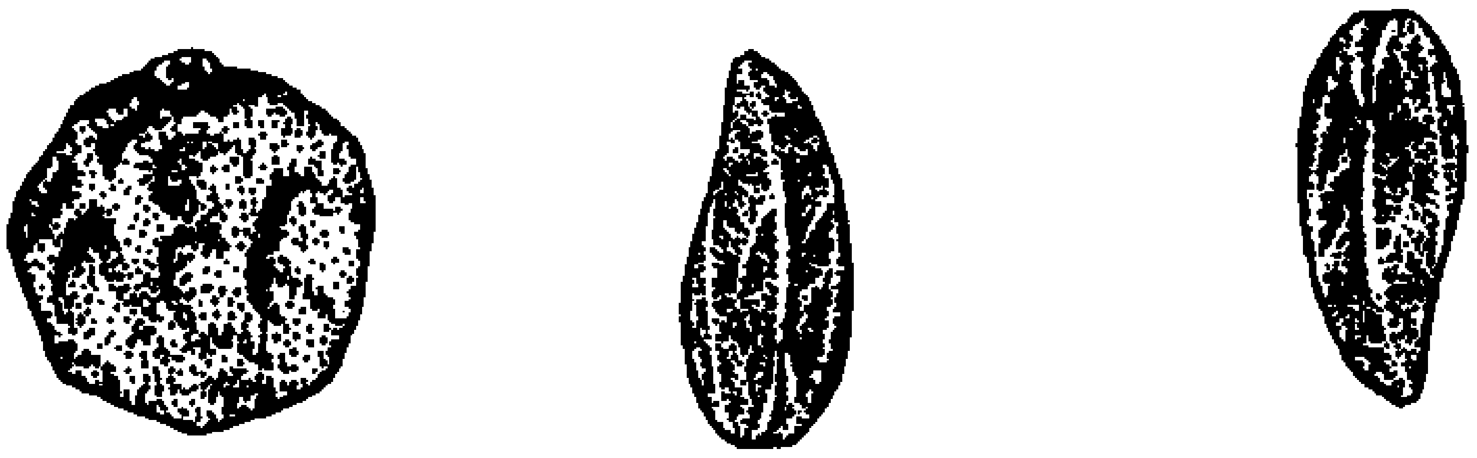

32 胡桃属 *Juglans*

分核　　种子　　纯净种子

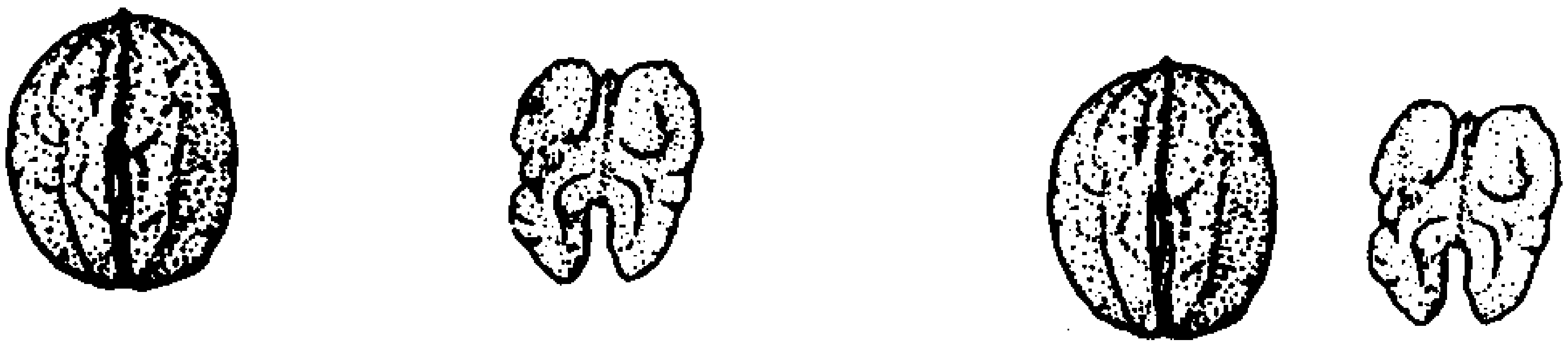

表 F1（续）

33 栾树属 *Koelreuteria*

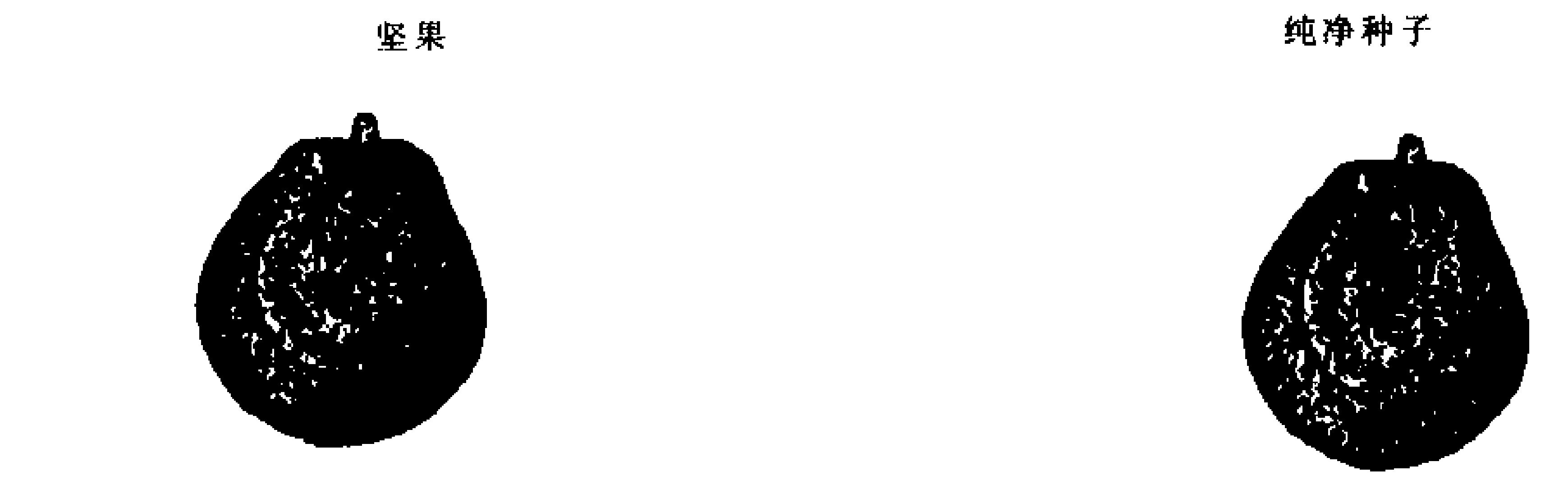

34 枫香属 *Liquidambar*

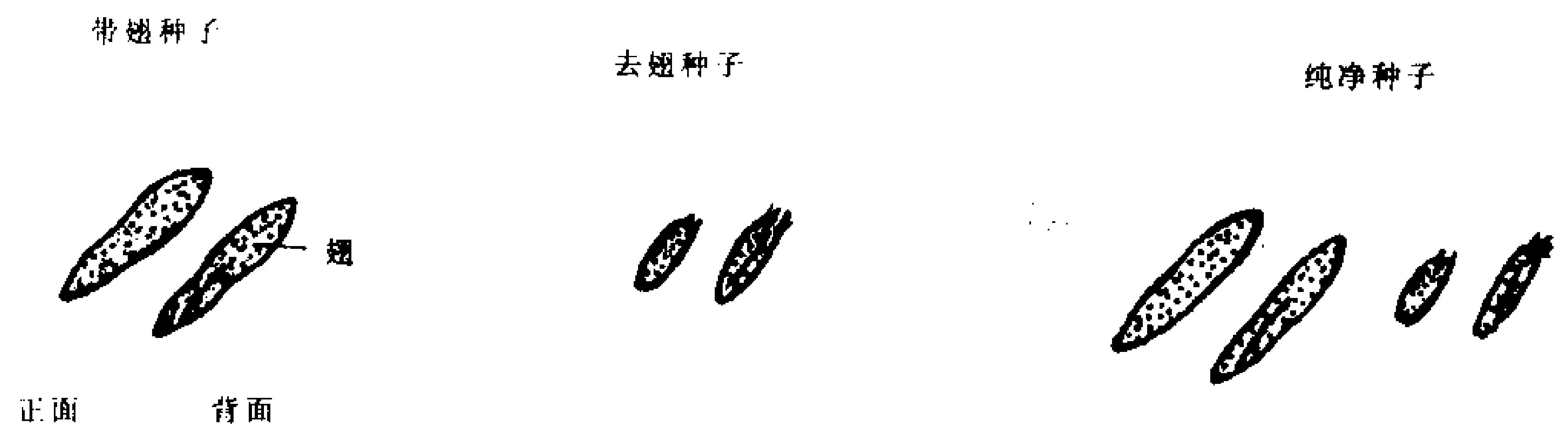

35 鹅掌楸属 *Liriodendron*

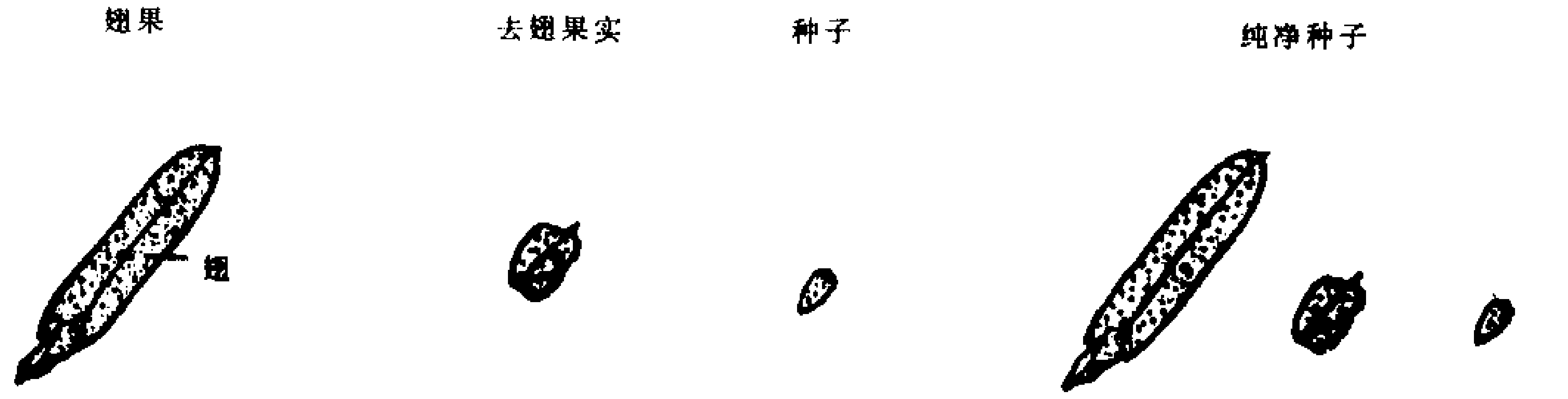

36 忍冬属 *Lonicera*

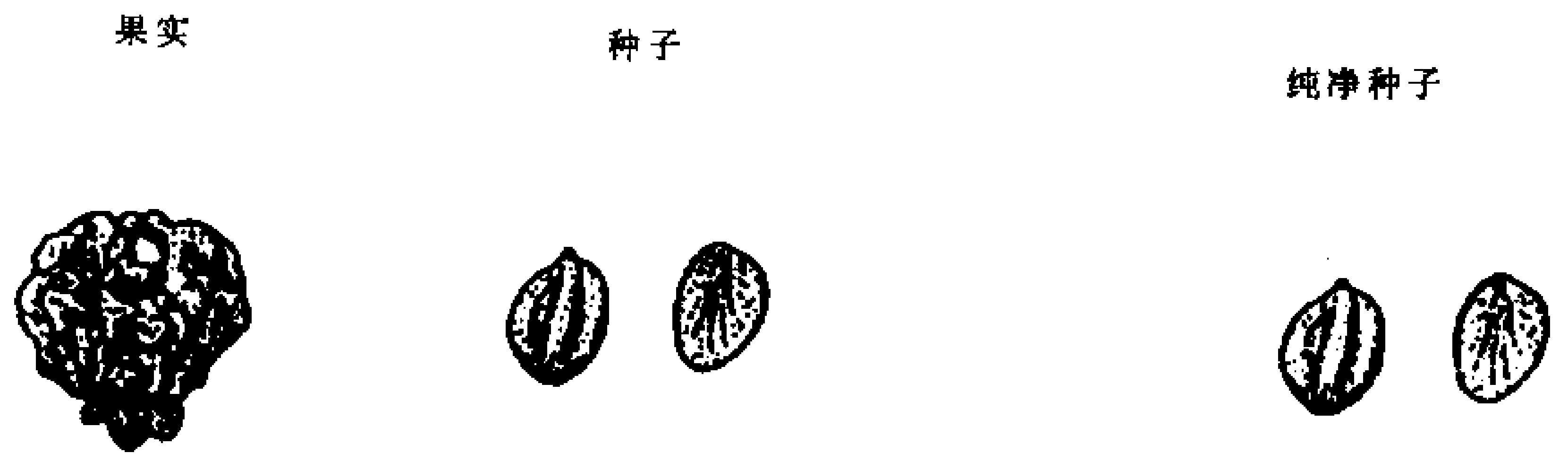

37 木兰属 *Magnolia*

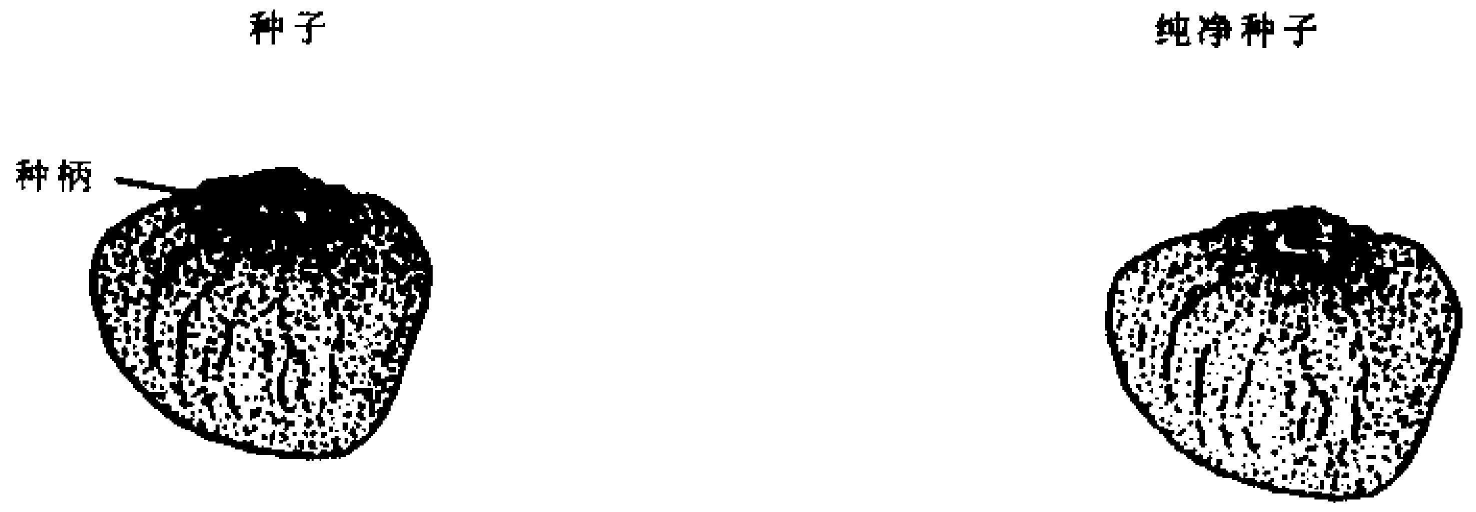

表 F1（续）

38　桑属　*Morus*

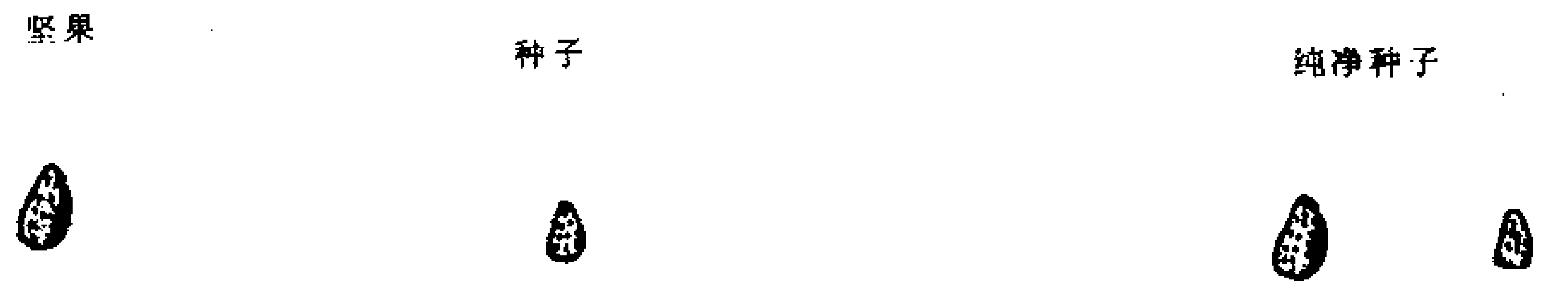

39　黄柏属　*Phellodendron*

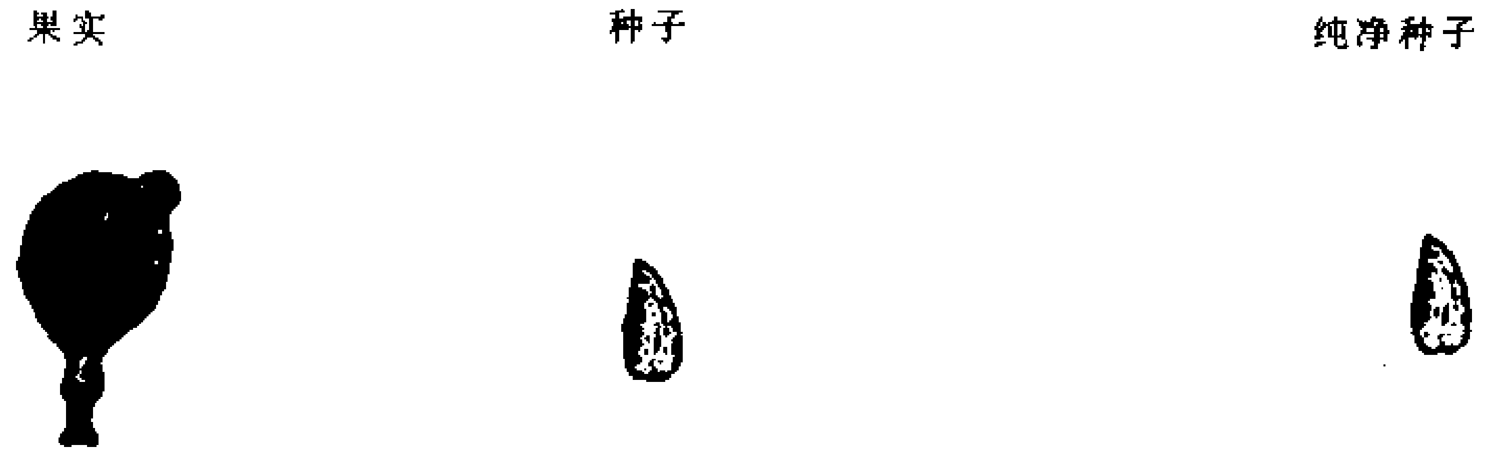

40　悬铃木属　*Platanus*

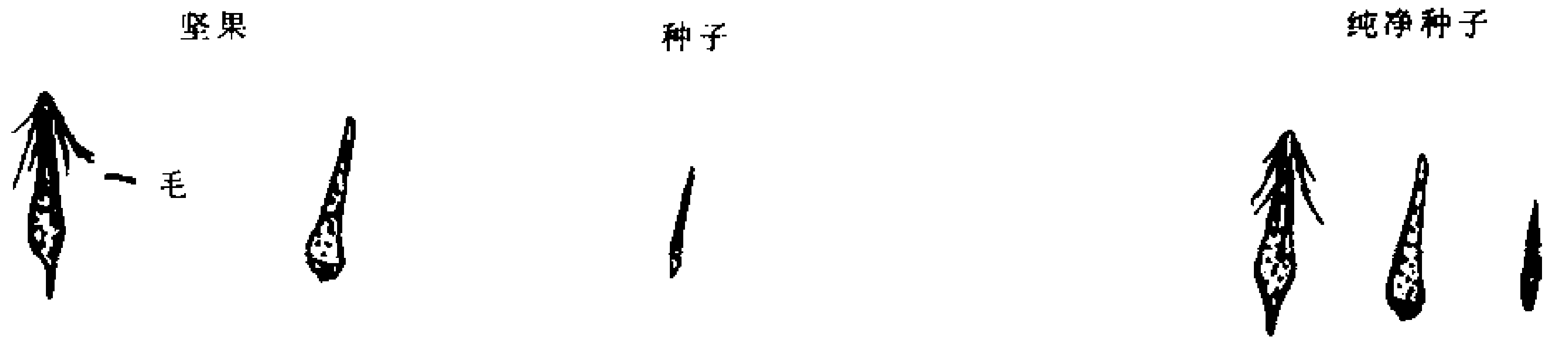

41　杨属　*Populus*

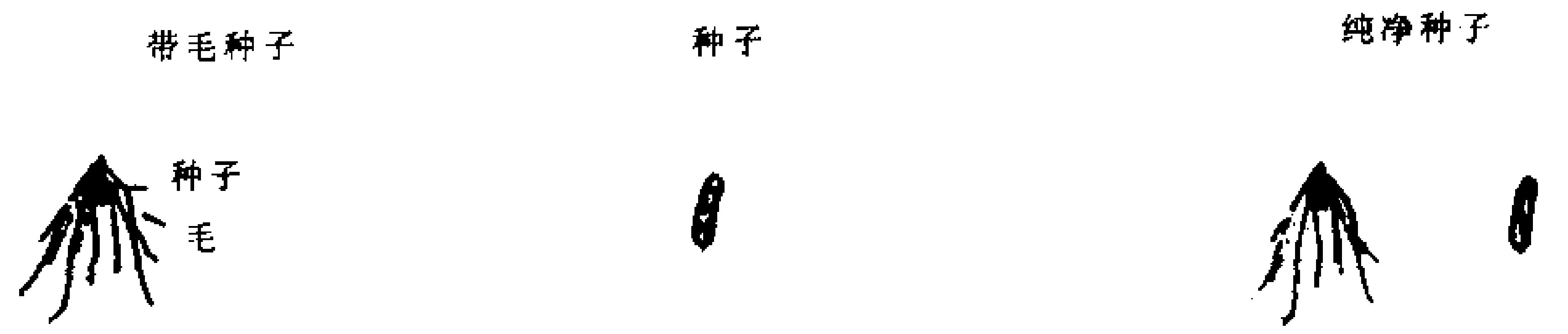

42　李属　*Prunus*

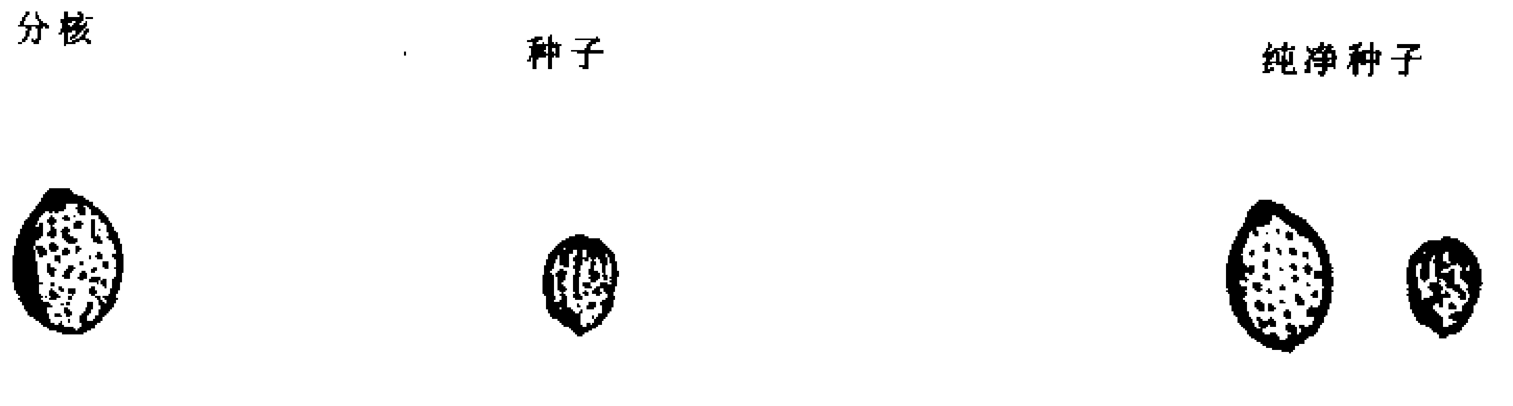

43　栎属　*Quercus*

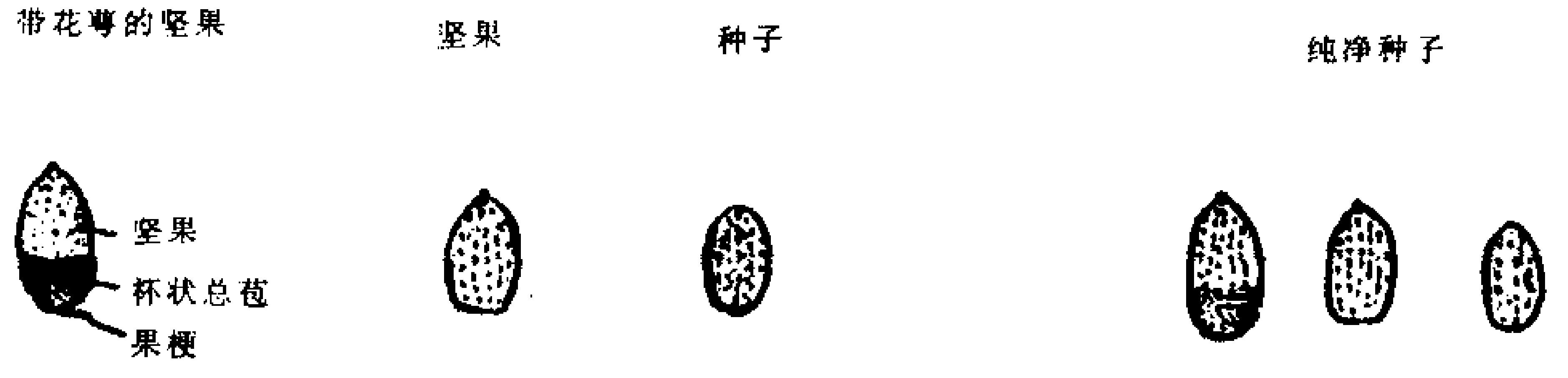

纯净种子不包括果梗

表 F1（续）

44 盐肤木属 *Rhus*

果实　　种子　　纯净种子

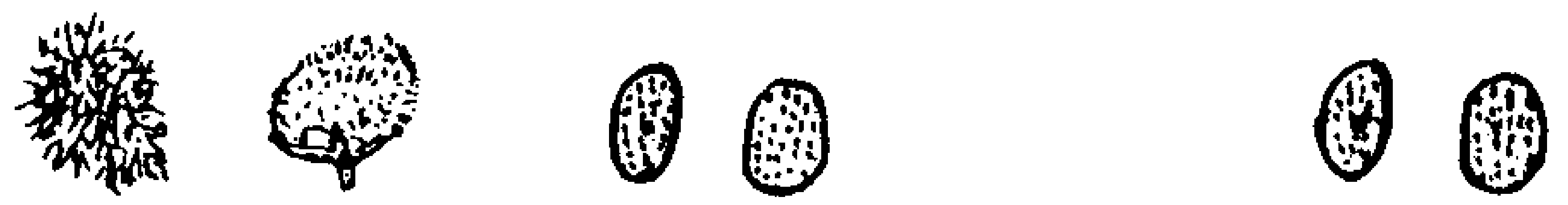

45 刺槐属 *Robinia*

带柄的种子　　种子　　纯净种子

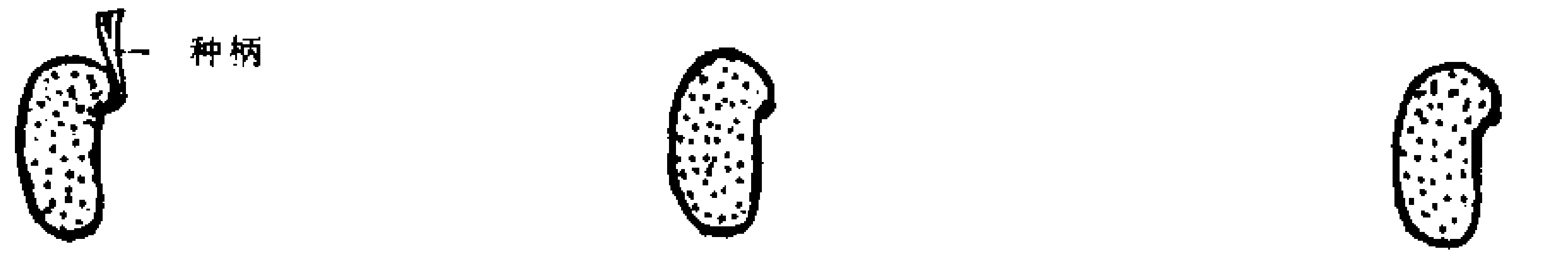

纯净种子不包括种柄

46 柳属 *Salix*

带毛的种子　　种子　　纯净种子

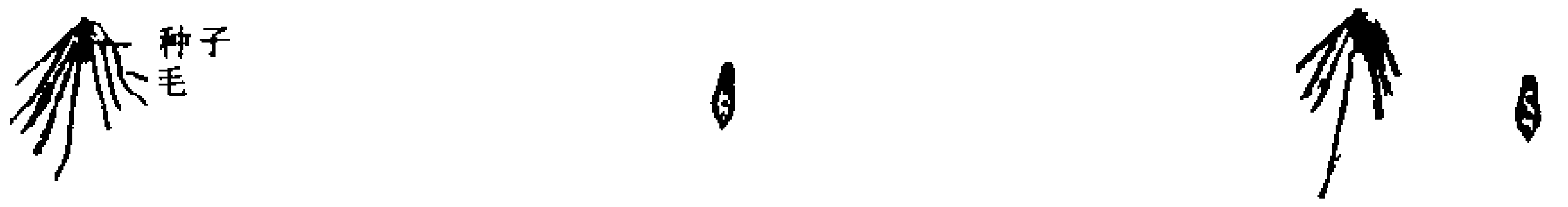

47 丁香属 *Syringa*

带翅种子　　去翅种子　　纯净种子

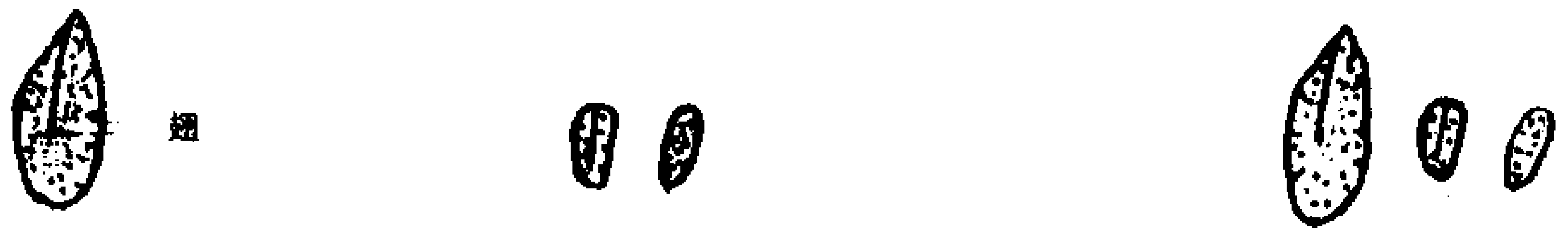

48 柚木属 *Tectona*

带萼的果实（萼已部分脱去）　　果实　　种子　　纯净种子

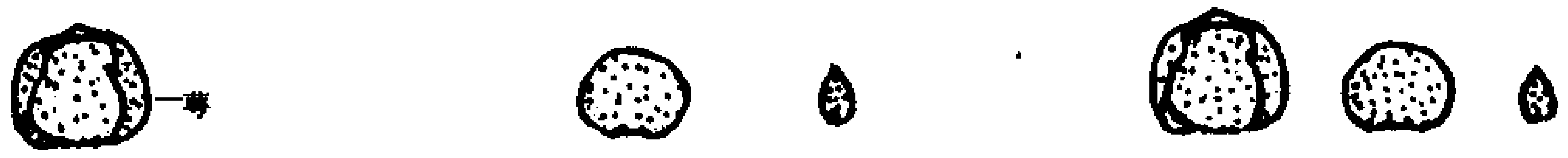

表 F1（完）

49 椴属 *Tilia*

坚果 种子 纯净种子

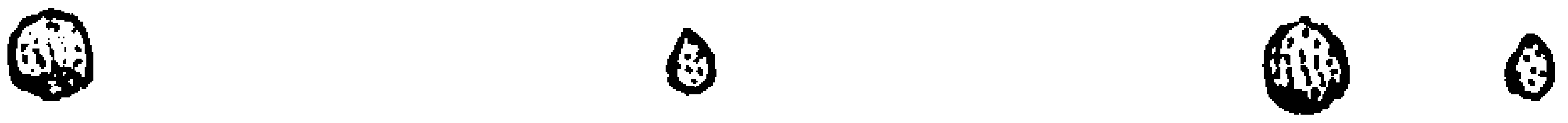

50 榆属 *Ulmus*

翅果 种子 纯净种子

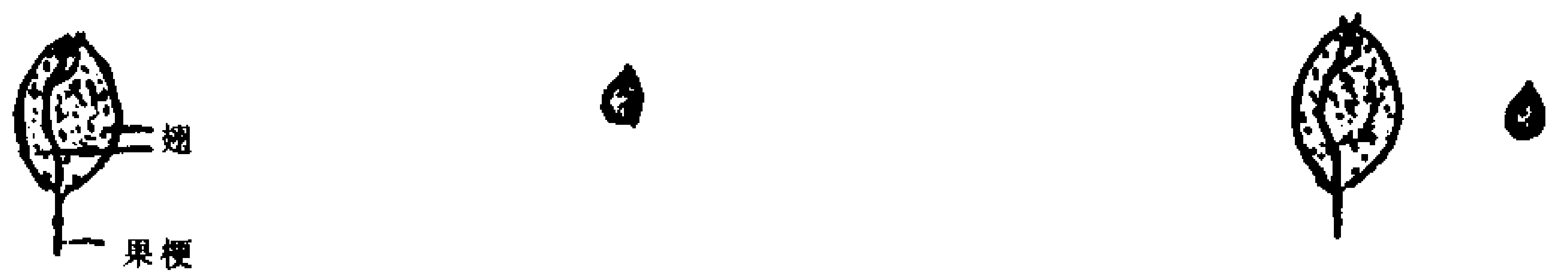

51 荚蒾属 *Viburnum*

果实 分核 纯净种子

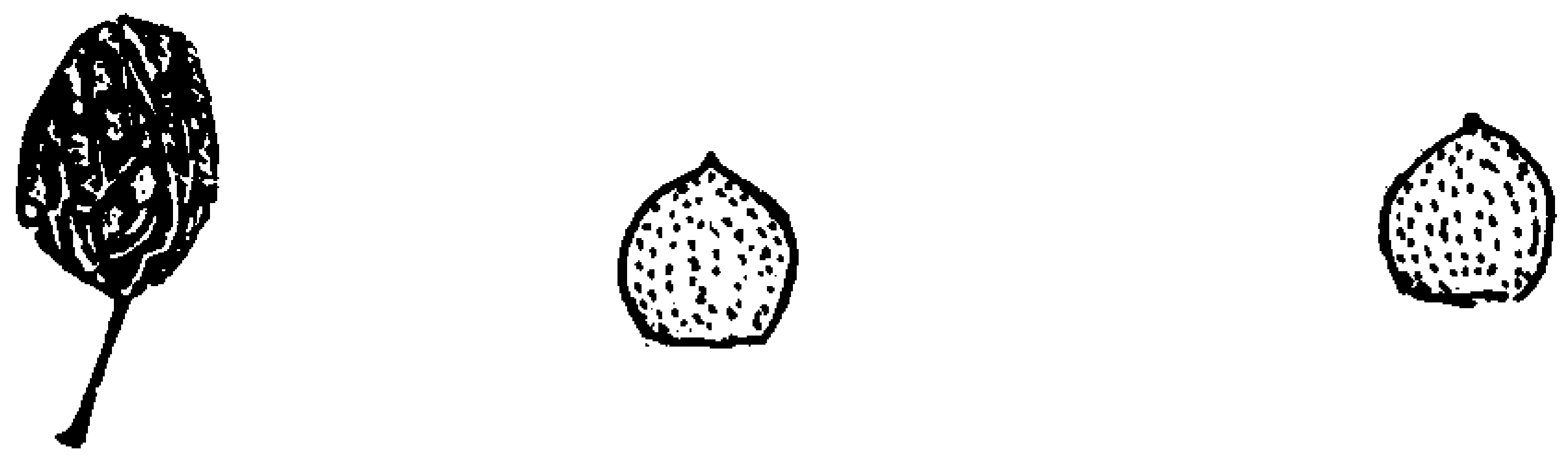

52 榉属 *Zelkova*

坚果 纯净种子

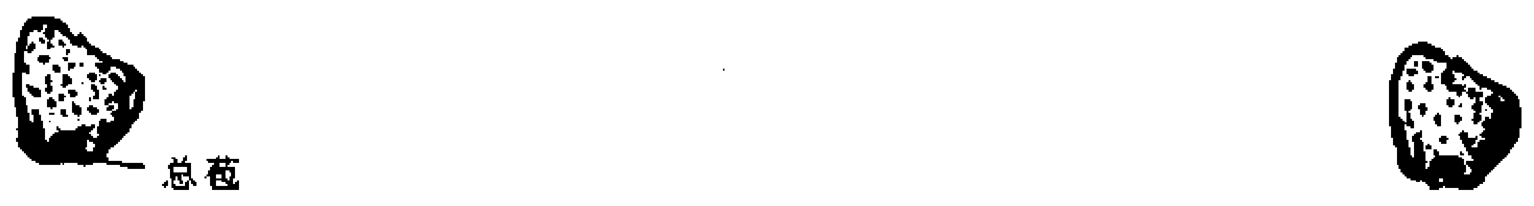

从坚果中脱出的种子也为纯净种子(未绘出)

注：本图引自高捍东等译，国际种子检验协会《乔灌木种子手册》，东南大学出版社，1994
