# Supplementary material for: A Strategy Based on GC-MS/MS, UPLC-MS/MS and Virtual Molecular Docking for Analysis and Prediction of Bioactive Compounds in Eucalyptus Globulus Leaves
Source: Int J Mol Sci. 2019 Aug 8;20(16):3875. doi: 10.3390/ijms20163875 (PMC6721025; doi:10.3390/ijms20163875)

Supplementary Materials

Structure of compounds in aqueous extracts from the *E. globulus* leaves


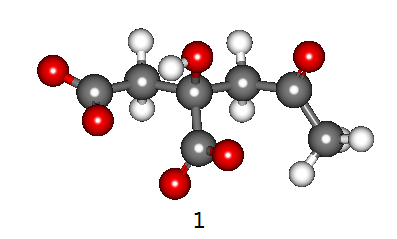

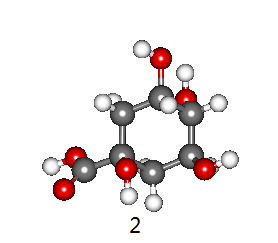

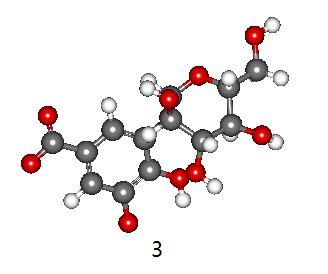

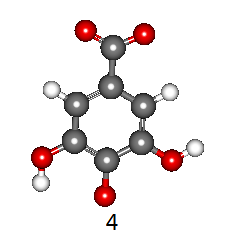


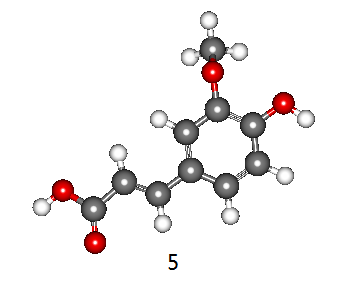

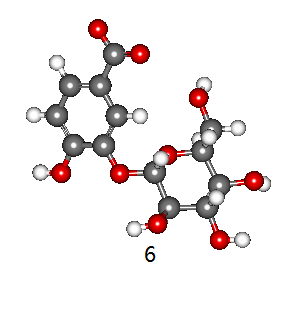

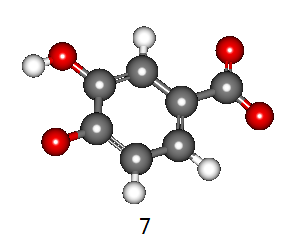

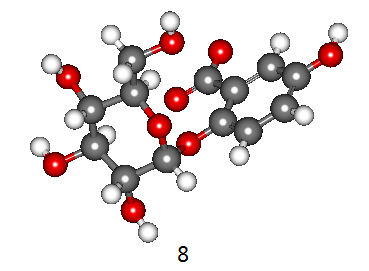


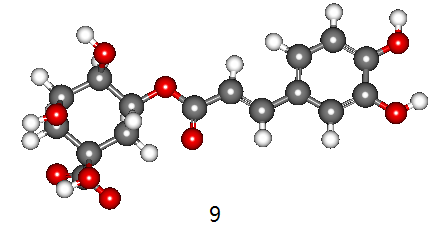

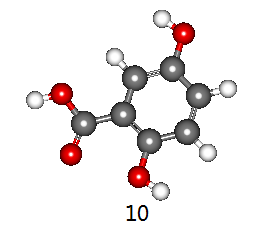


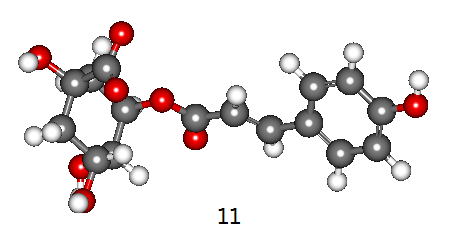

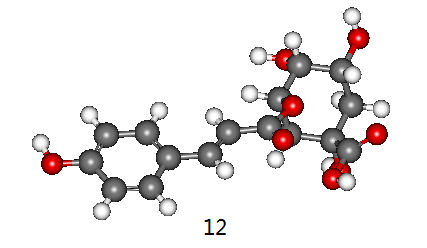

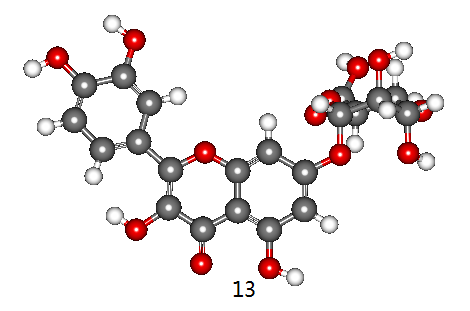

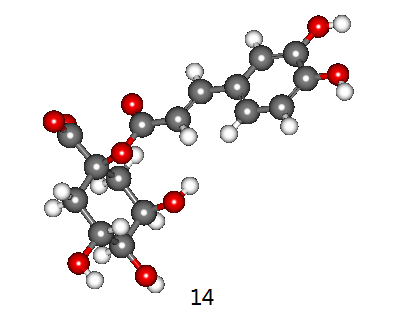


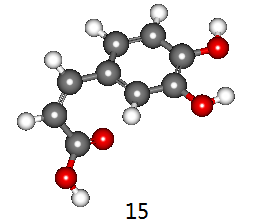

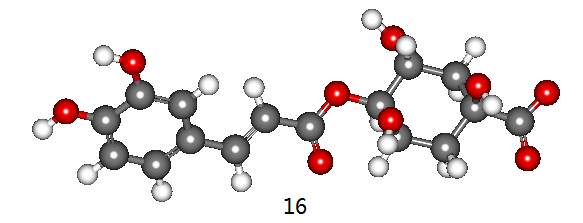

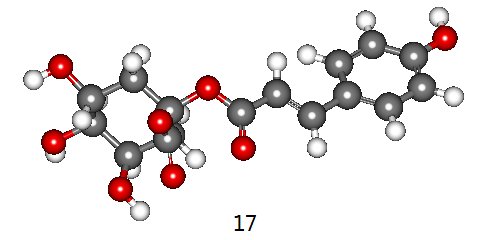

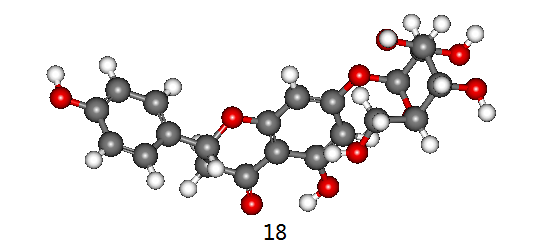


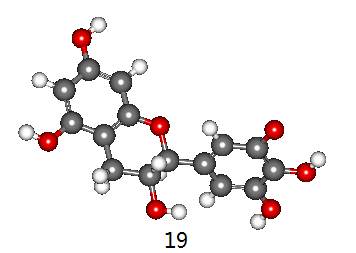

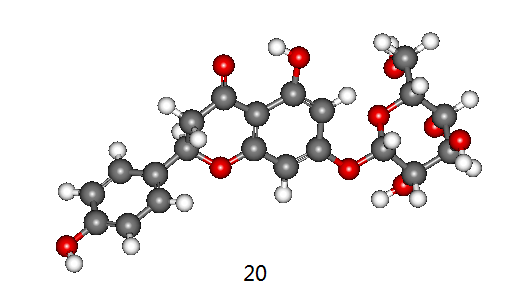


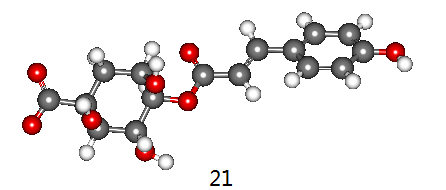

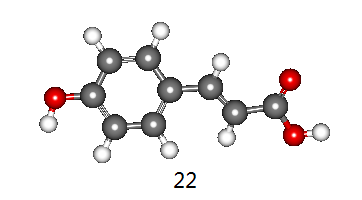

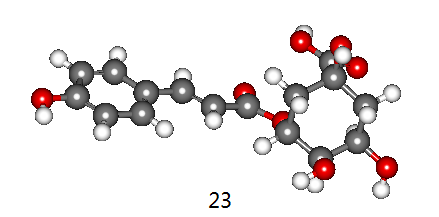

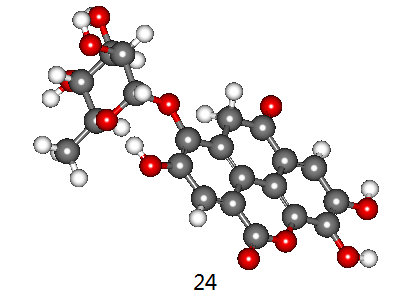


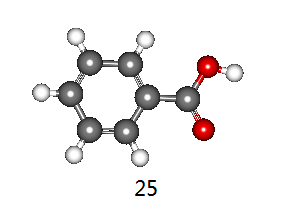

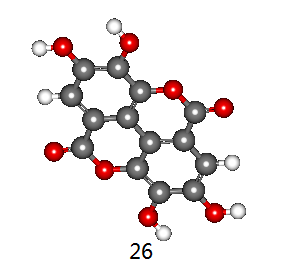


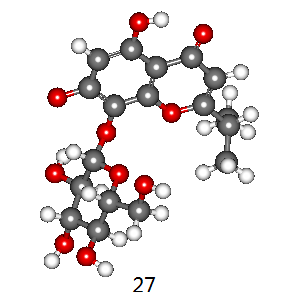

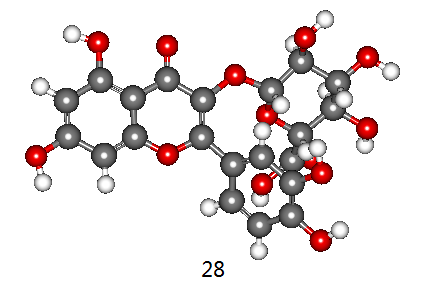


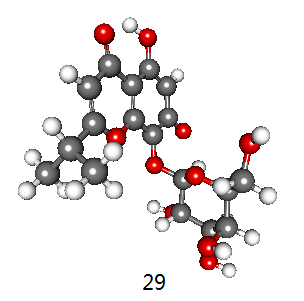

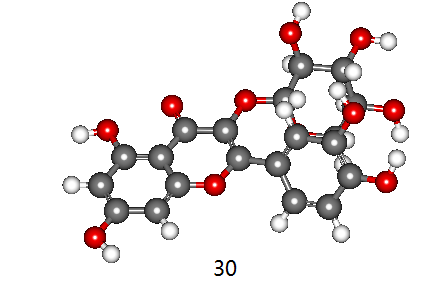


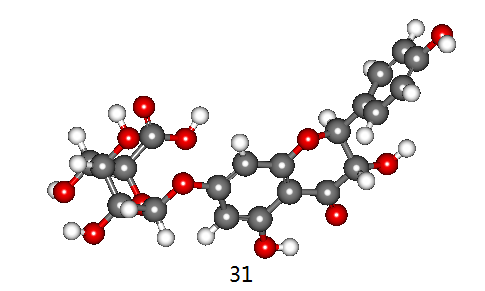

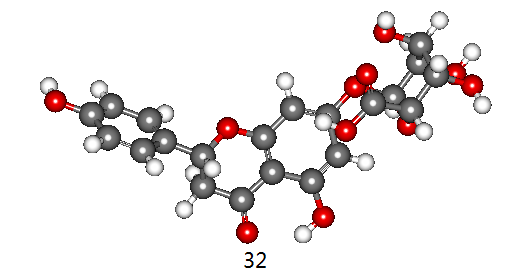


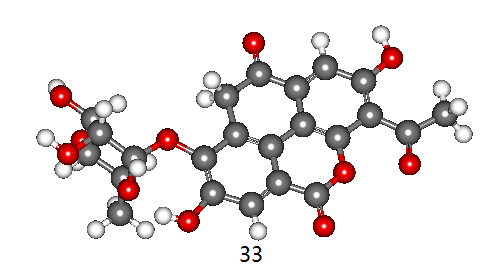

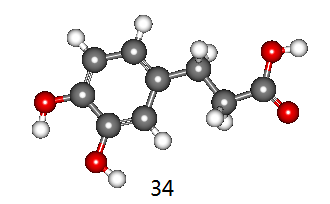


Structure of volatile components of *E. globulus* leaves


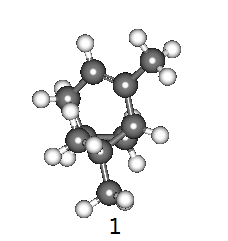

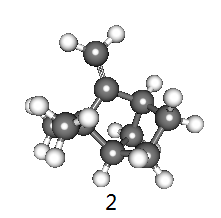

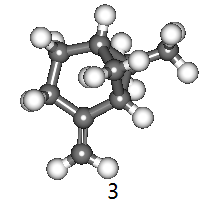

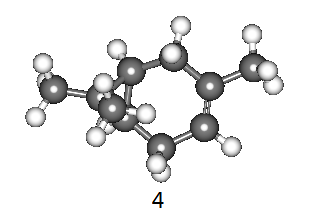

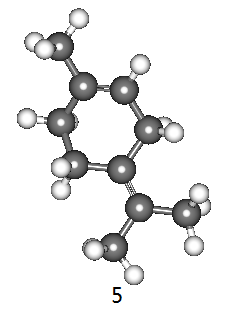

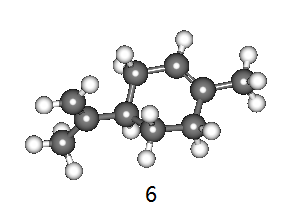

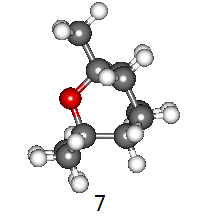

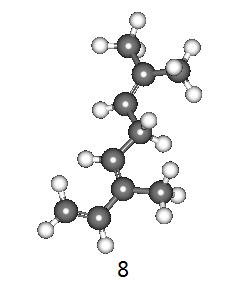

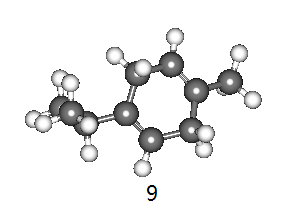

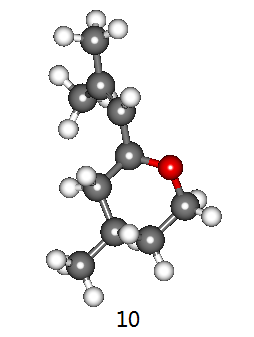

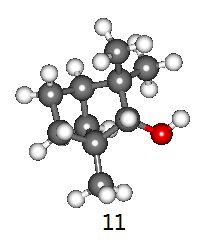

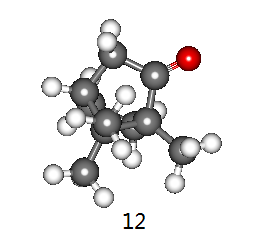


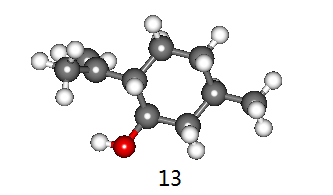

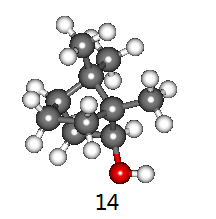

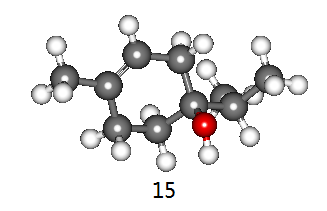

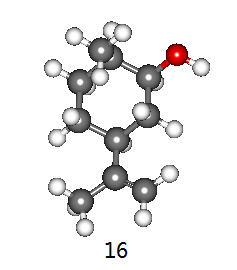

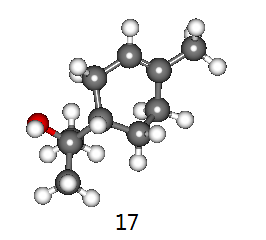

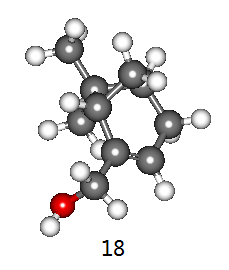

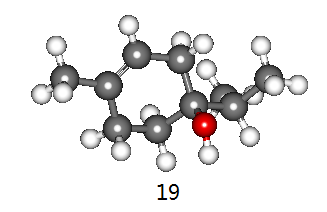

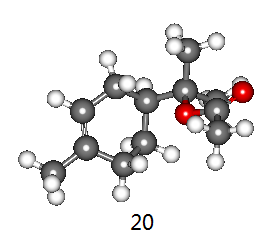


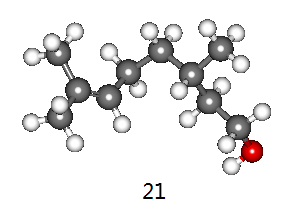

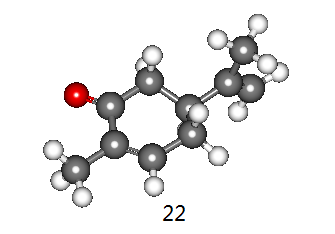

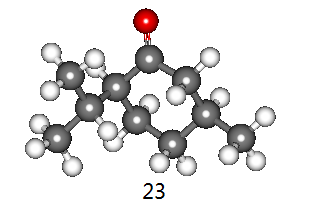

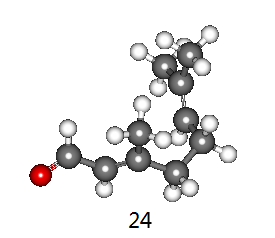


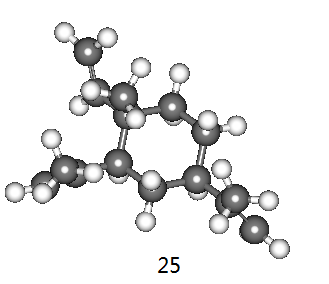

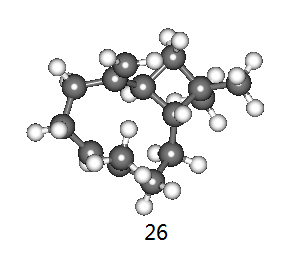

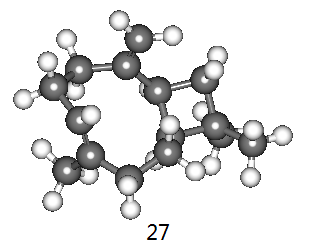

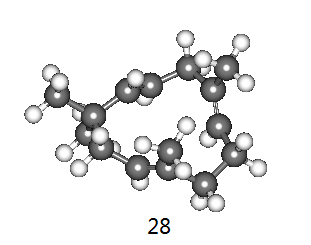


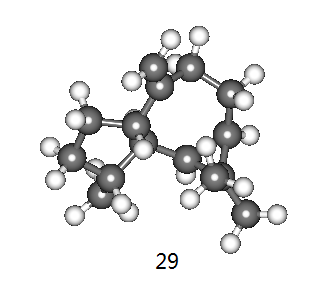

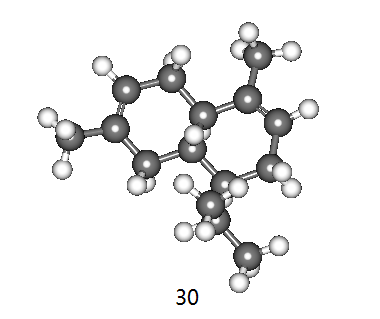


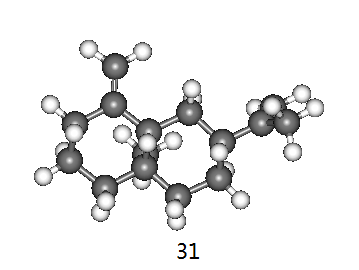

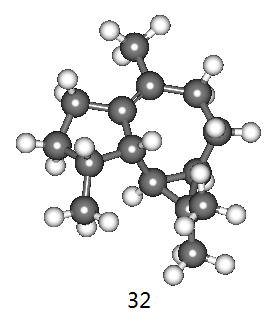

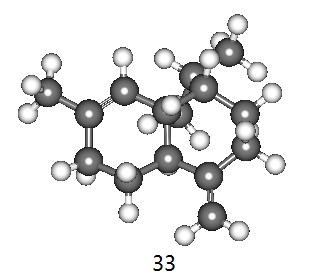

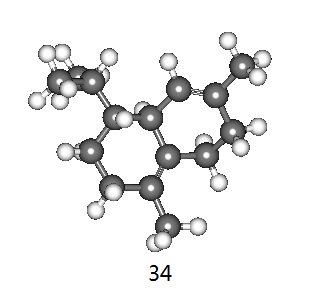


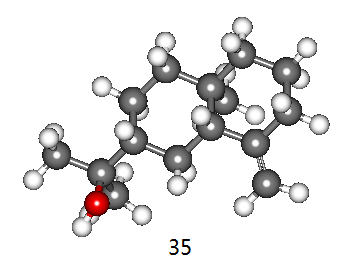

Supplement: Supplementary file 1 [file ijms-20-03875-s001.zip › ijms-541543-final sup/Supplement 2.docx]
